# Soundscape and ambient noise levels of the Arctic waters around Greenland

**DOI:** 10.1038/s41598-021-02255-6

**Published:** 2021-12-03

**Authors:** Michael Ladegaard, Jamie Macaulay, Malene Simon, Kristin L. Laidre, Aleksandrina Mitseva, Simone Videsen, Michael Bjerre Pedersen, Jakob Tougaard, Peter Teglberg Madsen

**Affiliations:** 1 Zoophysiology, Department of Biology, Aarhus University, Aarhus, Denmark; 2 Greenland Institute of Natural Resources, Nuuk, Greenland; 3 Greenland Climate Research Centre, Greenland Institute of Natural Resources, Nuuk, Greenland; 4 Polar Science Center, Applied Physics Laboratory, University of Washington, Seattle, WA USA; 5 Marine Mammal Research, Department of Ecoscience, Aarhus University, Roskilde, Denmark

**Keywords:** Environmental impact, Marine biology, Physical oceanography, Ecology, Biooceanography, Zoology

## Abstract

A longer Arctic open water season is expected to increase underwater noise levels due to anthropogenic activities such as shipping, seismic surveys, sonar, and construction. Many Arctic marine mammal species depend on sound for communication, navigation, and foraging, therefore quantifying underwater noise levels is critical for documenting change and providing input to management and legislation. Here we present long-term underwater sound recordings from 26 deployments around Greenland from 2011 to 2020. Ambient noise was analysed in third octave and decade bands and further investigated using generic detectors searching for tonal and transient sounds. Ambient noise levels partly overlap with previous Arctic observations, however we report much lower noise levels than previously documented, specifically for Melville Bay and the Greenland Sea. Consistent seasonal noise patterns occur in Melville Bay, Baffin Bay and Greenland Sea, with noise levels peaking in late summer and autumn correlating with open water periods and seismic surveys. These three regions also had similar tonal detection patterns that peaked in May/June, likely due to bearded seal vocalisations. Biological activity was more readily identified using detectors rather than band levels. We encourage additional research to quantify proportional noise contributions from geophysical, biological, and anthropogenic sources in Arctic waters.

## Introduction

In the last century, marine soundscapes have faced substantial and increasing contributions from anthropogenic sources due to increased human encroachment in the marine environment^1–4^. As a consequence, underwater noise levels are on the rise globally due to human activities such as shipping, seismic surveys, sonar, and construction^5^ and are predicted to continue to increase in the future^6^. Some regions have seen increases in low frequency ambient noise levels of 1–3 dB per decade in the 30–300 Hz range^7^ while activities such as seismic surveying and pile driving may periodically raise the noise floor by tens of decibel over hundreds of square kilometres^8–11^.

Several intergovernmental organisations have recognised anthropogenic underwater noise as an environmental pollutant with the potential for significant adverse effects on marine life^12–15^. Consequently, legislation aimed at regulating underwater noise has been passed, with underwater noise targeted most directly by the European Union Marine Strategy Framework Directive (descriptor 11)^16^. Paramount to managing any environmental pressure factor, such as underwater noise, is the ability to monitor its development in time and space. Such monitoring can inform managers of the magnitude of the problem, the relative contribution of individual sources and, most importantly, provide feedback on the effectiveness of mitigation measures.

The Arctic is a geographic region that is still relatively unaffected by anthropogenic noise pollution compared to other lower-latitude regions, which is mainly explained by extended seasonal periods of inaccessibility due to sea ice formation^14,17,18^. However, sea ice coverage in the Arctic is reducing rapidly^19^, with established and potential Arctic shipping routes now likely to stay open for longer each season, which will likely result in a marked shift in global shipping^20,21^. Further, the Arctic is rich in natural resources, it is home to unique wildlife, and its location is strategically important for the world’s superpowers and coalitions. Consequently, human exploitation, tourism and geopolitical interests are likely to increase shipping, survey activity, construction, and human presence in the Arctic region^22,23^ with increased noise pollution as an expected side effect. It is therefore critical to establish sufficient acoustic monitoring in the Arctic to track the predicted changes in ambient noise levels and thus allow for mitigation and legislation development on an informed basis^14,17,18^.

Monitoring acoustic habitats over large spatiotemporal scales can be performed using passive acoustic monitoring (PAM) where autonomous acoustic loggers are deployed to record for months or even years^24–27^. Each recording device may collect highly detailed data for a given location, such as noise, temporal and spatial abundance trends in vocalising animals and generalised biodiversity indices. However, it is a massive and costly undertaking to deploy enough devices to sufficiently cover entire ocean basins. Instead, large-scale estimation of noise and other ecological metrics from acoustic data can be attempted by generating models which are then validated against a subset of data samples. For example, acoustic indices^28^ may inform habitat modelling of the predicted habitat preference of different species^29^ and external metrics such as meteorological, hydrographic and AIS (Automatic Ship Identification System) data can be used to model noise levels^30^. While a modelling approach provides the potential for large-scale ecological inferences, the models generated are only as good as the data that have been used to validate and refine them. Thus, inherently, models require the collection of representative samples of acoustic data^31^, and these can comprise multiple long-term recordings over years, and thus terabytes of data, and present a daunting wealth of information to process. Ideally, every sound source is detected and classified and target sounds extracted for desired model outputs, for example to quantify anthropogenic or ambient noise levels or assess temporal distributions^32,33^ or estimating animal densities^34,35^.

For any large-scale monitoring project, the initial analysis stage is to characterise relevant soundscape metrics for the desired research outputs. Ideally, the more fully a soundscape is described (i.e. extracting noise metrics, identifying vocalisation of different species and sources of anthropogenic noise) the greater the ecological context and the larger the number of possible research outputs. However, currently such broadscale soundscape analysis represents an overwhelming analytical task for which comprehensive tools have not yet been fully developed. Calculations of noise levels are an example of soundscape metrics which are relatively straightforward and well established^36^, however, merely reporting an average, broadband long-term noise level for a given recording site provides limited information about the soundscape, its seasonal variation, and the nature and context of sound sources driving the overall levels. Here we provide the first large-scale picture of the noise levels in the waters around Greenland, seeking a hybrid approach to soundscape quantification using broadband noise metrics in concert with generalised detectors of certain sound types to reveal some of the sources of variation in broad-scale noise levels.

Specifically, we report PAM data from 26 deployments in five general areas around Greenland across more than 8 years (Oct 2011–Jan 2020) in order to quantify variations in broadband ambient noise levels and obtain baseline estimates for future comparisons. Additionally, we analyse the acoustic data using generic automated noise type detectors for quantifying broad-scale features of Arctic soundscapes and supplement our analysis with manual auditing to identify the dominant noise sources at each recording location. We demonstrate considerable variation in overall noise levels, document human sources of noise, and show that highly seasonal patterns exist in several areas.

## Methods

### Field sites and data collection

**Figure 1:**
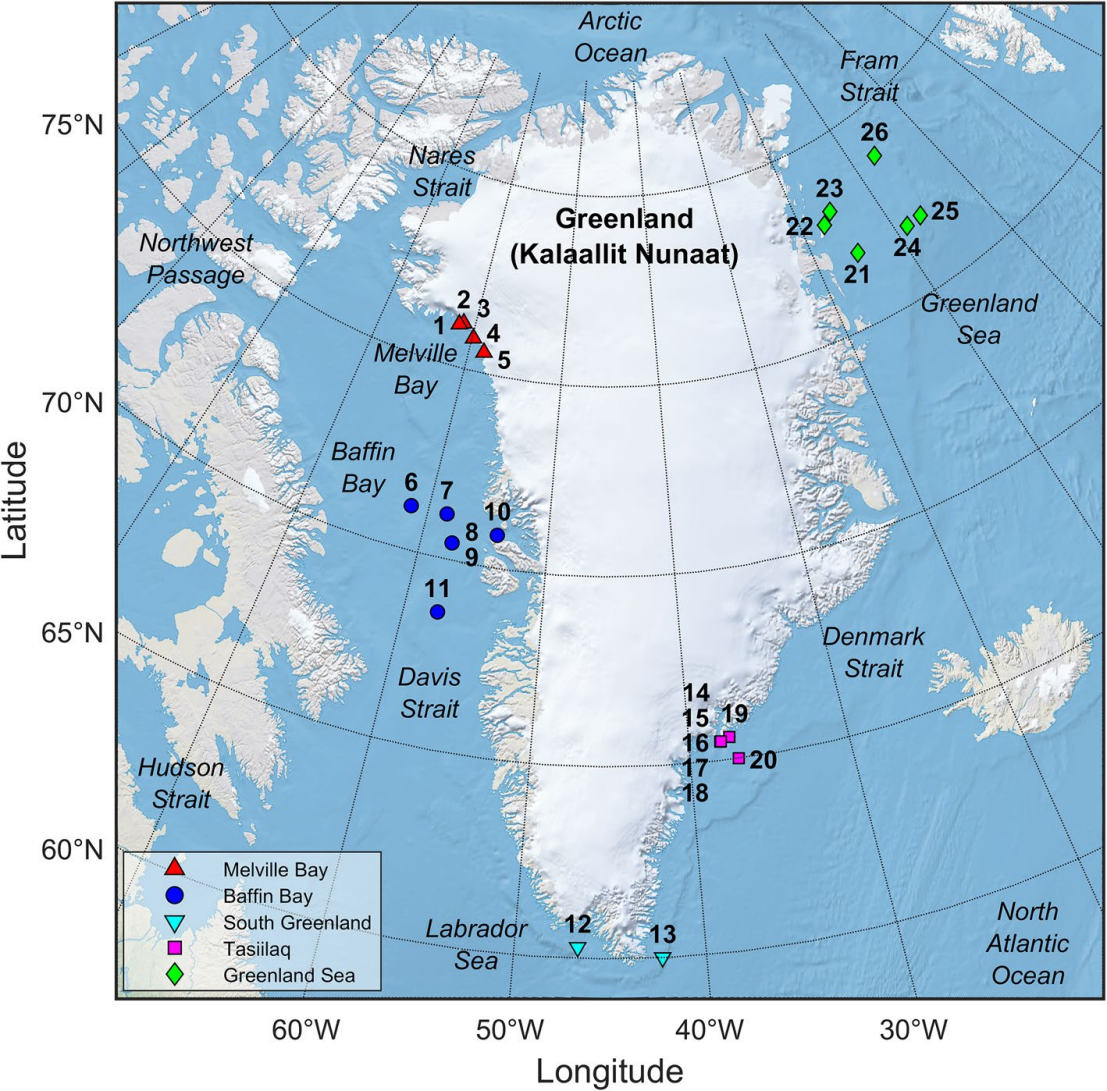
Map of recording locations. The stations (n = 26) are grouped into five regions. Melville Bay: red upwards pointing triangles, Baffin Bay: blue circles, South Greenland: cyan downwards pointing triangles, Tasiilaq: magenta squares, Greenland Sea: green diamonds. For stations 2 and 3, 8 and 9 and 14–18, only a single symbol is visible due to close proximity between locations. This figure was generated using MATLAB including functions such as readgeoraster, axesm and geoshow from the MATLAB Mapping Toolbox (Version 4.10, https://se.mathworks.com/products/mapping.html). The raster map data was obtained from the Natural Earth website (“Natural Earth I with Shaded Relief, Water, and Drainages”, scale 1:10 m, version 3.2.0, www.naturalearthdata.com).

The recordings were made between 2011 and 2020 off the coast of Greenland between 60.0° and 78.8° N and 6.7°–61.7° W at stations in the regions of Melville Bay, Baffin Bay, Qaqortoq and Prince Christian Sound in South Greenland, Tasiilaq in south-eastern Greenland, and the Greenland Sea (Fig. 1, details for each station are listed in Table 1).

**Table 1:**
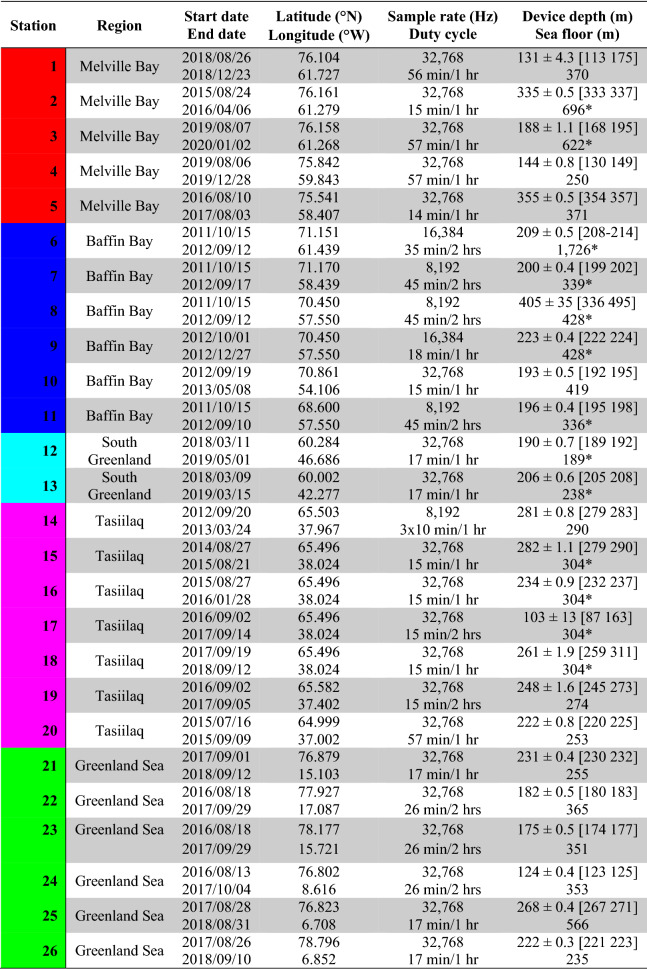
Overview of the 26 deployments and field sites.

Device depth is listed as mean ± SD with range in square brackets. (Note: Sea floor depths marked with asterisks (*) are not measured depths from this study, but stem from The International Bathymetric Chart of the Arctic Ocean Version 4.0^37^ that has a grid resolution of 200 × 200 m).

Recordings were made using Aural-M2 devices (Multi-Électronique (MTE) Inc., Rimouski, Quebec Canada) fitted with HTI-96-Min hydrophones (sensitivity of − 165 dB re. 1 V/µPa, flat frequency response from 2 Hz to 30 kHz, High Tech Inc., Long Beach, MS, USA) and operating at system peak clipping levels of 151 dB re. 1 µPa, sampling rates of 8192, 16,384, or 32,768 Hz (16 bit) and recording either at duty cycles of 10–45 min per hour or every second hour, or near continuously (Table 1). The acoustic loggers were moored to the bottom with a 300–600 kg anchor attached to an acoustic releaser (EdgeTech, PORT-LF or Teledyne Benthos865) with the parts tied together using Dynema line. On some moorings, a few metres of chain was attached to the anchor. Some moorings contained additional sensor equipment such as CTD instruments (e.g. Teledyne Acoustic Doppler Current Profilers and Sea-Bird Scientific SBE 37 SM, SBE 37 SMP and SBE 56 devices). Subsurface floats were attached at the top of the moorings and in several places along the line to keep the mooring straight in the water column. The acoustic recorders were attached from 15 m to several hundred metres from the anchor and 1.5–20 m from the top float with resulting recorder depths of 103–405 m (however, in a few cases strong ocean currents or large icebergs significantly affected the depth of the recorders by tens of metres, Table 1). Generally, the recorders were deployed for durations up to about a year to monitor seasonal variations although some were only deployed for a few months (Table 1). For the analysis, the first 24 h after deployment and last 24 h before recovery were ignored to exclude noise contributions from the vessel deploying the loggers.

### Analysis of noise levels

The noise analysis was performed using PAMGuard^38^, an open source toolbox for passive acoustic monitoring (version 2.01.04, www.pamguard.org). For each deployment, the noise was quantified as third octave levels (TOLs) at ANSI standard (base 2) centre frequencies^36^ ranging from 10 Hz up to 3.15, 6.3 or 12.5 kHz depending on sampling rate. Third octave bands were implemented using 6-pole Butterworth band pass filters in concurrent non-overlapping 10 s windows before computing the TOLs as the root mean square (RMS) levels after those filters. The TOLs subsequently served for computation of weekly and monthly levels. Further, the TOLs were used for estimation of decade levels in the bands from 10 to 100, 100–1000, and 1000–10,000 Hz by summation of the 10 s TOL (on a linear intensity scale). For the third octave bands only partially overlapping with the decade bands, the TOLs were scaled by the fraction of overlap before summation.

### Flow-noise and system self-noise

To identify if flow-noise was a potential problem, diel patterns of the computed TOLs were investigated via periodograms (for each station, TOLs were summarized as median hourly levels, the absolute FFT was computed (FFT size = number of hourly estimates), and the x-axis was scaled by Nyquist frequency and FFT size to convert to hours). Generally, a marked ~ 6/12-h signature, presumably indicating tidal flow, was identified in the periodograms, particularly at the 10 Hz band, which usually persisted at decreasing magnitude up to the 100–200 Hz third octave bands. More detailed analysis including noise level correction for flow-noise effects is outside the scope of this study.

Self-noise calibrations for the Aural-M2 recorders were not available, so the TOL distributions were also used for approximate self-noise estimation: Self-noise limitation was assumed if the exceedance levels^24^ L_99_ and L_90_ (where subscripted numbers indicate the percentage of measurements above each level) in each third octave band differed by less than 0.5 dB or if the lowermost width of violin plots exceeded a ratio of 0.05 relative to the maximum violin width. For such third octave bands, the self-noise was estimated as the L_99_ or minimum TOL, respectively, and a cubic polynomial function was fitted for illustration of the problem (see “Supplementary TOL Figures”).

### Analysis of noise types using PAMGuard detectors

For quantifying fluctuation of general noise types in the environment, we applied generic PAMGuard detectors that would search for tonal and transient noise events over different frequency scales.

Tonal sounds were detected using PAMGuard’s “Whistle and Moan Detector” (WMD) module^39^. The WMD module performs a number of noise reduction and thresholding processes on spectrogram data to effectively generate binary images which are then processed using a connected component analysis algorithm to detect any long, narrowband tonal signals^39^. Two different detector settings were applied for each recording. The first detector searched for tonal sounds across the frequency scaled spectrogram (FFT size 1024 bins, FFT overlap 512 bins and Hann window using PAMGuard’s default settings) from 1500 Hz up to the Nyquist frequencies i.e. 16,384, 8192 or 4096 Hz depending on sampling rates. This configuration was used to detect higher frequency tonal sounds such as toothed whale whistles, however the frequency resolution (sampling rate divided by FFT size) was insufficient for effectively detecting lower frequency tonal sounds, such as typical baleen whale vocalisations. A second lower frequency WMD module was therefore implemented for the detection of tonal events below 1500 Hz. Data was low pass filtered (4-pole Butterworth, 1500 Hz cut-off), decimated to 3000 Hz sample rate, converted to a low frequency spectrogram (FFT length 512 bins, 256 bins overlap and Hann window) and processed using a second instance of the WMD module. Both WMD algorithms used default PAMGuard settings.

Transients were detected using PAMGuard’s “Click Detector” and “Ishmael Energy Sum” (IES) modules. The PAMGuard click detector detects transients based on the amplitude difference between sample-by-sample moving average measures of a signal search window and a noise estimate window within a specified filter band (4-pole Butterworth 3000 Hz high pass). The averaging interval of the signal window is significantly less than that of the noise window (the length difference is of magnitude 4 or 5 depending on whether the signal level is below or above threshold, respectively) and so the detector rapidly tracks changes in amplitude whilst the noise level changes much more slowly over time. A click is then detected and saved if the signal has a predefined threshold (12 dB) above the noise, which was chosen following exploratory analysis of sequences with and without biosonar signals. The click detector module is generally efficient at detecting short transients such as echolocation clicks, however, it is unsuited for longer impulsive sounds, such as seismic airgun sounds. Therefore, a second detector searched for longer impulsive transients below 1500 Hz with characteristics similar to seismic airgun sounds. The IES module is similar to the click detector module, however, the basis for the signal and noise level is the summed energy within FFT data of a spectrogram rather than continuous acoustic samples^40^. The Ishmael detector was run on the same spectrogram data as the low frequency WMD detector and calculated the energy sum signal and noise levels between 0 and 600 Hz, registering a detection if the signal-to-noise level exceeded 8 dB (this threshold was chosen to reduce the number of missed seismic pulse detections while preventing unmanageably sized detector outputs resulting from false positives). The IES outputs from the seismic detector were post-processed in MATLAB (R2020a, MathWorks, Natick, Massachusetts, USA) using custom written scripts in order to classify specific events as seismic activity. Given that detailed detection and mapping of seismic activity in the North Atlantic and Arctic Ocean was outside the scope of this study, the seismic classifier was kept relatively simple and relied on the knowledge that some seismic surveys in Greenland and the North Atlantic were known to have operated at stable inter-pulse intervals (IPIs) of 12, 14 and 18.5 s. Consequently, some seismic surveys are likely to have been missed. First, all possible IPIs were computed between all transient detections made within each wav-file (26–135 min durations depending on wav file storage settings and duty cycle, Table 1). Seismic airgun activity was then classified as having been present, if the IPI mode was within a 400 ms tolerance window, 12 ± 0.2 s, 14 ± 0.2 s or 18.5 ± 0.2 s (a 400 ms window was chosen to accommodate IPI differences stemming from temporal variation of signal-peak occurrence within each reverberant signal) and if a minimum of 15 consecutive pulses were detected within 600 s.

The TOL and noise type detections were summarised for each station in circular plots representing durations of 1 year (“Supplementary Soundscape Figures”). For recordings exceeding this duration, only the first 365 days were plotted.

### Identification of broad-scale events

Selected manual auditing was performed in order to verify automatic detectors and to identify main noise source contributors at each recording station. The sheer size of the data set precluded inspection of every single wav-file. Instead, datagrams including long-term spectral averages, TOL noise profiles, concatenated spectra of clicks and the WMD detector outputs were plotted in PAMGuard allowing a manual analyst to visualise months of noise and detector data. Events of interest that were visible on the datagrams were selected and the manual analyst then used PAMGuard’s data visualising tools to inspect spectrograms and detector outputs at much finer temporal scales and, if necessary, listen to raw acoustic data.

Given that maximum sampling rates were ~ 32 kHz, the Nyquist frequency becomes 16 kHz, thus the recordings were unsuited for investigating toothed whale biosonar, which generally operates in frequency ranges of many tens of kilohertz^41^. Only sperm whale clicks^42^ are likely to contain considerable energy below this 16 kHz limit. Nevertheless, toothed whale clicks were often detected in the recordings, which is likely due to the recording artefact called aliasing, where energy above the Nyquist frequency folds down into the recorded spectrum, thereby making click detections possible in the absence of proper low pass filters, but likely to different degrees for different species. Narwhals produce more low-frequency clicks than belugas at similar source levels^43^, so narwhal click energy will more likely fold down below the Nyquist frequency at detectable levels. Belugas, however, could still be detected by the WMD module, and subsequently identified for events containing bird-like vocalisations^44^. Very low click repetition rates (~ 0.5–2 clicks per s) without coinciding detections of tonal communication calls were assumed to stem from sperm whales^42^, whereas species identification for other echolocation events was sometimes possible based on simultaneous whistle detections.

### Code availability

The PAMGuard software is open source and freely available at www.pamguard.org. In PAMGuard, we used built-in modules for all parts of the analysis using settings as described above; detailed settings for each module can be found in the attached xml files in the “Supplementary Information”.

The PAMGuard-MATLAB library was used to extract detection results from bespoke PAMGuard detection files into MATLAB (www.pamguard.org).

For the seismic activity classification, we used built-in functions from MATLAB. For making the deployment map in Fig. 1, we also used functions such as *readgeoraster*, *axesm* and *geoshow* from the MATLAB Mapping Toolbox (Version 4.10). The raster map data was obtained from the Natural Earth website (“Natural Earth I with Shaded Relief, Water, and Drainages”, scale 1:10 m, version 3.2.0, www.naturalearthdata.com).

## Results

Recordings were made at 26 stations (Table 1, Fig. 1) in the years between 2011 and 2020. The total combined deployment duration (counting only time intervals included in the analysis) summed to 20 years and 298 days of deployment. Because of duty-cycling, the combined recording duration amounted to 6 years and 149 days, which produced 16.9 TB of data.

### Ambient noise levels

**Figure 2:**
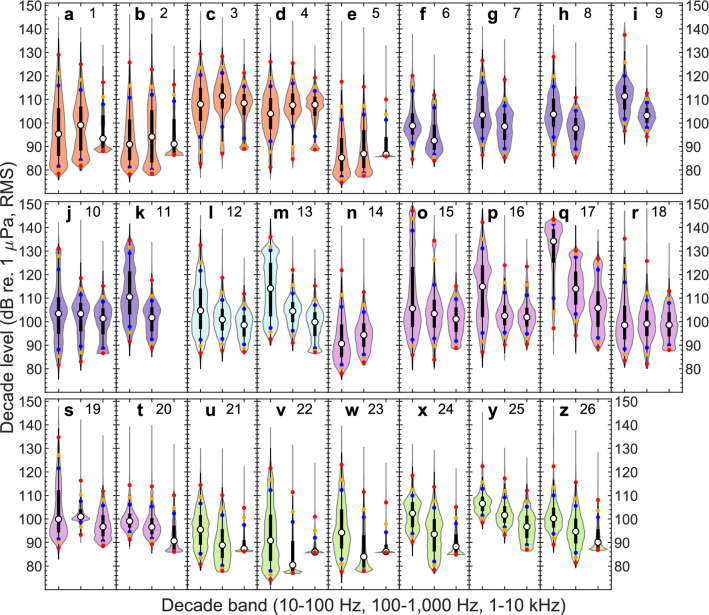
Decade level distributions (asynchronous) for the 26 recording stations. (**a**–**z**) Distributions of 10 s decade level estimates are shown for each station in the decade bands 10–100 Hz (VLF), 100–1000 Hz (LF), and 1–10 kHz (MF, only computed when sampling rates were sufficiently high). The decade level distributions are depicted as violin plots overlaid with box plots (interquartile range: thick black lines; whiskers: thin black lines). Exceedance levels are shown as coloured dots (L_50_ or median: white; L_90_ and L_10_: blue; L_95_ and L_5_: orange; L_99_ and L_1_: red). Stations numbers and region colours (Melville Bay: red, Baffin Bay: blue, South Greenland: cyan, Tasiilaq: magenta, Greenland Sea: green) as in Table 1 and Fig. 1.

The ambient noise was computed as third octave levels (TOLs) and also summarised as decade levels (Fig. 2) for the decade bands at 10–100 Hz (very low-frequency, VLF), 100–1000 Hz (low-frequency, LF), and 1–10 kHz (mid-frequency, MF). The “Supplementary Information” contain TOL distribution plots for each deployment and monthly TOLs summarised in tables as exceedance levels (L_1_, L_5_, L_10_, L_25_, L_50_, L_75_, L_90_, L_95_, and L_99_) for each of the 26 stations.

The completely flat noise level distribution bottoms and highly similar L_50_, L_90_ and L_99_ values in several plots (e.g. Figure 2b,e,v,w) show that ambient noise levels, mainly in the MF band, were often below the self-noise level of the acoustic logger (for details see the “Supplementary TOL Figures”), which results in overestimation of noise levels. Because of these self-noise limitations and low-frequency flow-noise observed mainly at the 10–200 Hz third octave bands, the decade band most representative of genuine ambient noise levels is likely the LF band, which will therefore be of main focus.

In Melville Bay (Fig. 2a–e), the recordings collected in late summer 2019 (Fig. 2c,d) showed considerably elevated noise levels compared to those collected between 2016 and 2018 (Fig. 2a,b,e) with full-deployment median levels in the LF decade band ranging from 108 to 111 and from 87–99 dB re. 1 µPa (RMS), respectively.

In Baffin Bay, the full-deployment median LF levels varied between 93 and 103 dB re. 1 µPa (RMS) (Fig. 2f–k). All Baffin Bay recordings, except station 10, were collected at low sampling rates (Table 1) so only the VLF and LF decade bands were computed.

In South Greenland, the median LF levels were 101 and 104 dB re. 1 µPa (RMS) (Fig. 2l,m). At Tasiilaq, median LF levels ranged from 95 to 114 dB re. 1 µPa (RMS) (Fig. 2n–t). Station 17 stands out among the 26 stations with the highest VLF and LF levels, however, considerable depth changes (Table 1) and extensive signal clipping were observed in the recordings.

**Figure 3:**
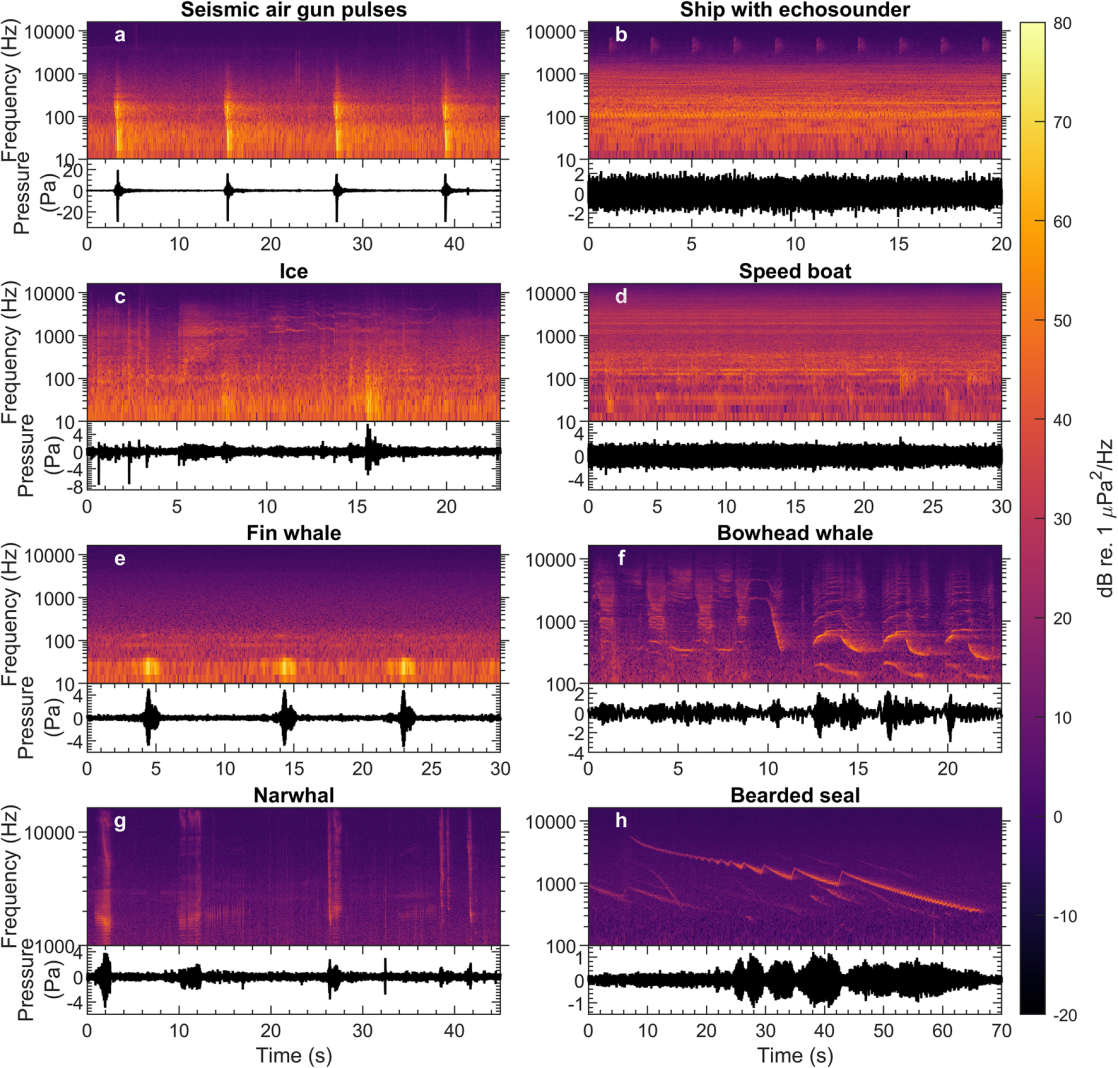
Sound source examples illustrating frequency contours and waveforms. (**a**) Seismic survey pulses (station 23). (**b**) Ship with active echosounder (station 21). (**c**) Ice noise showing both transient and tonal signals (station 21). (**d**) Speed boat (station 18). (**e**) Fin whale calls (station 25). (**f**) Bowhead whale calls (station 26). (**g**) Narwhal echolocation clicks and calls (station 26). (**h**) Bearded seal call showing the characteristically down-sweeping frequency contour of this species (station 21). All sources are recorded at unknown range. All signals were sampled at 32,768 Hz and spectrograms were made using an FFT size of 4096, a 4096-sample Hann window and a 2048 sample overlap. Note the logarithmic y-axes, which allow for better visualisation of low-frequency energy in the spectrograms.

In Greenland Sea, median LF levels varied between 84 and 102 dB re. 1 µPa (RMS) (Fig. 2u–z). As in Melville Bay, the MF decade levels in Greenland Sea were generally close to or below the system self-noise. Overall, stations in Greenland Sea recorded the lowest noise levels followed by Melville Bay. However, during the manual audit it was noted that seismic airgun activity could result in a constantly raised noise floor (mainly below ~ 1 kHz), as reverberations did not appear to plateau at natural ambient levels in-between airgun pulses (e.g. Fig. 3a).

### Decade levels versus 63, 125 and 2000 Hz third octave bands

For the purpose of comparison with the two Marine Strategy Framework Directive indicator bands centred at 63 and 125 Hz^45^ and the 2 kHz band used in the BIAS project^27^, the three TOL distributions were plotted in the same manner as the decade levels (Supplementary Fig. S1). Overall, the three decade bands and three third octave bands show highly similar distributions (for crude comparison of absolute levels between the three third octave bands and the decade bands they each fall into, one should compensate by 10log_10_ of the bandwidth ratios, i.e. 8, 15, and 13 dB respectively). The main differences are found between the VLF decade band and the 63 Hz third octave band where VLF bands are more top-heavy (e.g. stations 11–13, Fig. 2, Supplementary Fig. S1), which potentially relates to flow-noise.

### Noise source examples and calendar plots

**Figure 4:**
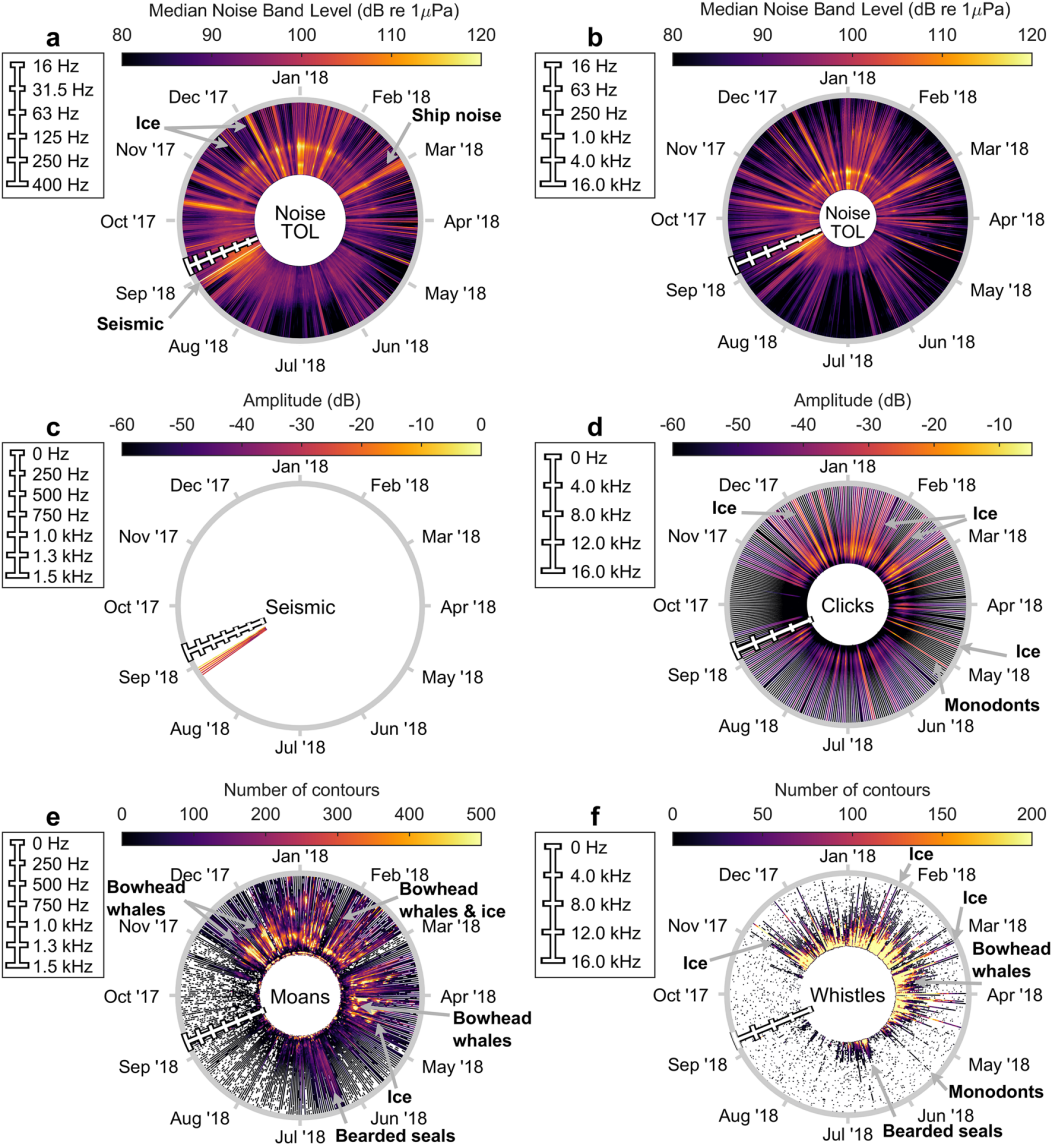
Example of noise and detector outputs for soundscape characterisation from station 26 plotted in 10-min resolution. Circles represent durations of 1 year. The position of the frequency scale bar in late August represents the start of the recording. (**a**, **b**) Daily median third octave levels (TOLs) are colour-coded relative to amplitude and shown for the third octave bands each centred at frequencies between 10 and 400 Hz (**a**) and over the full range from 10 Hz to 12.5 kHz (**b**) on a logarithmic axis. (**c**) Seismic survey detections are shown as the median frequency content (< 1.5 kHz) of the detected events. (**d**) The median spectral content for all transients detected. (**e**) The contours for all detected low-frequency tonal signals (moans, < 1500 Hz) are plotted on top of each other and colour-coded as number of signals overlapping in each frequency bin (5.86 Hz width). (**f**) The contours for all detected high-frequency tonal signals (whistles, 1500–16,384 Hz) are plotted on top of each other and colour-coded as number of signals overlapping in each frequency bin (32 Hz width).

A collection of sound source examples is presented as spectrograms and waveforms in Fig. 3 to illustrate the variety of noise sources and frequency contours of their signals. An example of an annual soundscape representation is shown for station 26 (Fig. 4), and similar figures are found for all stations in the “Supplementary Information”. The low-frequency noise is plotted for the TOL bands each centred at frequencies between 10 and 400 Hz (Fig. 4a) along with TOLs across the full frequency range from 10 Hz to 12.5 kHz (Fig. 4b). In both TOL plots, one of the main highlights occur in early September, which coincides with detections of seismic survey activity (Fig. 4c). Several other distinct events with durations up to a few days are dispersed over the year, where some of the apparent sources seem to be ice, weather and shipping. The click detections for station 26 were numerous over the entire year although with the highest number of detections occurring from late autumn to early spring (Fig. 4d). Some of these are echolocation clicks of toothed whales, although many are ice or unidentified transient-like noises. Low-frequency tonal sounds (moans) were detected year round at station 26, but most noticeably between November and May where bowhead whale (*Balaena mysticetus*) vocalisations were frequently identified (Fig. 4e,f). Bearded seals (*Erignathus barbatus*) were highly vocal during summer with calling activity peaking around May/June (Fig. 4e,f). Ice noise and bowhead whale calls were frequent among the high frequency tonal (whistle) detections, but also monodonts and various seal calls were identified.

### Seasonal noise variation

**Figure 5:**
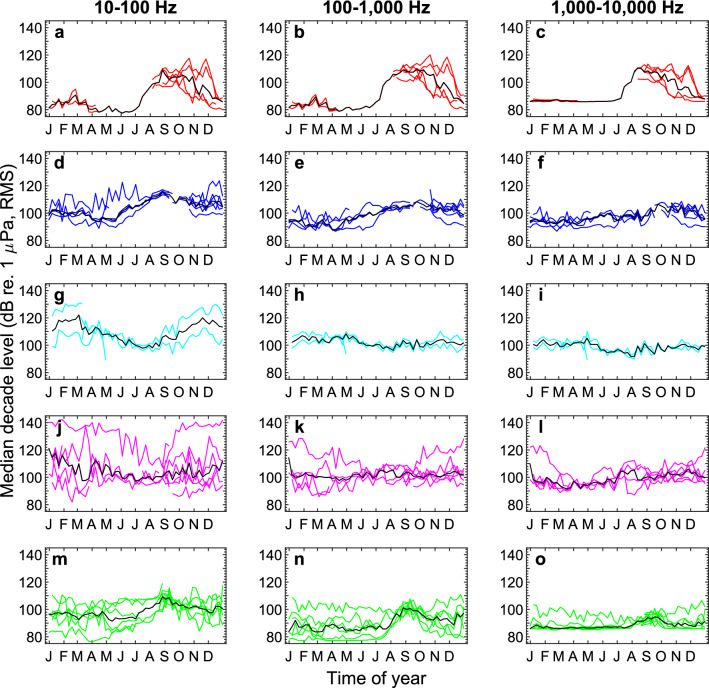
Seasonal and regional (asynchronous) variation of ambient noise levels summarized as weekly median levels. The weekly median decade levels are plotted for each station for each of the five geographic regions: Melville Bay (**a**–**c**), Baffin Bay (**d**–**f**), South Greenland (**g**–**i**), Tasiilaq (**j**–**l**), and Greenland Sea (**m**–**o**) in the decade bands 10–100 Hz (VLF, first column), 100–1000 Hz (LF, second column), and 1–10 kHz (MF, third column). Black lines show the median for all data in each subplot. Note that the transition from December to January results in an additional line being plotted for each station. Self-noise has likely affected some noise estimates particularly in the MF band. For recordings exceeding a duration of 1 year, some weekly medians are based on data from the same weeks in different years. Region colours as in Fig. 1 (Melville Bay: red, Baffin Bay: blue, South Greenland: cyan, Tasiilaq: magenta, Greenland Sea: green).

Analysis of seasonal noise variation showed a consistent pattern for Melville Bay, Baffin Bay, and Greenland Sea (Fig. 5a–f,m–o). From January to June, decade levels were generally lower compared to the rest of the year and often relatively stable, although some stations showed considerable variation. July/August marked an increase in noise levels in all decade bands that generally peaked in September/October. By December, the decade levels had decreased and were comparable to the levels in January to June (Fig. 5a–f,m–o).

In South Greenland, the seasonal noise variation was highly similar between the two stations except for the VLF band, where initially similar levels from March to August were followed by a ~ 20 dB difference in the period from September to March (Fig. 5g–i).

At Tasiilaq, seasonal patterns were less obvious compared to other regions although noise variation seemingly decreased during June to August in the VLF and LF decade bands and around April in the MF band. Station 17 recorded exceptionally high levels (Fig. 5j–l).

### Detection counts and seasonal variation

**Figure 6:**
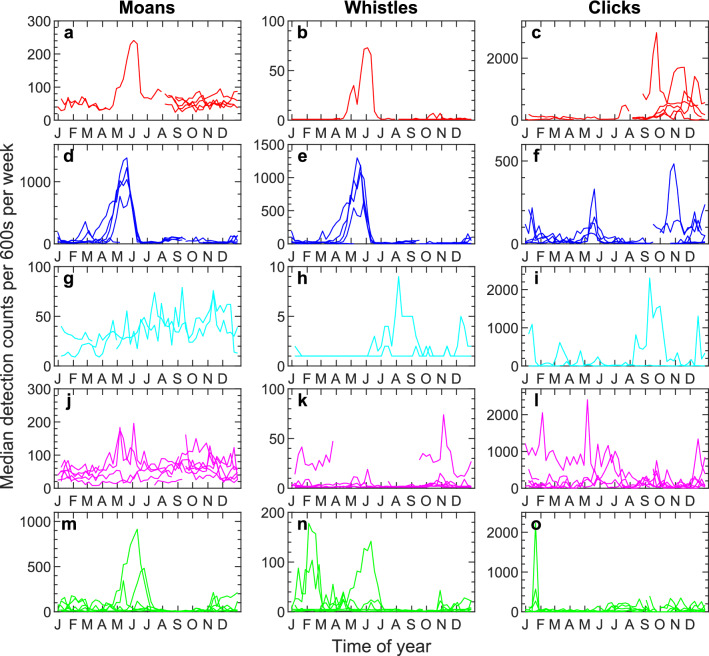
Seasonal and regional (asynchronous) variation of median detection counts. The first column show 0–1500 Hz tonal detector counts (moans). The second column show tonal detector counts (whistles) in the range from 1500 Hz to the Nyquist frequency. The third column show transient detector counts (clicks). The spikes in moans/whistles around May/June are likely bearded seal calls. Note that the variation in these sounds does not correspond to variations in decadal levels in Fig. 5. Region colours as in Fig. 1 (Melville Bay: red, Baffin Bay: blue, South Greenland: cyan, Tasiilaq: magenta, Greenland Sea: green).

There were distinct temporal variations in the detector outputs for Melville Bay, Baffin Bay, and Greenland Sea (Fig. 6a–f,m–o). Both the low frequency (moans, < 1500 Hz) and high frequency (whistles, 1500 Hz to Nyquist frequency) tonal detections show consistent and considerable increases in median detection rates from May to July, driven in part by high levels of vocal activity form bearded seals. It should be noted that these temporal patterns in detections correlate poorly with the temporal patterns in the decadal noise levels in Fig. 5. Temporal tonal patterns were less apparent for the stations in South Greenland and Tasiilaq (Fig. 6g–l), compared to the other three areas.

The click detector mainly triggered during toothed whale echolocation or ice noise, however, ship noise, mooring noise and weather were also among the detections (“Supplementary Soundscape Figures”). Click detector outputs did not yield similarly clear seasonal patterns as the tonal detectors (Fig. 6), although detection peaks due to ice in November and December were seen for several stations. In Baffin Bay, toothed whale echolocation resulted in a distinct peak for station 6 in May (Fig. 6f) and sperm whale clicks produced several detection peaks in South Greenland (Fig. 6i, “Supplementary Soundscape Figures”).

## Discussion

Long-term PAM programmes are needed for quantifying natural variation and changing underwater sound levels in relation to human activities. Our large-scale noise monitoring program in Greenland is one of the first of its kind, covering the deep waters off coastlines across the world’s largest island, and was motivated by an ambition to better understand Arctic soundscapes and to document underwater noise levels in this rapidly changing part of the world.

We present ambient noise levels from five regions around Greenland recorded in the period 2011–2020 along with observations of broad-scale soundscape characteristics and seasonal variation. The data were collected over different months and years and at varying ranges from the coast and from each other (Fig. 1, Table 1), which must be kept in mind when interpreting the results. Differences in oceanographic conditions have likely existed between stations, and variations in acoustic propagation phenomena, like the Arctic sound channel^46^, will likely have influenced detection range abilities and noise estimates. In addition, ambient noise levels, particularly in Melville Bay and Greenland Sea, were so low for extended periods that self-noise of the recording setup prevented reliable estimates of even the median ambient noise level at several third octave bands in the kilohertz frequency range (“Supplementary TOL Figures”). In those cases, the median noise levels are therefore overestimated to an unknown degree. Similarly, low-frequency flow-noise, which was mainly identified below 100–200 Hz, results in unknown overestimation of ambient noise levels at low frequencies. Therefore, if future investigations find that anthropogenic sources raise the noise levels in those bands compared to the values reported here, then noise pollution impacts will be underestimated.

In early recordings from Greenland Sea, McGrath^47^ observed monthly mean TOLs in the period October 1972–June 1973, in the 50, 100, and 400 Hz bands, ranging between 90 and 102 dB re. 1 µPa. More recently, Ahonen et al*.*^48^ collected 4 years of data in the Fram Strait at the same site between 2008 to 2014 and reported annual median TOLs in the 50 Hz, 63 Hz, 125 Hz and 500 Hz bands that ranged from 84 to 97 dB re. 1 µPa. These long-term median noise levels are similar to observations at lower latitudes in the North Atlantic, where Merchant et al*.*^49^ reported median TOLs in the 63, 125, 250 and 500 Hz bands that ranged from 91 to 96 dB re. 1 µPa in the North Sea, 79–95 dB re. 1 µPa in the Southern North Sea and 93–97 dB re. 1 µPa in the Celtic Sea. In our study, the median TOLs in the 50–500 Hz bands ranged from a minimum of 69 to a maximum of 103 dB re. 1 µPa, RMS (excluding station 17, see “Supplementary TOL Figures”), thus largely overlapping with previous Arctic and North Atlantic observations, although considerably quieter levels are reported here particularly for stations in Melville Bay and Greenland Sea. There is considerable seasonal variation of the ambient noise in the three northernmost regions (Melville Bay, Baffin Bay, and Greenland Sea) consistently increasing in the period from July/August to October/November by approximately 10–30 dB, compared to the rest of the year, with the highest increases occurring in the VLF and LF decade bands (Fig. 5). It can be difficult to determine the source of large variations in noise levels from TOL measurements alone, prompting us to employ a number of generic detectors (Fig. 4) to investigate the influence of environmental, anthropogenic and biological sources to the recorded soundscapes.

Arctic soundscapes are often characterised by exceptionally large seasonal noise variation that strongly depends on sea ice^50^. The most quiet ice-covered conditions can be about 20 dB lower than the lowest noise levels in the ice-free sea whereas ice-movements and rapid cooling of ice sheets can result in noise levels 20 dB higher compared to quiet open water conditions^50^. Both transient and tonal detectors were capable of detecting ice noises (Fig. 4, “Supplementary Soundscape Figures”), which highlights that ice produces a broad range of sounds^51^. In Melville Bay, Baffin Bay, and Greenland Sea, the highest noise levels and number of tonal detections are during summer months and early autumn, which correspond with the minimum extent of the Arctic sea ice^19^, whereas the lowest levels were seen during winter and spring (Fig. 5). Thus, higher noise levels occur during times of open waters in those areas; one explanation for this is that ice movements, alternatingly warming and cooling sea ice and wave-generated noise may be dominant noise sources in times of semi-open and open water, respectively^50^, where drifting icebergs may also contribute to the soundscape^52^.

However, increasing open water is also expected to lead to significantly higher levels of anthropogenic noise with shipping and seismic surveying in late summer and autumn. Our seismic detector mainly identified seismic activity during August and October, and additional events were identified from June to November during the manual audit, which closely matches the time where annual noise levels reach peak levels in Melville Bay, Baffin Bay and Greenland Sea. In several recordings, particularly from Greenland Sea, seismic pulses were followed by long-lasting reverberation that did not plateau at natural background noise levels in-between pulses thus producing a constantly raised noise floor in the environment particularly at frequencies below 1 kHz, similar to previous observations^8^. This documents that seismic surveys may dictate the ambient noise levels even during the time of year when natural noise levels are at their highest.

In Melville Bay and Baffin Bay, seismic activity has been shown to significantly raise ambient noise levels over the entire period from August to mid-October with negative consequences for the active space of acoustically communicating marine mammals^8^. In the Chukchi and Beaufort Seas, seismic airgun noise has been detected over substantial parts of the open water season in summer and fall and has been estimated to raise the average spectrum level by 2–8 dB^53^. Seismic activity detection was not the main focus in this study, and it should be noted that the detector and classifier used in this study were relatively simple and missed some seismic surveys (e.g. missed events were observed for stations 8–9, 11–14 and 25 during the manual audit, see annotations in “Supplementary Soundscape Figures”). Future noise studies would benefit from access to databases with detailed information about survey ship paths and airgun firing patterns for optimization. Off Tasiilaq and in South Greenland, shipping and small boat traffic was observed on several occasions during the manual auditing process and could at times also be highly dominant noise sources even at high frequencies (Fig. 3b,d).

The arctic marine environment hosts a multitude of soniferous species, thus it might be expected that biological sources contribute significantly to ambient noise levels. Our analysis did not include species classification among the tonal and transient detections, so the following observations stem from the manual audits of subsections of the data. Bowhead whales were identified in numerous recordings mainly during winter and spring (Fig. 4, “Supplementary Soundscape Figures”), which matches other observations for this species^48,54^. Bearded seal calls were commonly heard on many of the stations in spring and early summer with call activity peaking in May/June and markedly decreasing thereafter (Fig. 4, “Supplementary Soundscape Figures”). In Baffin Bay and Davis Strait, bearded seal vocalisations have been identified from January to June with call activity peaking in April to June^55^ which is similar to Greenland Sea where calls are detected from February to July with peak activity in May and June^56^. Bearded seal calls were readily detected by the moan and whistle detectors (e.g. stations 5–8, 10, 15, 17, 21, 25, 26, see “Supplementary Soundscape Figures”) and are likely important contributors to the seasonal tonal detection peaks observed from April to June in Melville Bay, Baffin Bay and Greenland Sea (Fig. 6a,b,d,e,m,n). Fin whale calls were also identified in some recordings (Fig. 3e). Fin whales are known to be highly vocal from September/October to February/March in the Fram Strait^48^ and from June to December in the Davis Strait where the 20 Hz component of their calls can form continuous bands when multiple animals vocalise together^57^. Other marine mammal species also identified in the recordings were narwhals (*Monodon monoceros*), belugas (*Delphinapterus leucas*), delphinids (*Delphinidae*) and sperm whales (*Physeter macrocephalus*). Even during times of peak biological activity (e.g. bearded seal calling in April to June), the clear call patterns observable from the tonal detector outputs were hardly recognisable in the median TOL plots (e.g. see “Supplementary Soundscape Figures” for station 7) and detector outputs generally had little or no temporal correlation with median noise (Figs. 5, 6). Therefore, the increase in noise levels in all three decade bands during late summer and autumn in Melville Bay, Baffin Bay and Greenland Sea is likely not due to seasonal activity in call patterns, which suggests the aforementioned environmental, geophysical and/or anthropogenic noise sources as the more likely drivers of the higher ambient noise. Thus, in this study, although the soundscape contained a rich diversity of biological sounds, TOL measurements were not a good proxy for biological activity, which highlights the need for more targeted detection algorithms for broad-scale identification of call activities.

The large noise variations of tens of decibels here presented between years and locations are consistent with the multi-year TOL observations of Ahonen et al*.* for the same site in Greenland Sea^48^. This underscores that Arctic marine mammals must have adapted to cope with highly varying noise levels in their natural environment. However, whether or not that constitutes an advantage for coping with the added effects of noise pollution depends on the degree to which the animals are already challenged by natural soundscape variation; a question that in our opinion remains to be answered with dedicated studies. In areas where noise levels are naturally high (e.g. near glaciers where iceberg calving occurs), it is unknown to what extent the Arctic marine mammals are disturbed by the high noise levels and impulsive sounds or if they are attracted due to higher primary productivity near marine-terminating glaciers^58^. Also, if important periods, such as breeding seasons, overlap with periods of naturally occurring low ambient noise levels, then modest introduction of human activity may considerably impact communication range for vocalising animals or impede auditory scene analysis. Masking effects on communication range are relatively straightforward to quantify for uniform noise as a range reduction factor directly from the increase in noise level for a given frequency band^59^, however, temporal and spectral noise composition dynamics may complicate evaluation of the masking effects^60^.To fully address impacts on marine life, ambient noise levels and animal behavioural responses should be studied in various time scales to uncover both immediate disturbance responses as well as long-term effects such as habituation, sensitisation and chronic stress^61–63^.

The ability to reliably assess noise impacts on marine wildlife rests on a representative coverage of spatiotemporal variation in ambient noise levels for the area in question. Naturally, more loggers and longer deployments increase data robustness, but also the monitoring costs. Accordingly, monitoring programmes must balance the number of loggers and their deployments in time and space against the likelihood of sufficiently sampling the soundscape in a given area. This study gives an indication on the number of recording stations that are necessary for addressing broad-scale noise levels in time and space in the Arctic. In Melville Bay for example, stations 3 and 4 were spaced 52 km apart and recorded in the same time span (Table 1), and median decade levels differed up to 4 dB (Fig. 2c,d). The stations 1, 2, and 5 were spaced up to 110 km apart and recorded in different years, and their median decade levels differed up to 12 dB (Fig. 2a,b,e). Stations 2 and 3 were located < 0.5 km apart, but deployed in different years, and showed 17 dB difference in median levels of each decade band (Fig. 2b,c). It seems that even for long-term recordings, a small data set of only two or three deployments could face a high risk of inadequately sampling the noise variation in a given environment, and that separation of the acoustic loggers in time as well as space is important when monitoring noise levels. This study shows that in some cases the noise levels recorded in one year may have little predictive power for noise levels at the same site in later years. In contrast, a large-scale long-term noise study in the Baltic found very similar noise levels between 2 years at two sites^27^, highlighting that Arctic underwater noise variation might be substantial in comparison to temperate regions, which likely relates to interannual differences in sea ice cover. The extent of large spatiotemporal noise inconsistencies should be investigated more closely in future studies, as a lack of extrapolative power must be accounted for in noise models and impact assessments.

In conclusion, we have presented PAM data for 26 long-term deployments around Greenland to provide noise baselines and explore broad-scale soundscape trends. Noise levels in the Arctic are shown to be highly variable, highlighting that Arctic marine animals must cope with a wide range of masking and noise exposure levels. Whether Arctic species are evolutionarily adapted to cope better with varying noise levels compared to non-Arctic species is unknown. It remains to be understood if impact can be assessed by noise load only or if the nature and behaviour of noise sources, in terms of rate of change of noise load and spectral composition, is the driver as per the risk-disturbance hypothesis^64^. If so, then anthropogenic noise pollution in the Arctic may increase the risk of severely challenging marine mammals that are already dealing with natural noise variation in their environment. In line with previous studies^46,51^, we show that ice cover can act to decrease ambient noise to low levels during winter and spring, but ice can also produce a variety of transient and tonal sounds and increase noise levels considerably. Seismic airguns were another commonly identified sound source that could dominate soundscapes over extended durations. Further studies are needed to quantify, classify, and disentangle the noise contributions stemming from geophysical, biological, and anthropogenic sound sources in the Artic that is facing rapid reductions in ice cover and consequentially increases in anthropogenic activities and noise.

## Supplementary Information

Supplementary Information.

## Data Availability

The datasets generated during and/or analysed during the current study are available from the corresponding author on reasonable request.
