# Author Correction: Soundscape and ambient noise levels of the Arctic waters around Greenland

**DOI:** 10.1038/s41598-021-03640-x

**Published:** 2021-12-14

**Authors:** Michael Ladegaard, Jamie Macaulay, Malene Simon, Kristin L. Laidre, Aleksandrina Mitseva, Simone Videsen, Michael Bjerre Pedersen, Jakob Tougaard, Peter Teglberg Madsen

**Affiliations:** 1 Zoophysiology, Department of Biology, Aarhus University, Aarhus, Denmark; 2 Greenland Institute of Natural Resources, Nuuk, Greenland; 3 Greenland Climate Research Centre, Greenland Institute of Natural Resources, Nuuk, Greenland; 4 Polar Science Center, Applied Physics Laboratory, University of Washington, Seattle, WA USA; 5 Marine Mammal Research, Department of Ecoscience, Aarhus University, Roskilde, Denmark

Correction to: *Scientific Reports* 10.1038/s41598-021-02255-6, published online 03 December 2021

The original version of this Article contained an error in the spelling of the author Jamie Macaulay which was incorrectly given as Jamie Macauley. The original Article has been corrected.

