# Supplementary material for: Soundscape and ambient noise levels of the Arctic waters around Greenland
Source: Sci Rep. 2021 Dec 3;11:23360. doi: 10.1038/s41598-021-02255-6 (PMC8642542; doi:10.1038/s41598-021-02255-6)
Supplement: Supplementary file 1 — Supplementary Information. [file 41598_2021_2255_MOESM1_ESM.zip › Ladegaard_etal_Suppl_Info_ESM.zip!/Ladegaard_etal_Suppl_Info/Suppl Figure S1 TOL_63_125_2000Hz_all_stations.pdf]

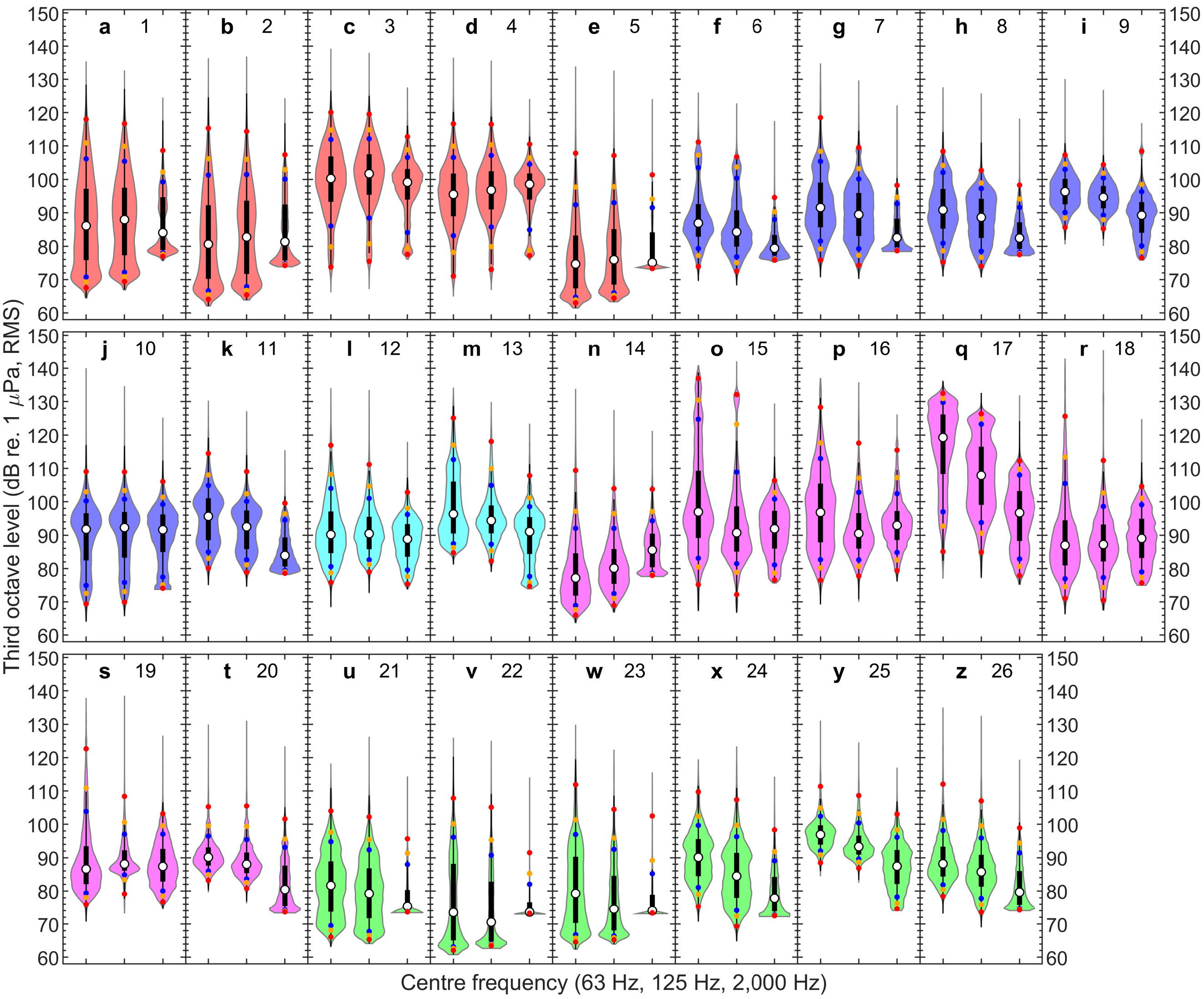

Third octave level (TOL) distributions in the 63, 125, and 2,000 Hz third octave bands for the 26 recording stations. **a-z** The TOL distributions are depicted as violin plots overlaid with box plots (interquartile range: thick black lines; whiskers: thin black lines), as the decade levels distributions in Fig. 2. Exceedance levels are shown as coloured dots (L50 or median: white; L90 and L10: blue; L95 and L5: orange; L99 and L1: red). Note that in Fig. 2, the 1-10 kHz decade level was not computed for all stations due to low sampling rates, whereas the 2 kHz TOL could be computed for all stations. Region colours as in Fig. 1 and Table 1 (Melville Bay: red, Baffin Bay: blue, South Greenland: cyan, Tasiilaq: magenta, Greenland Sea: green).
