# Supplementary material for: Soundscape and ambient noise levels of the Arctic waters around Greenland
Source: Sci Rep. 2021 Dec 3;11:23360. doi: 10.1038/s41598-021-02255-6 (PMC8642542; doi:10.1038/s41598-021-02255-6)
Supplement: Supplementary file 1 — Supplementary Information. [file 41598_2021_2255_MOESM1_ESM.zip › Ladegaard_etal_Suppl_Info_ESM.zip!/Ladegaard_etal_Suppl_Info/Suppl Soundscape Figures.pdf]

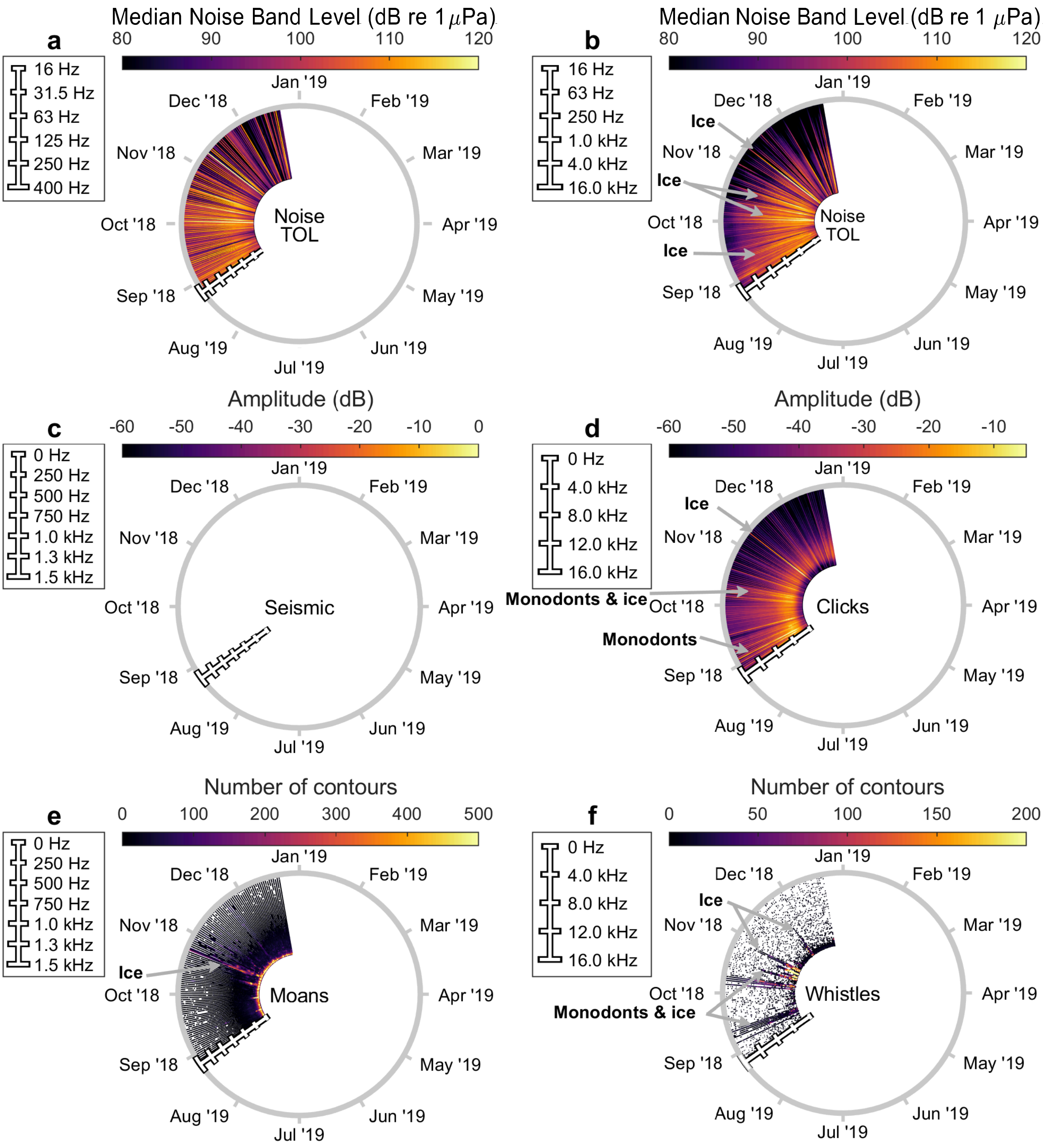

Soundscape figure for station 1. See Fig. 4 for figure explanation. See Table 1 for sampling rate.

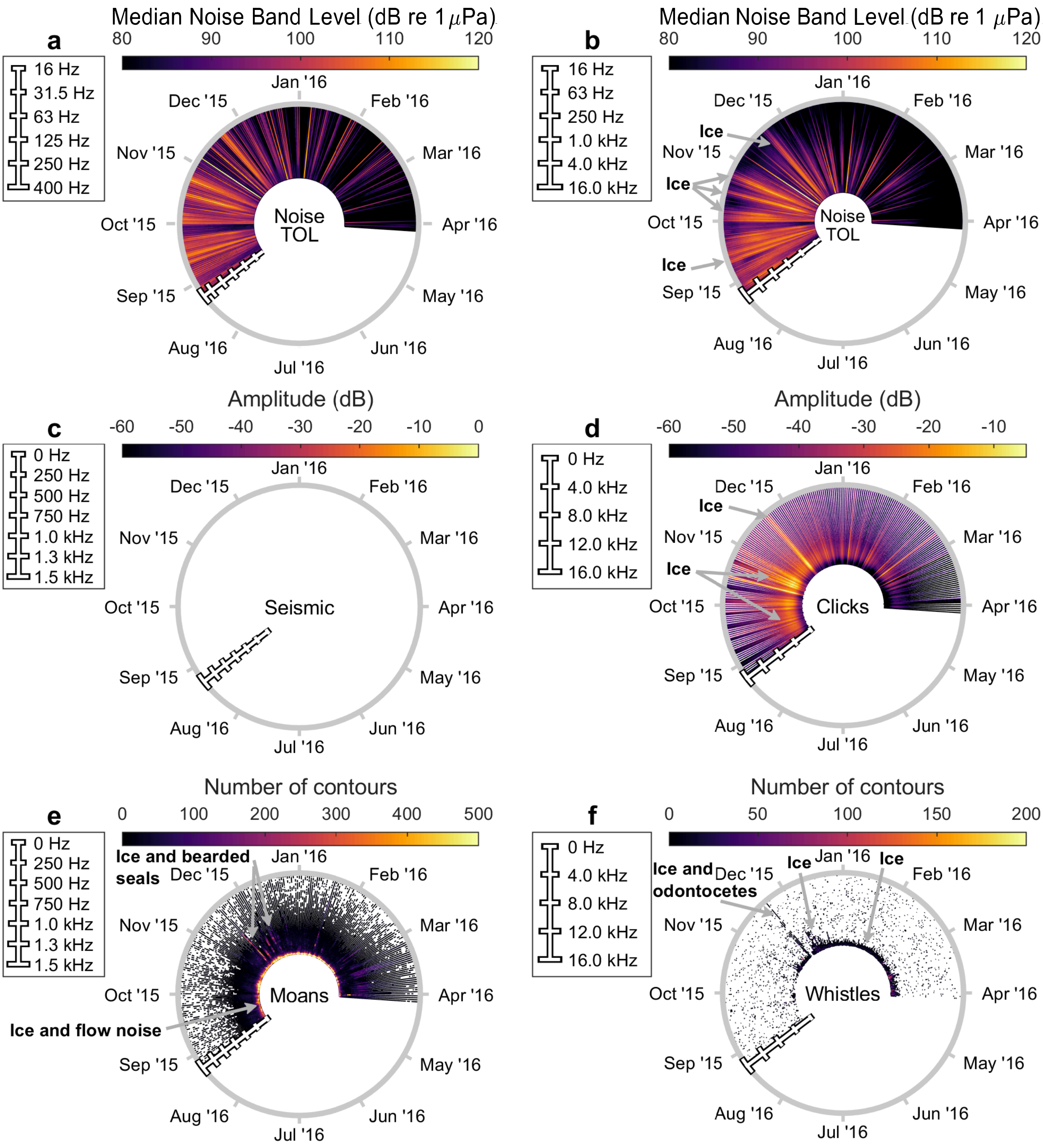

Soundscape figure for station 2. See Fig. 4 for figure explanation. See Table 1 for sampling rate.

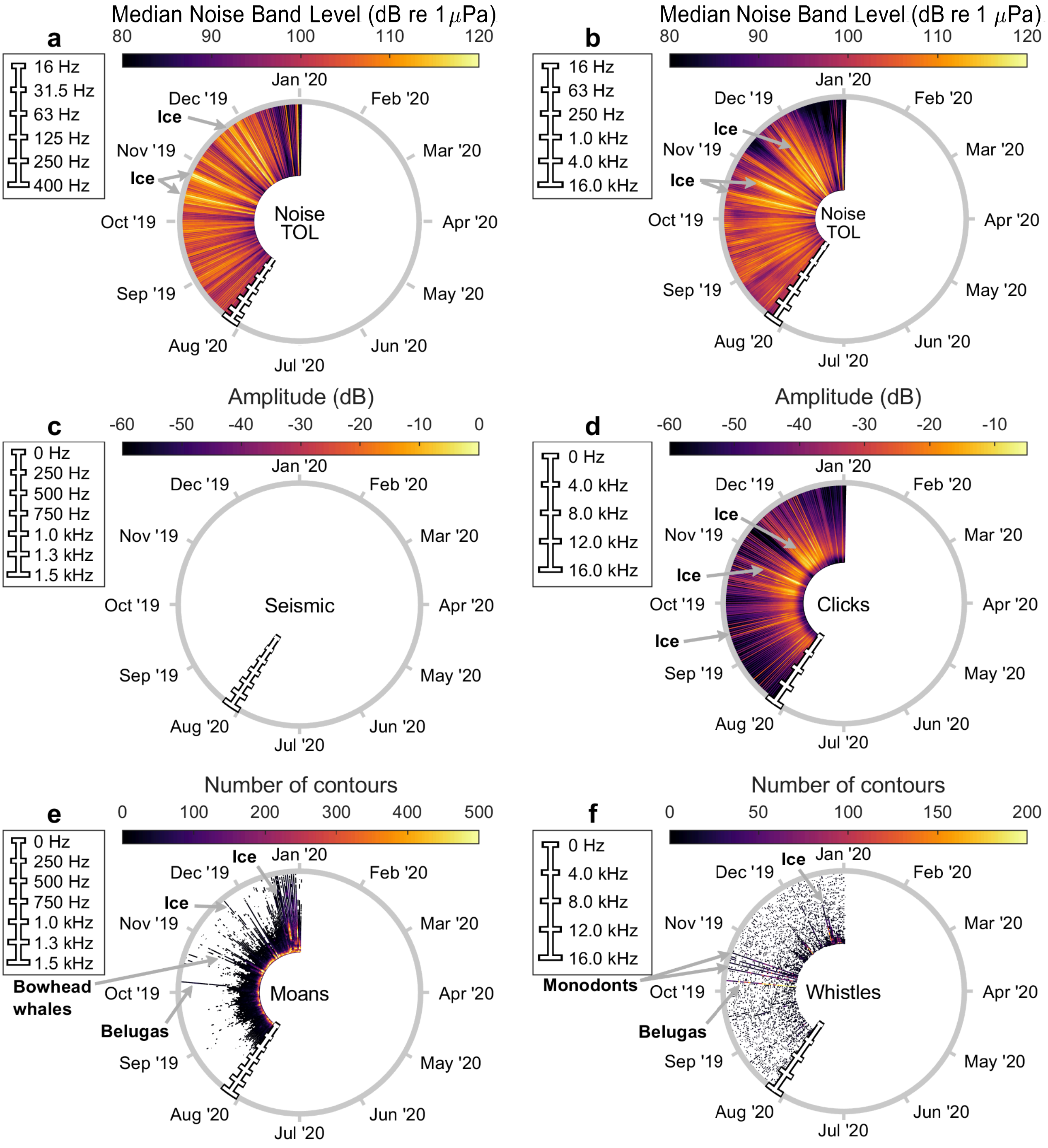

Soundscape figure for station 3. See Fig. 4 for figure explanation. See Table 1 for sampling rate.

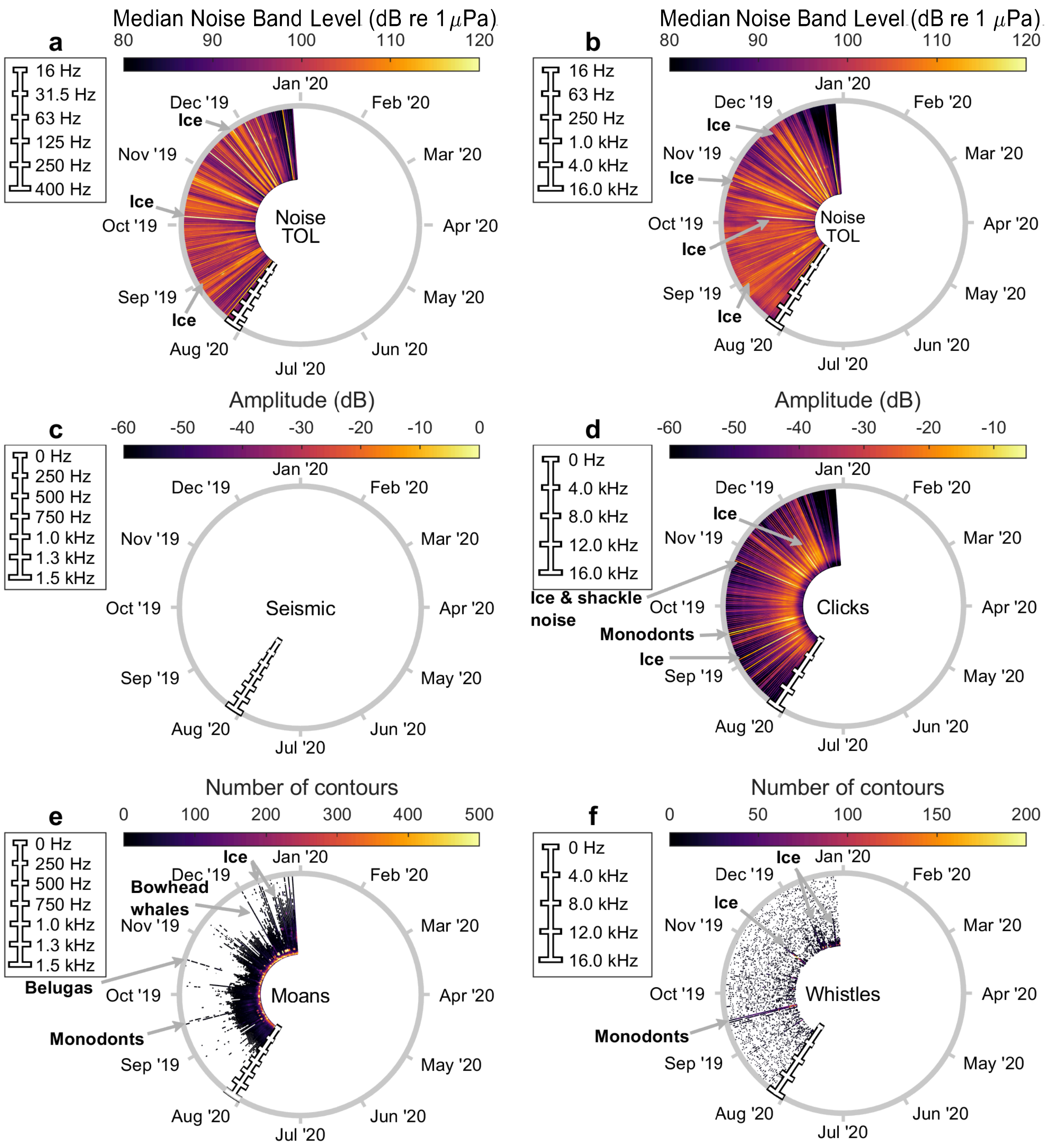

Soundscape figure for station 4. See Fig. 4 for figure explanation. See Table 1 for sampling rate.

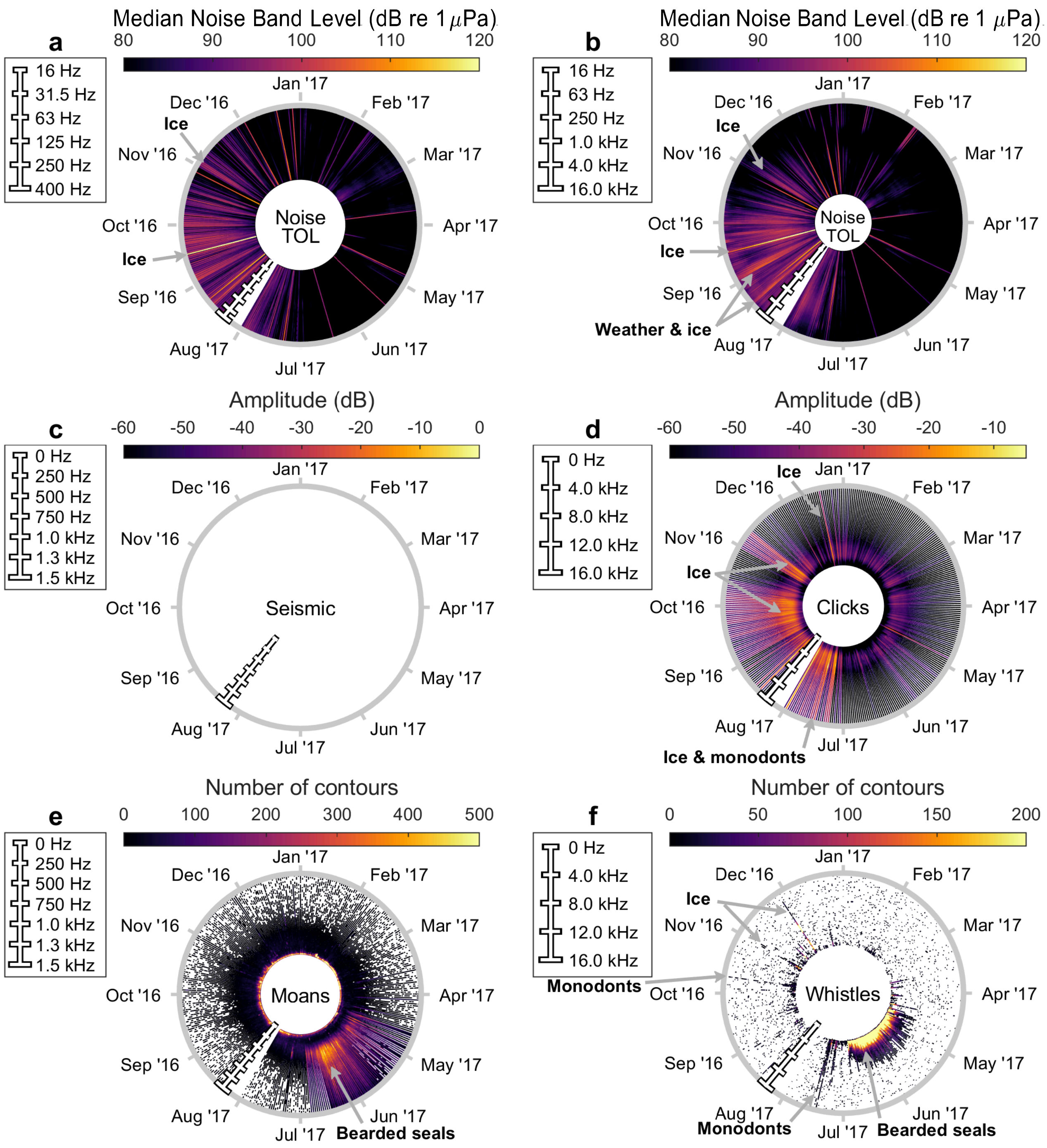

Soundscape figure for station 5. See Fig. 4 for figure explanation. See Table 1 for sampling rate.

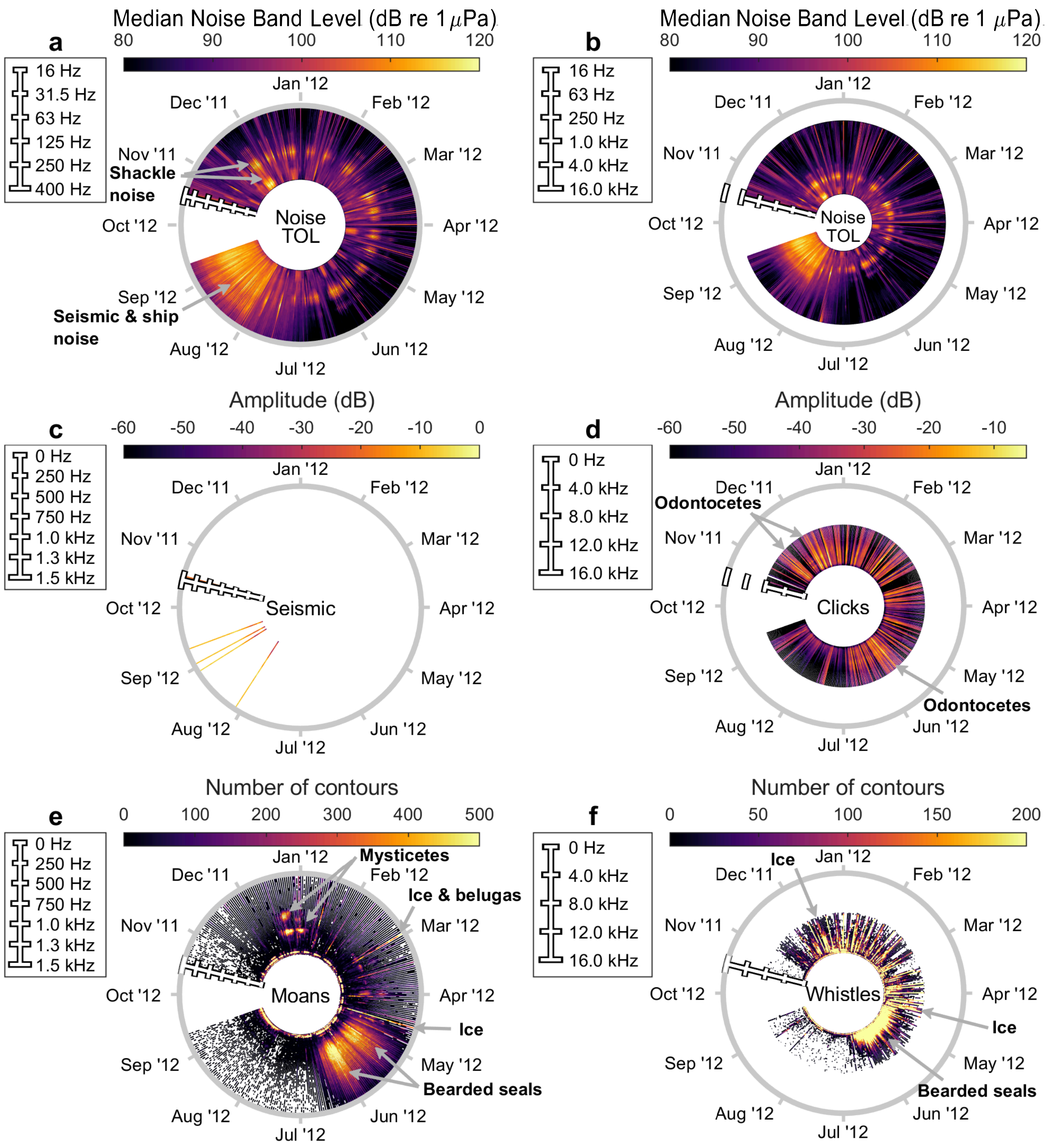

Soundscape figure for station 6. See Fig. 4 for figure explanation. See Table 1 for sampling rate.

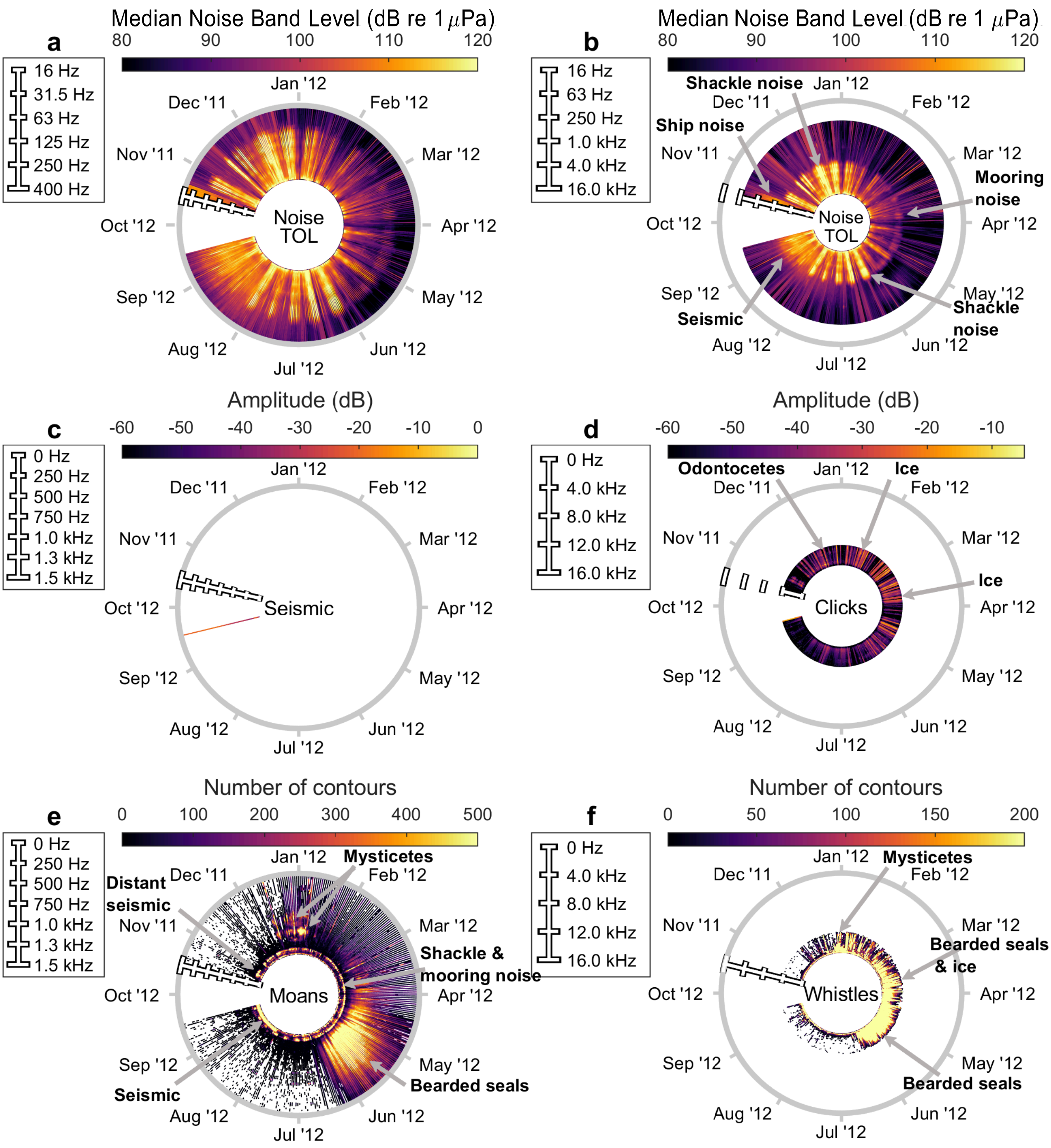

Soundscape figure for station 7. See Fig. 4 for figure explanation. See Table 1 for sampling rate.

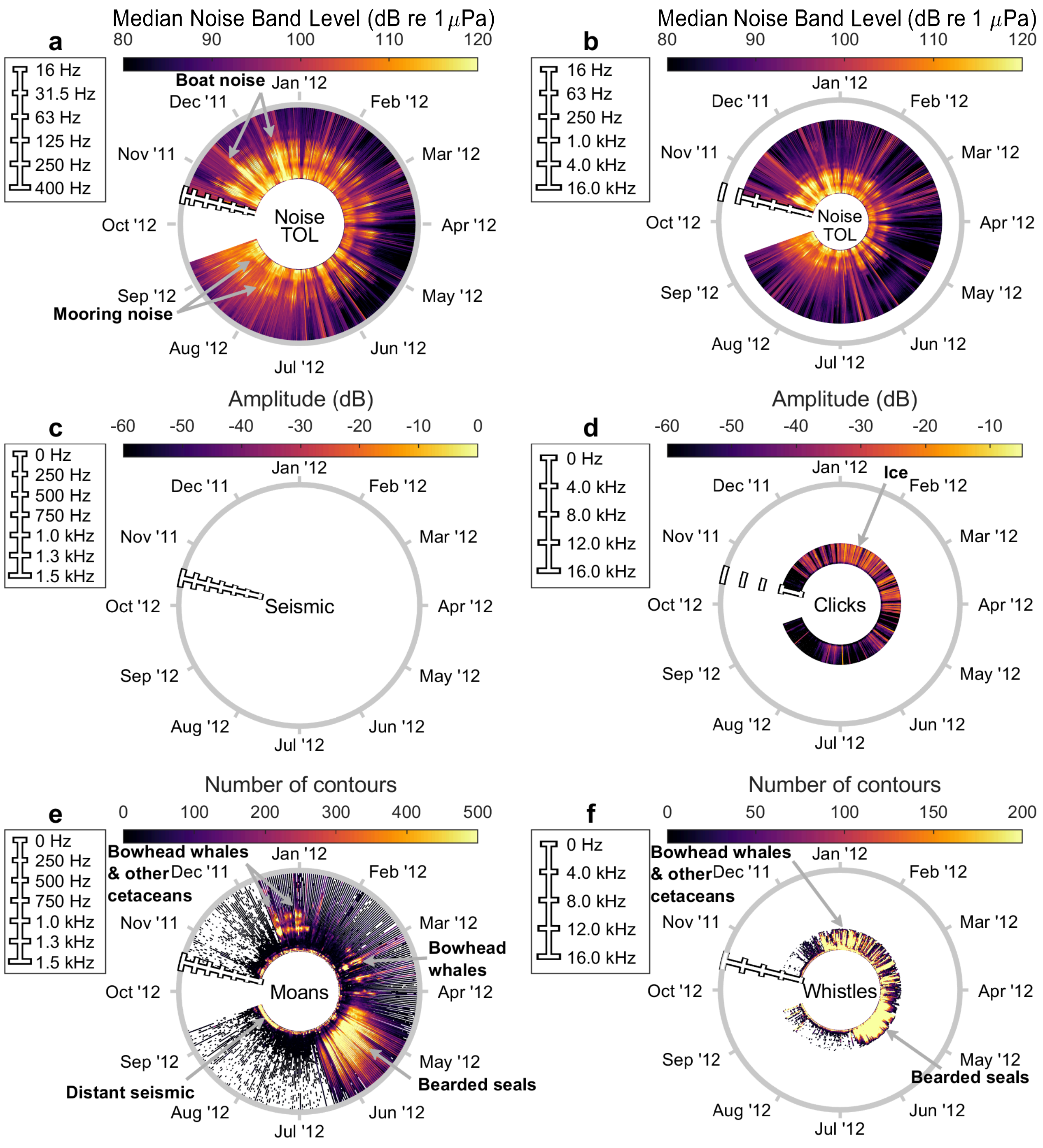

Soundscape figure for station 8. See Fig. 4 for figure explanation. See Table 1 for sampling rate.

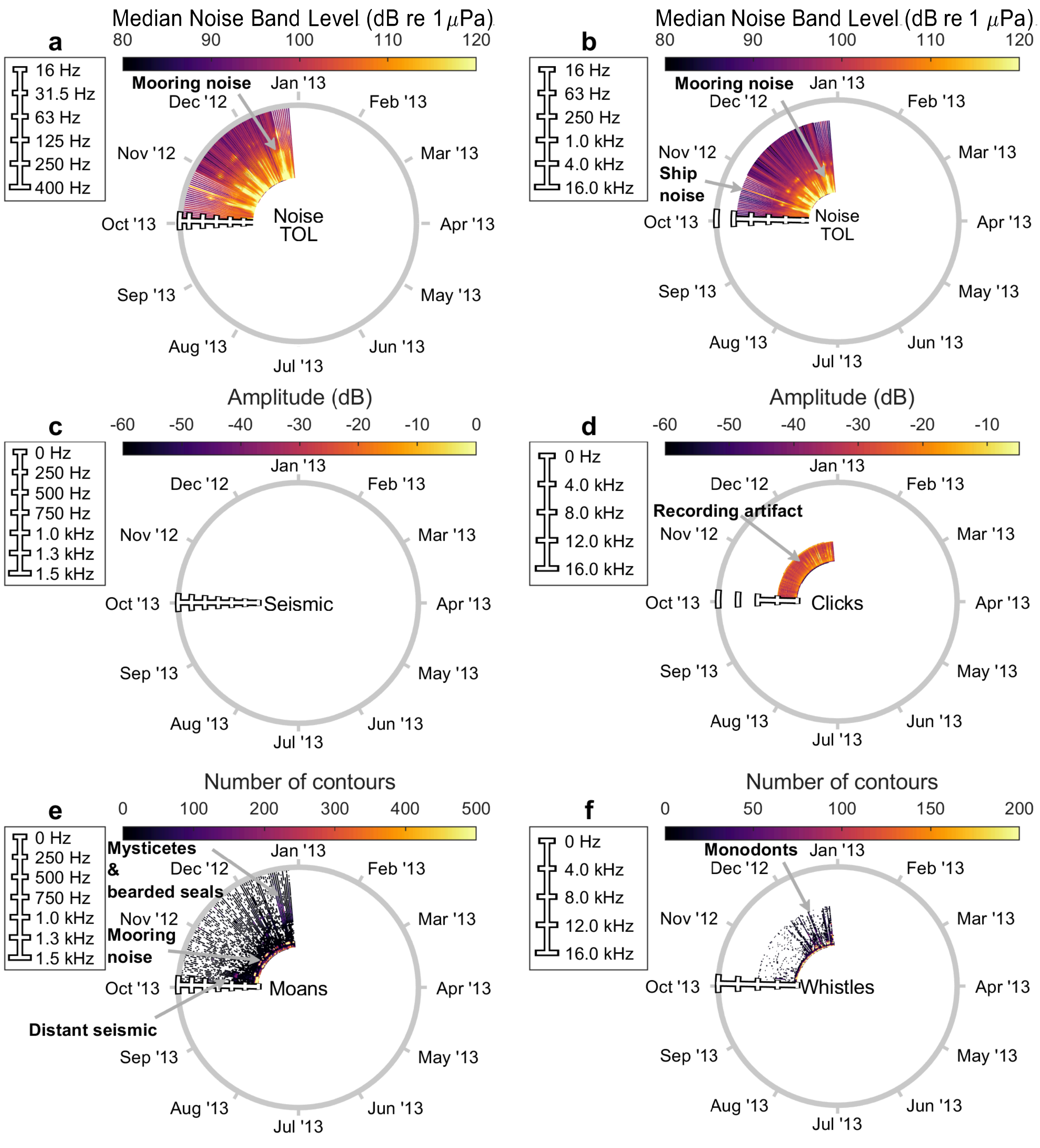

Soundscape figure for station 9. See Fig. 4 for figure explanation. See Table 1 for sampling rate.

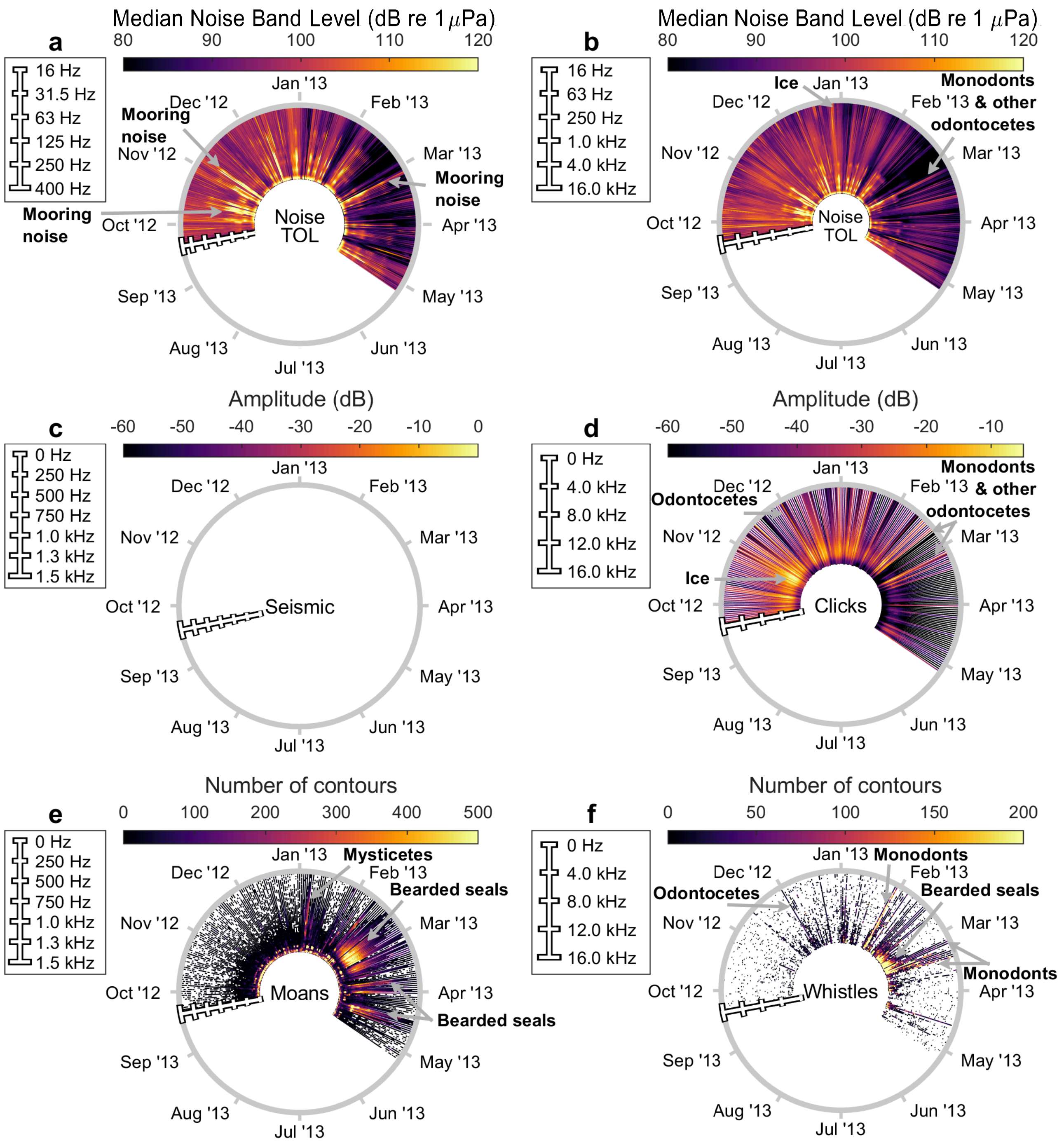

Soundscape figure for station 10. See Fig. 4 for figure explanation. See Table 1 for sampling rate.

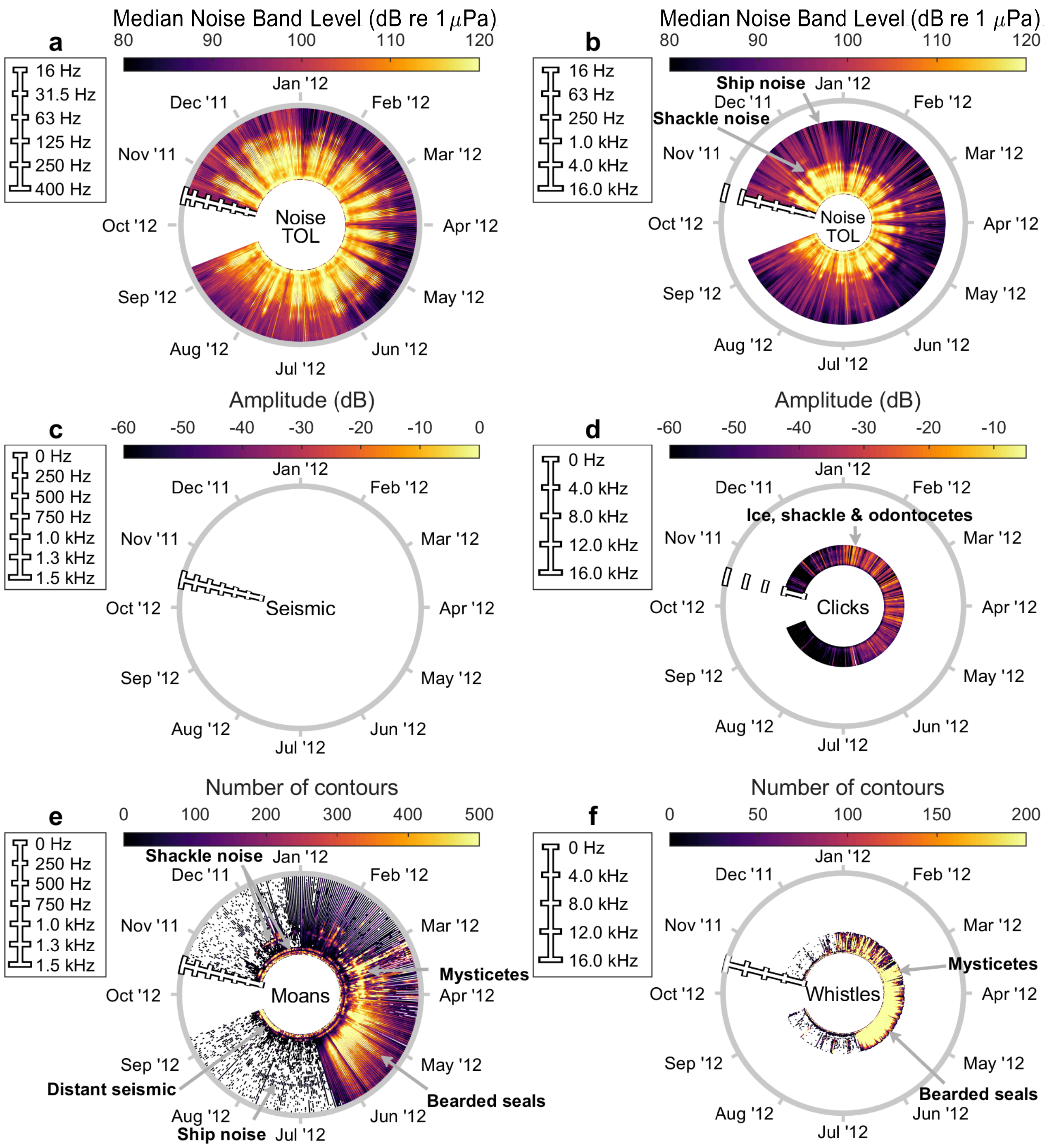

Soundscape figure for station 11. See Fig. 4 for figure explanation. See Table 1 for sampling rate.

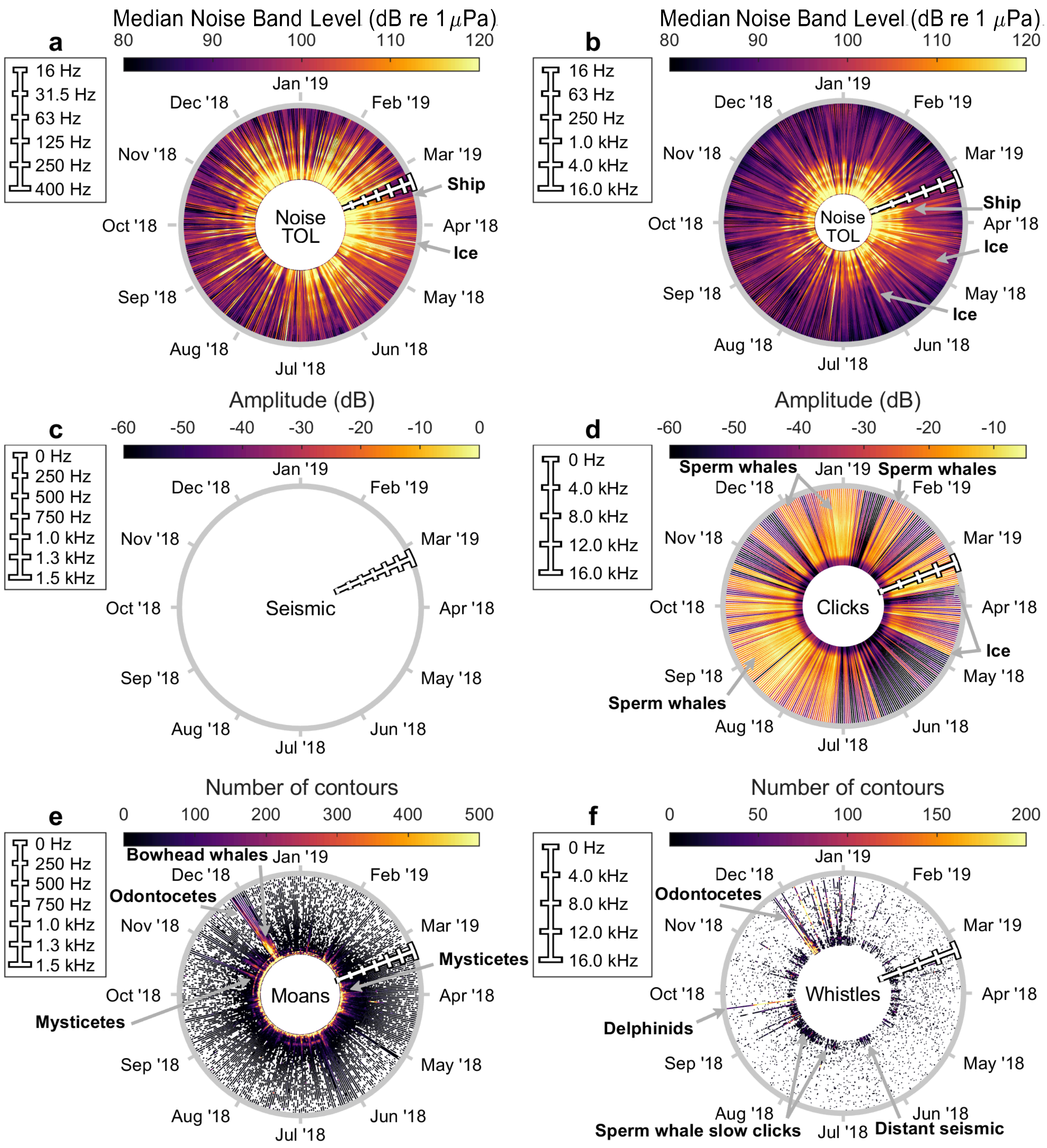

Soundscape figure for station 12. See Fig. 4 for figure explanation. See Table 1 for sampling rate.

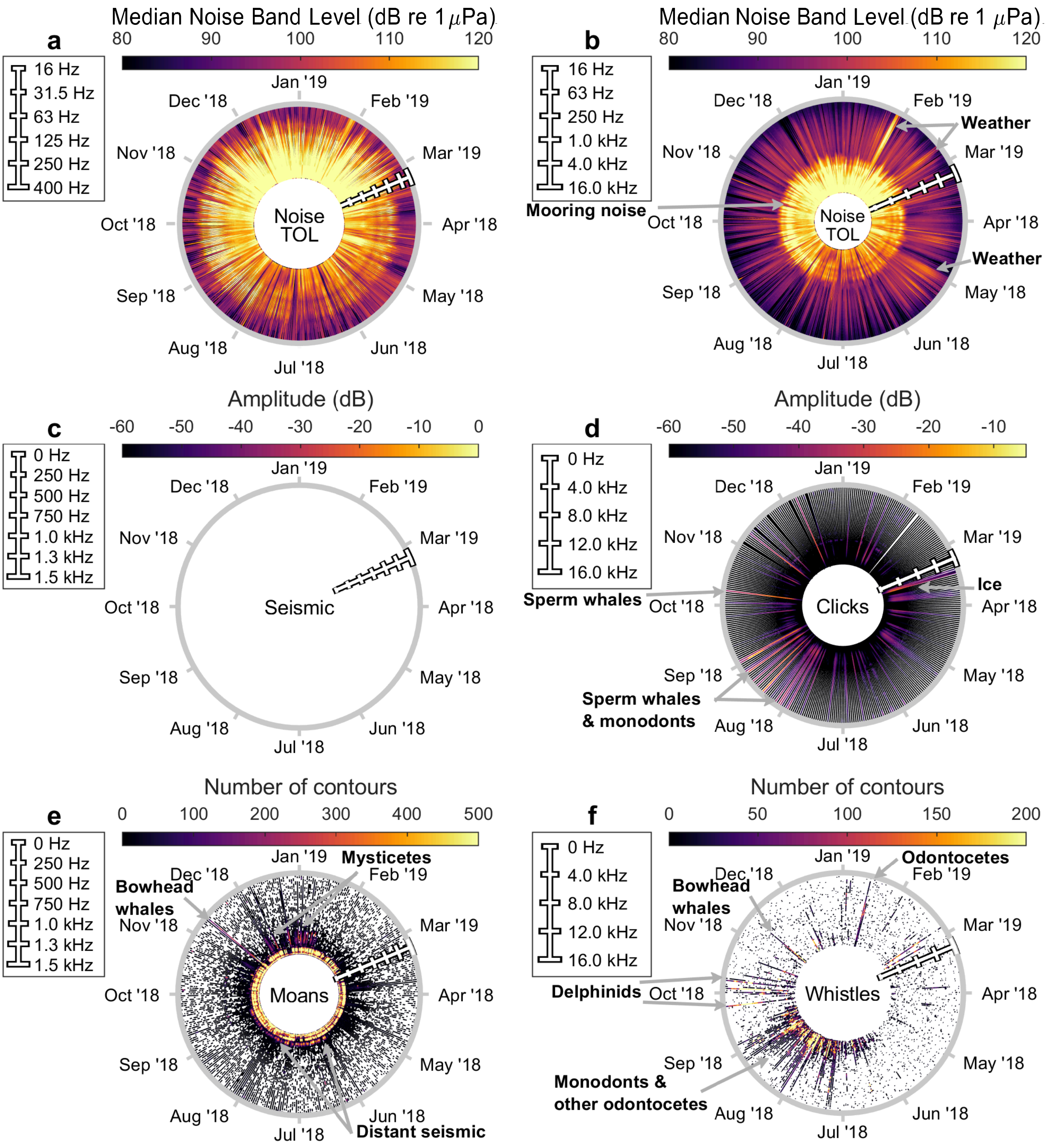

Soundscape figure for station 13. See Fig. 4 for figure explanation. See Table 1 for sampling rate.

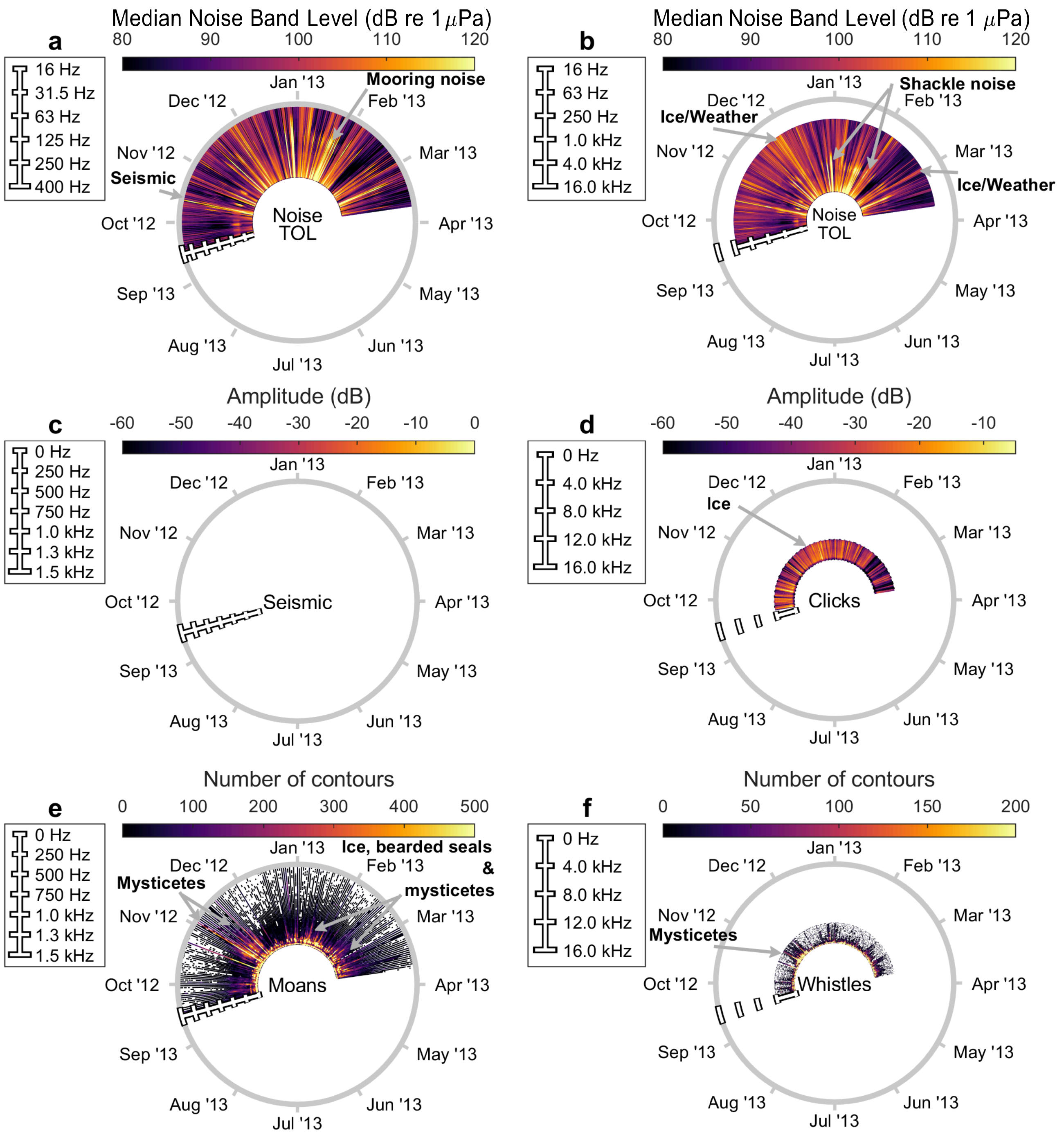

Soundscape figure for station 14. See Fig. 4 for figure explanation. See Table 1 for sampling rate.

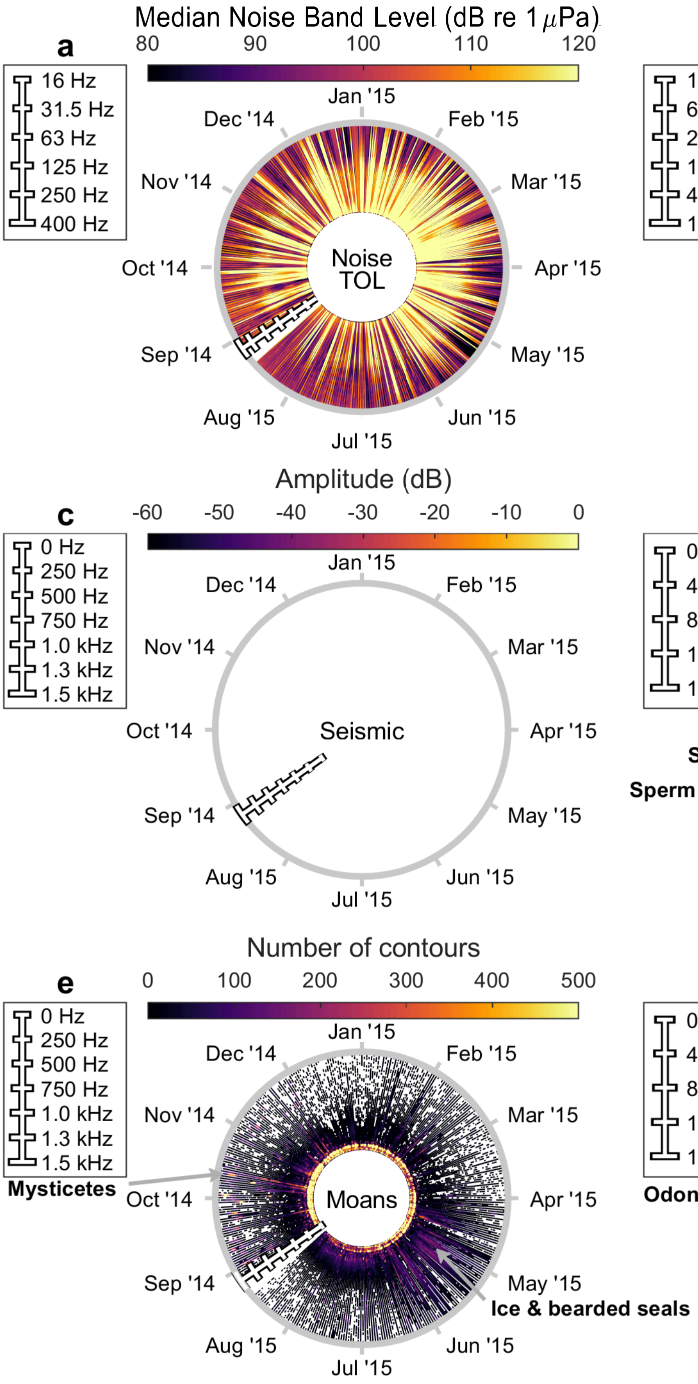

Soundscape figure for station 15. See Fig. 4 for figure explanation. See Table 1 for sampling rate.

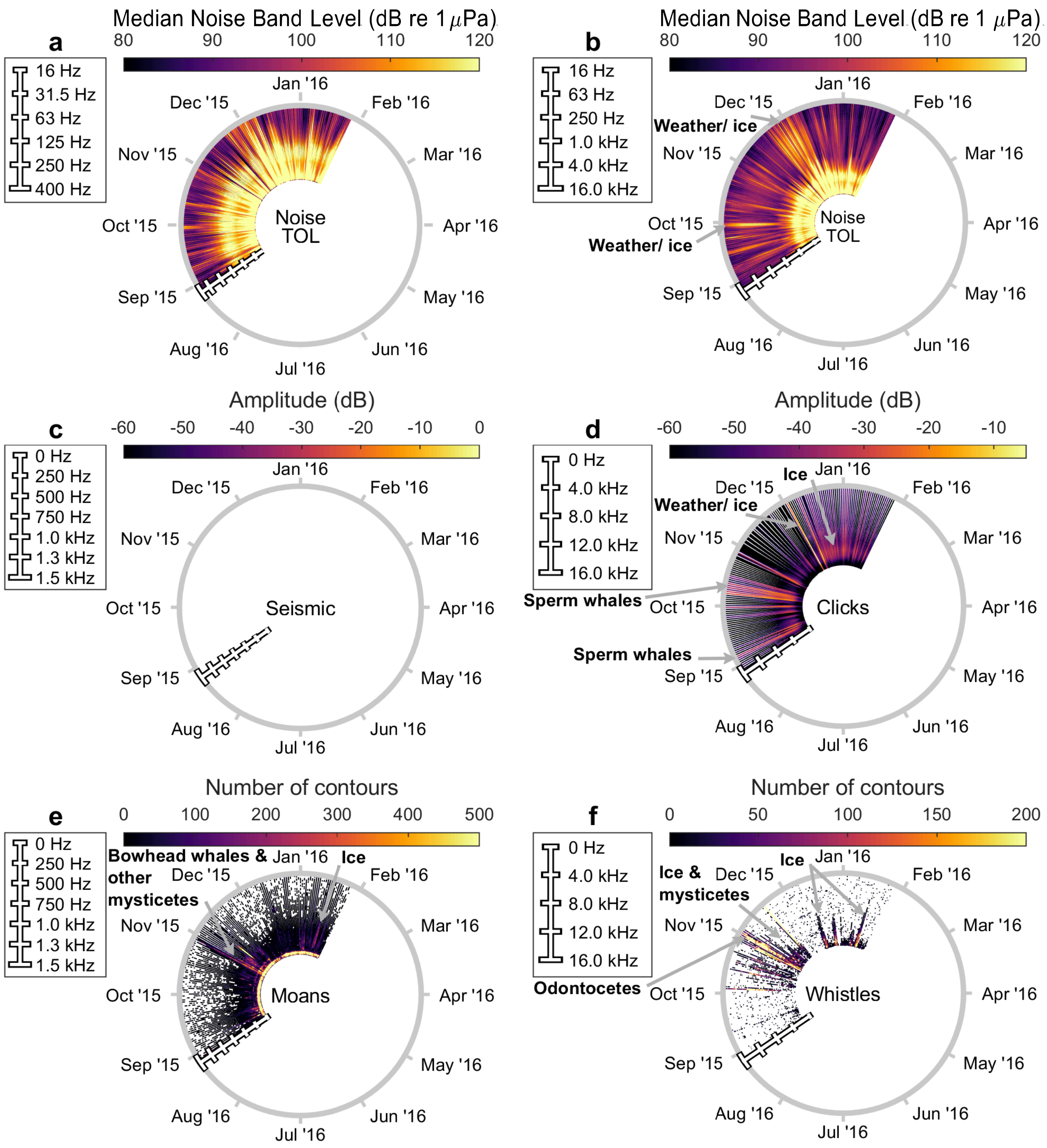

Soundscape figure for station 16. See Fig. 4 for figure explanation. See Table 1 for sampling rate.

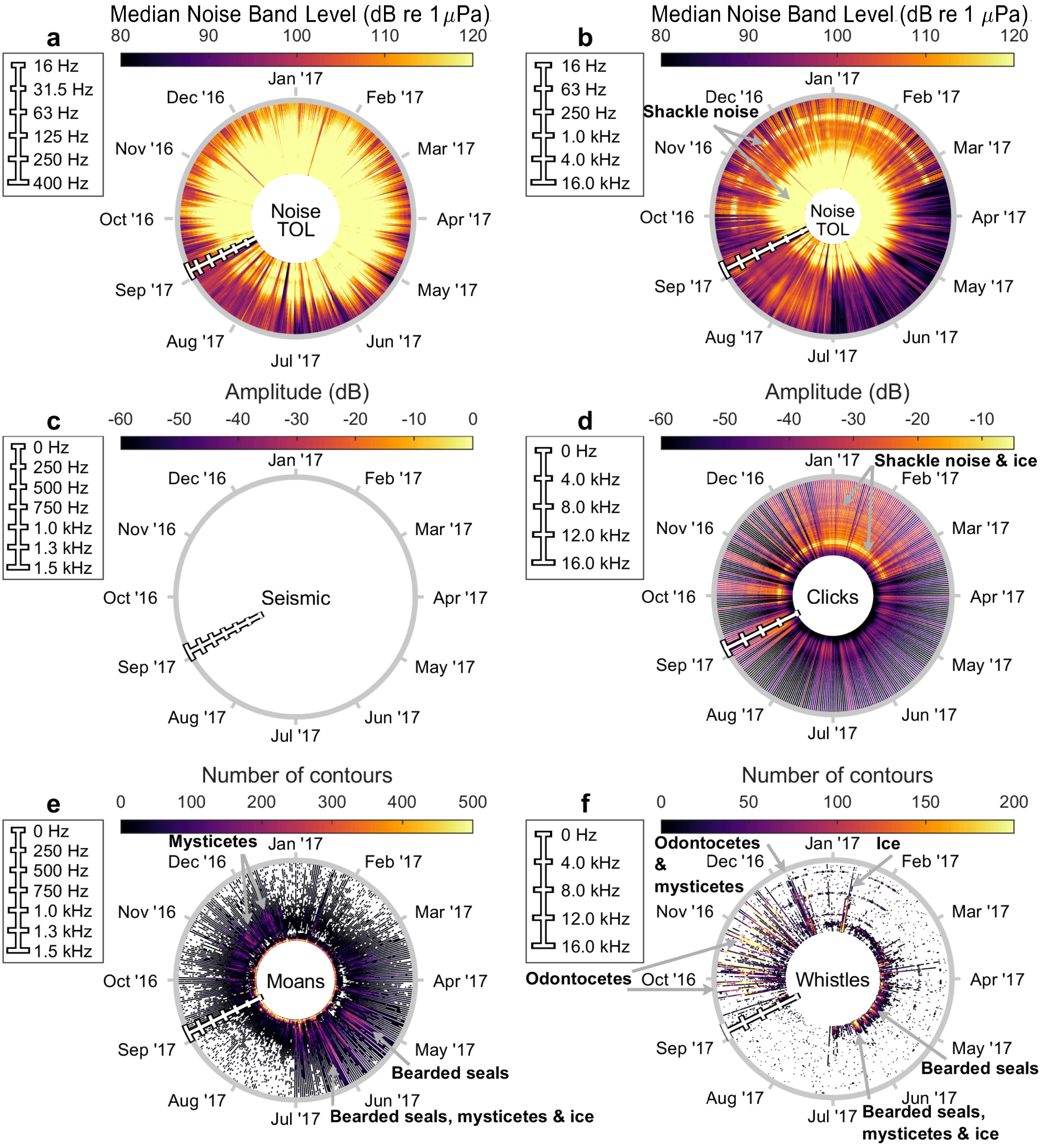

Soundscape figure for station 17. See Fig. 4 for figure explanation. See Table 1 for sampling rate.

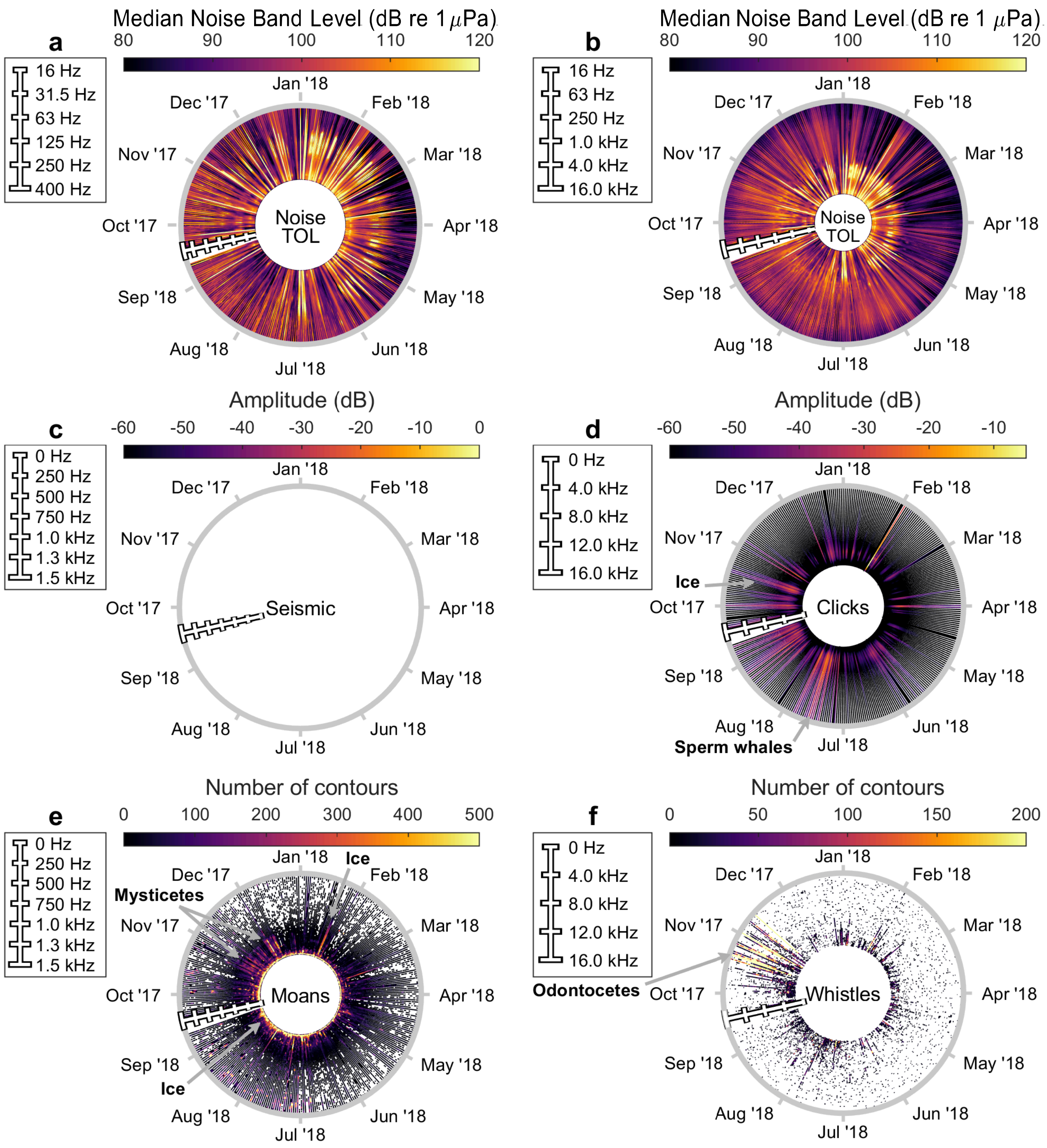

Soundscape figure for station 18. See Fig. 4 for figure explanation. See Table 1 for sampling rate.

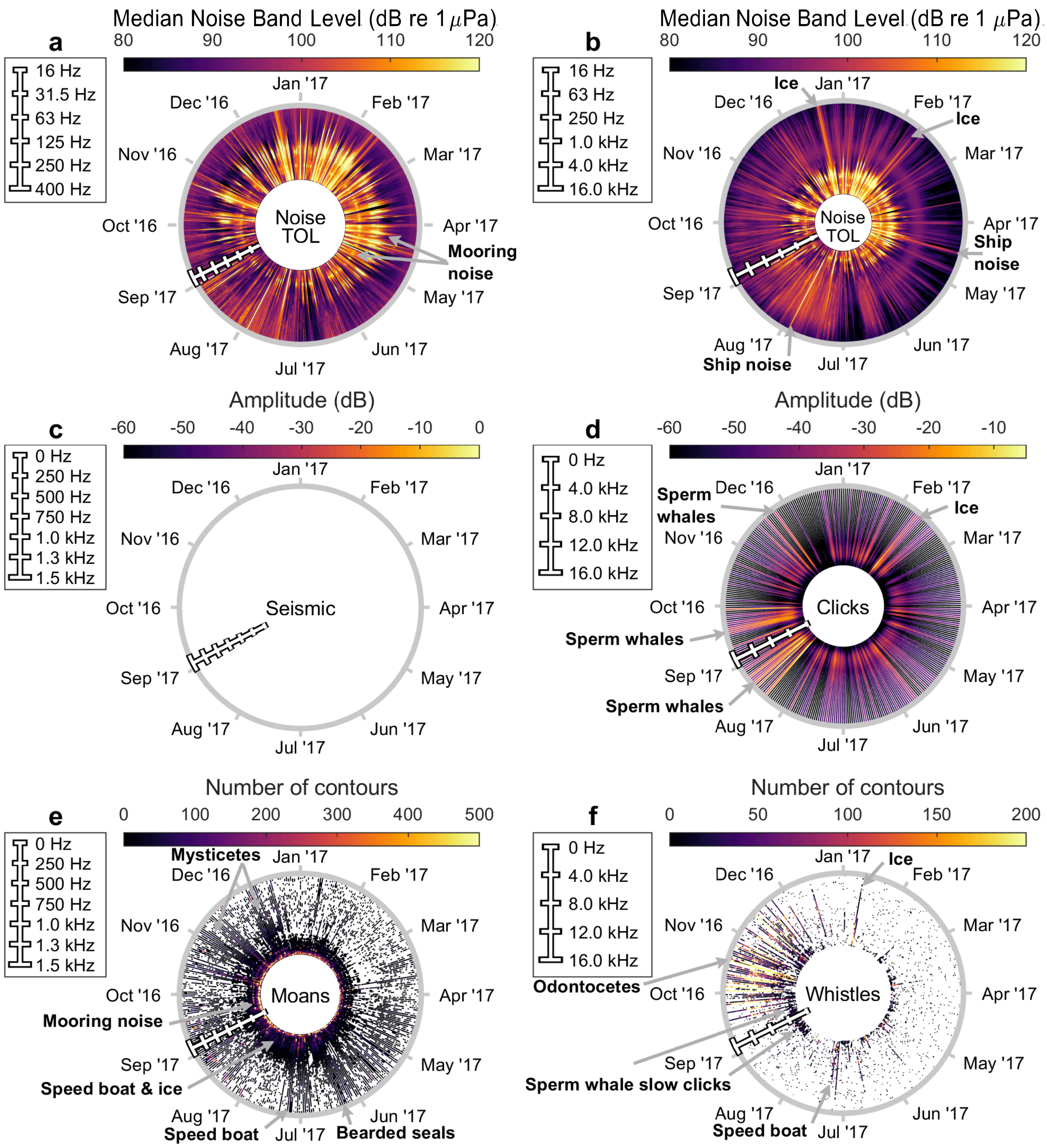

Soundscape figure for station 19. See Fig. 4 for figure explanation. See Table 1 for sampling rate.

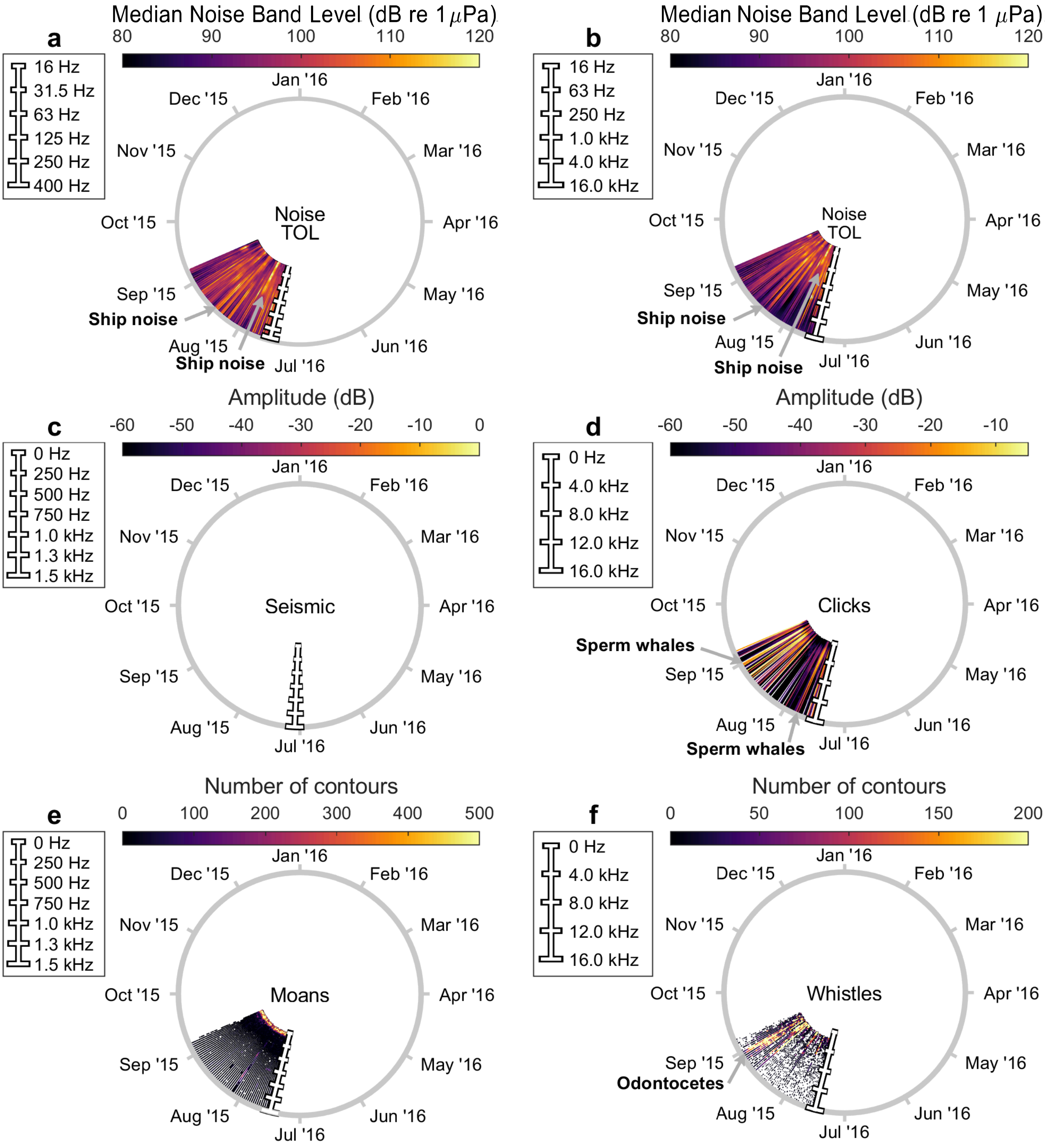

Soundscape figure for station 20. See Fig. 4 for figure explanation. See Table 1 for sampling rate.

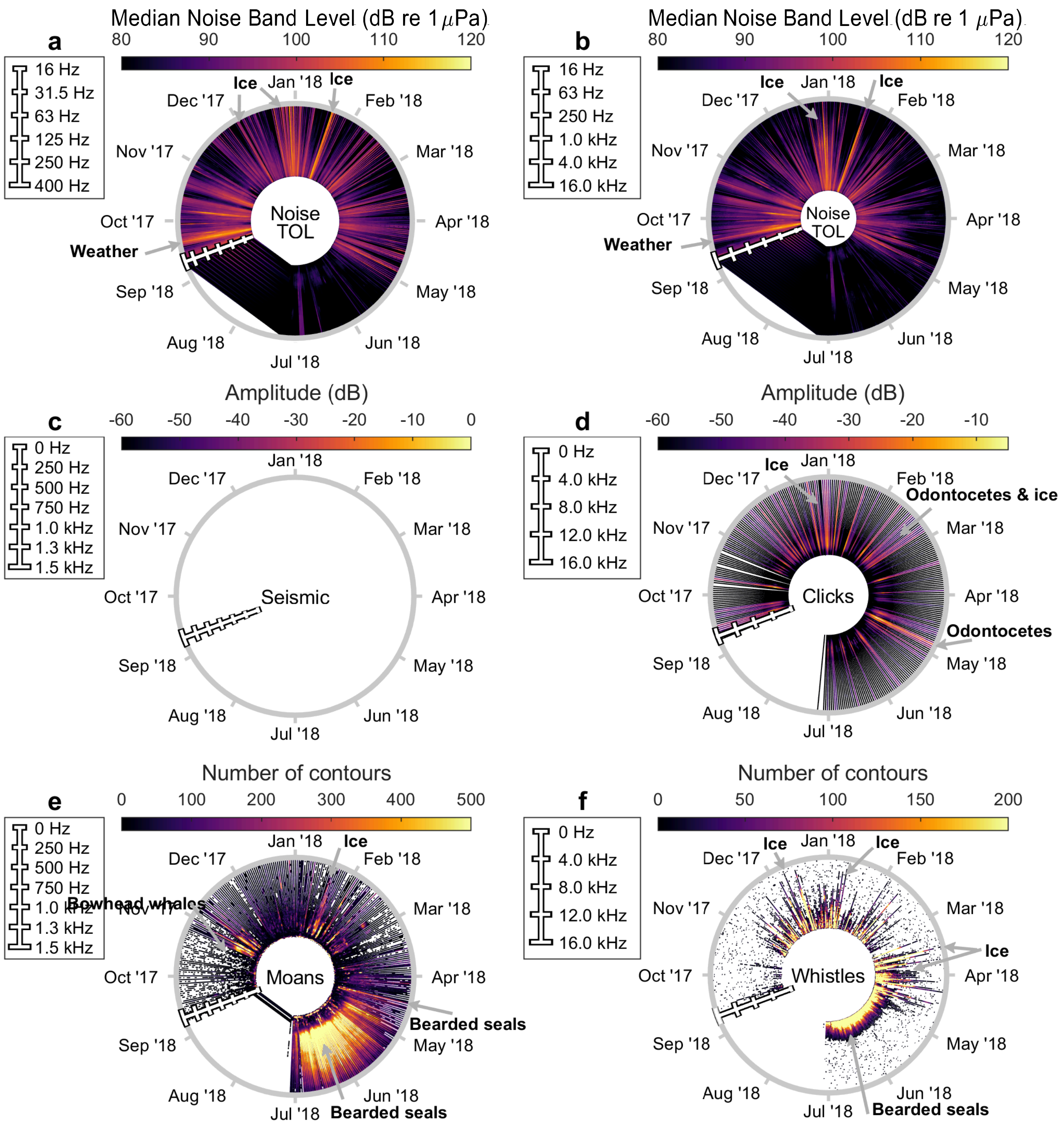

Soundscape figure for station 21. See Fig. 4 for figure explanation. See Table 1 for sampling rate.

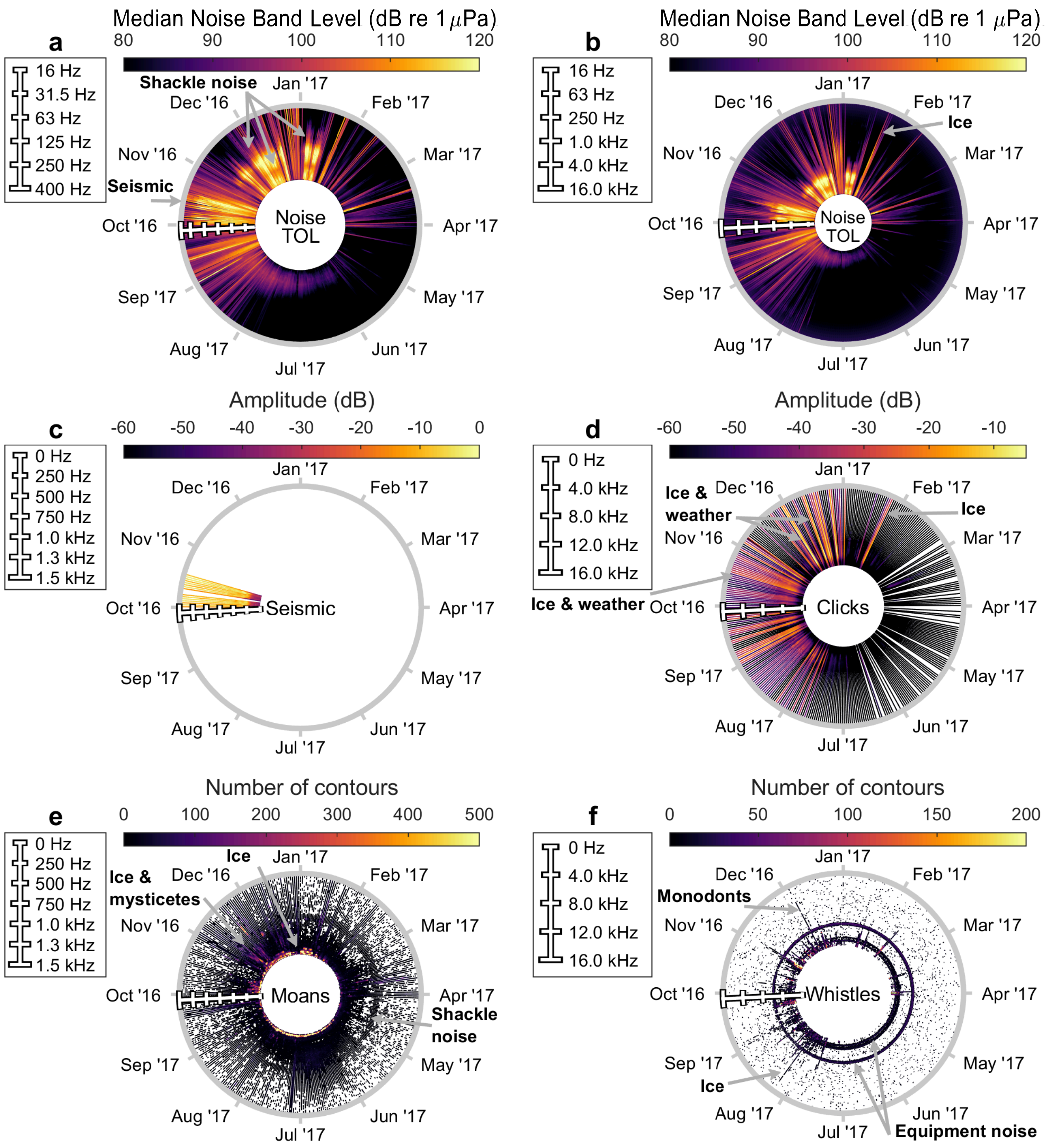

Soundscape figure for station 22. See Fig. 4 for figure explanation. See Table 1 for sampling rate.

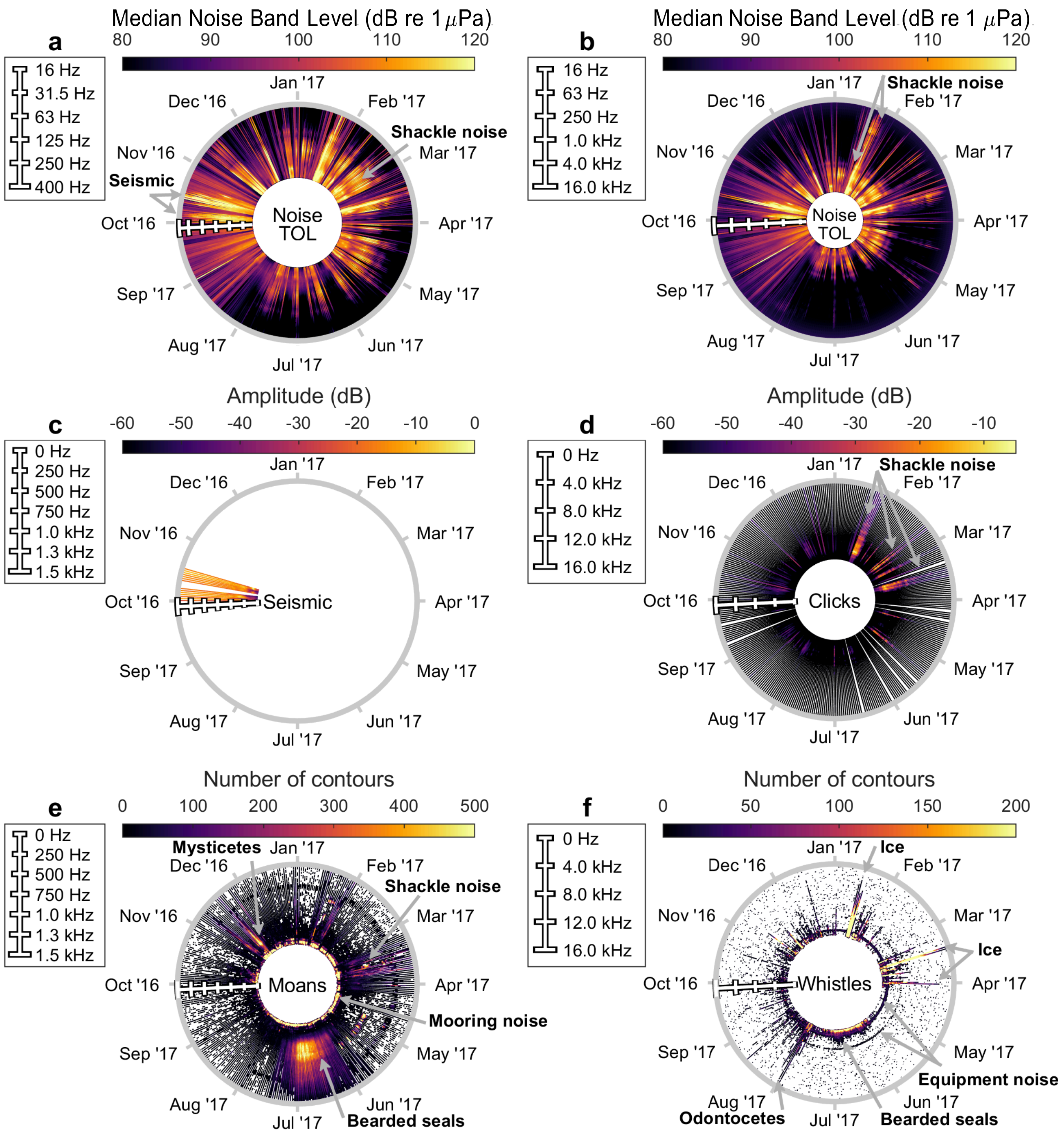

Soundscape figure for station 23. See Fig. 4 for figure explanation. See Table 1 for sampling rate.

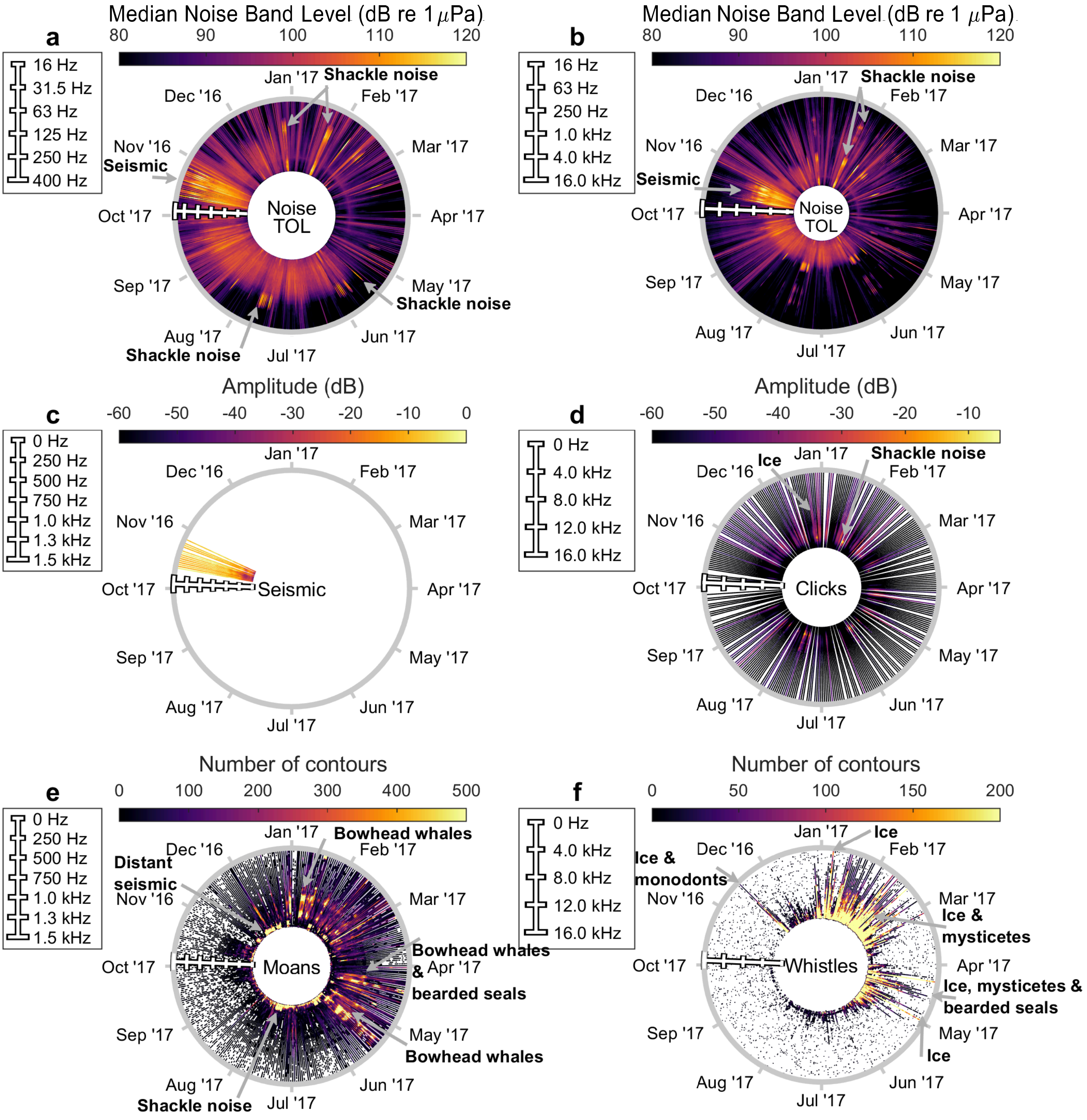

Soundscape figure for station 24. See Fig. 4 for figure explanation. See Table 1 for sampling rate.

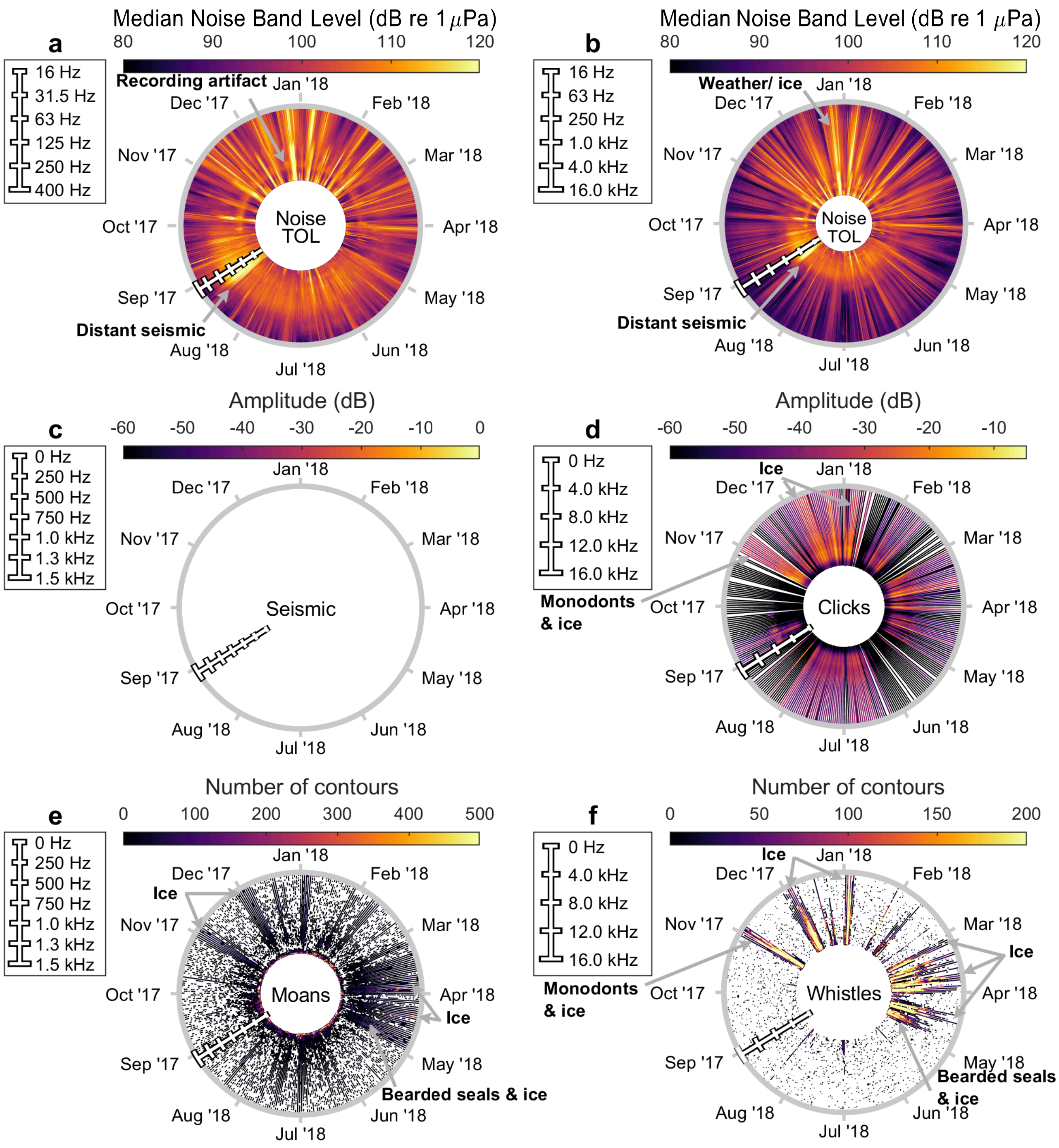

Soundscape figure for station 25. See Fig. 4 for figure explanation. See Table 1 for sampling rate.

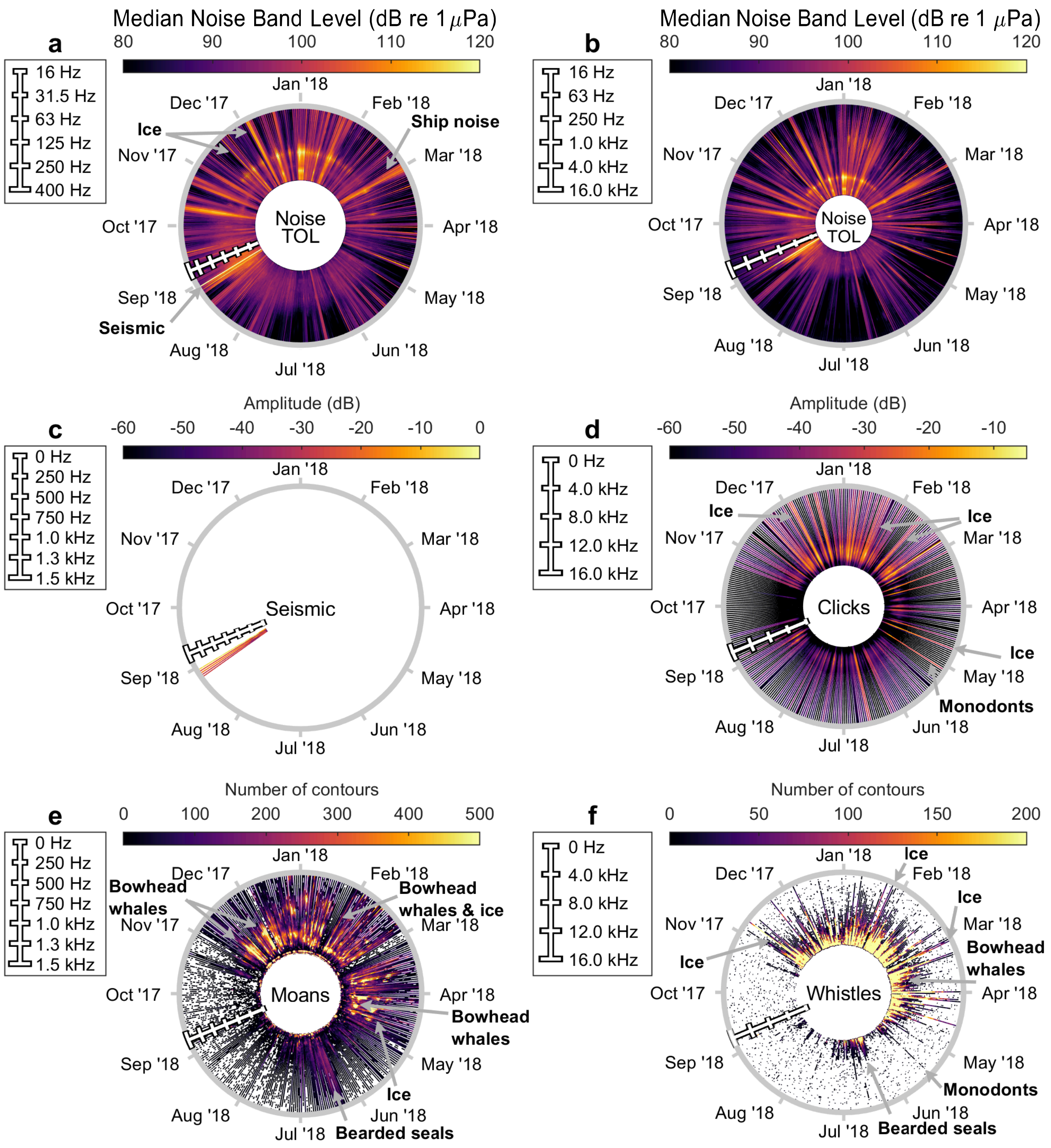

Soundscape figure for station 26. See Fig. 4 for figure explanation. See Table 1 for sampling rate.
