# Supplementary material for: Soundscape and ambient noise levels of the Arctic waters around Greenland
Source: Sci Rep. 2021 Dec 3;11:23360. doi: 10.1038/s41598-021-02255-6 (PMC8642542; doi:10.1038/s41598-021-02255-6)
Supplement: Supplementary file 1 — Supplementary Information. [file 41598_2021_2255_MOESM1_ESM.zip › Ladegaard_etal_Suppl_Info_ESM.zip!/Ladegaard_etal_Suppl_Info/Suppl TOL Figures.pdf]

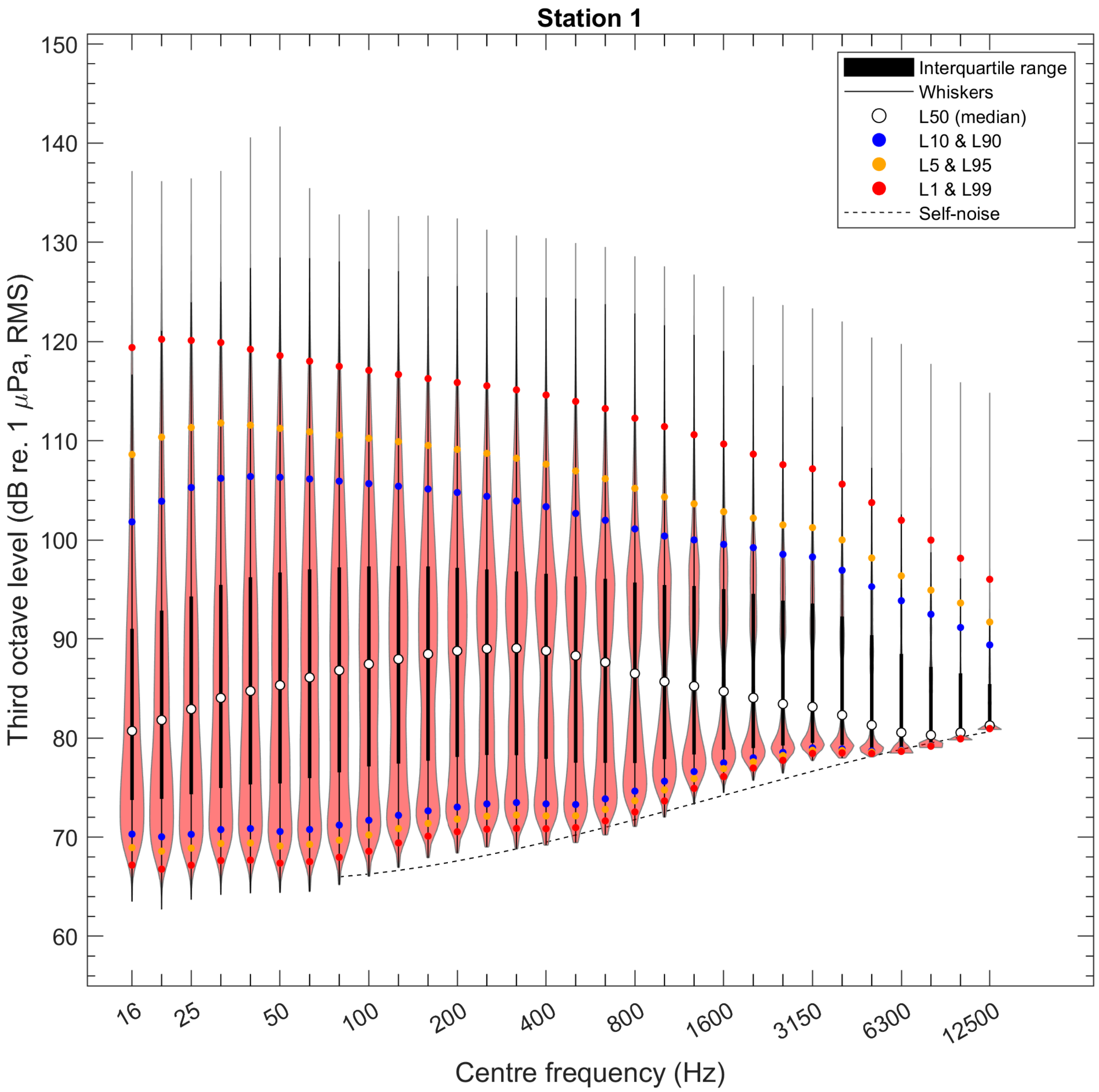

Third octave levels (TOLs) at station 1. The TOL distribution at each third octave band is represented with a violin plot overlaid with a boxplot and points at different exceedance levels (see legend). Boxplot whiskers extend to the lowest and highest data points observed within a distance of 1.5 times the interquartile range from the first and third quartiles, respectively.

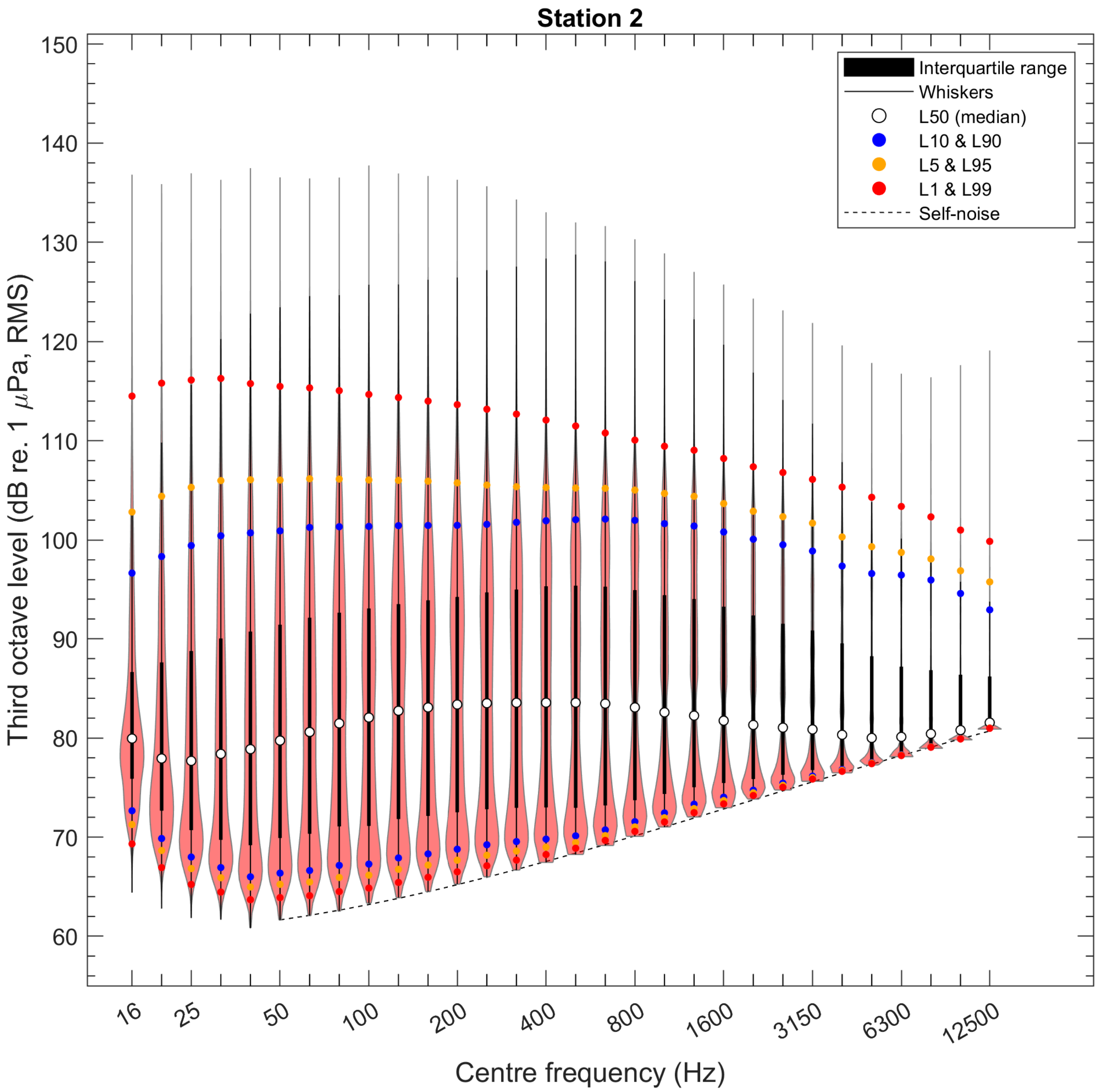

Third octave levels (TOLs) at station 2. The TOL distribution at each third octave band is represented with a violin plot overlaid with a boxplot and points at different exceedance levels (see legend). Boxplot whiskers extend to the lowest and highest data points observed within a distance of 1.5 times the interquartile range from the first and third quartiles, respectively.

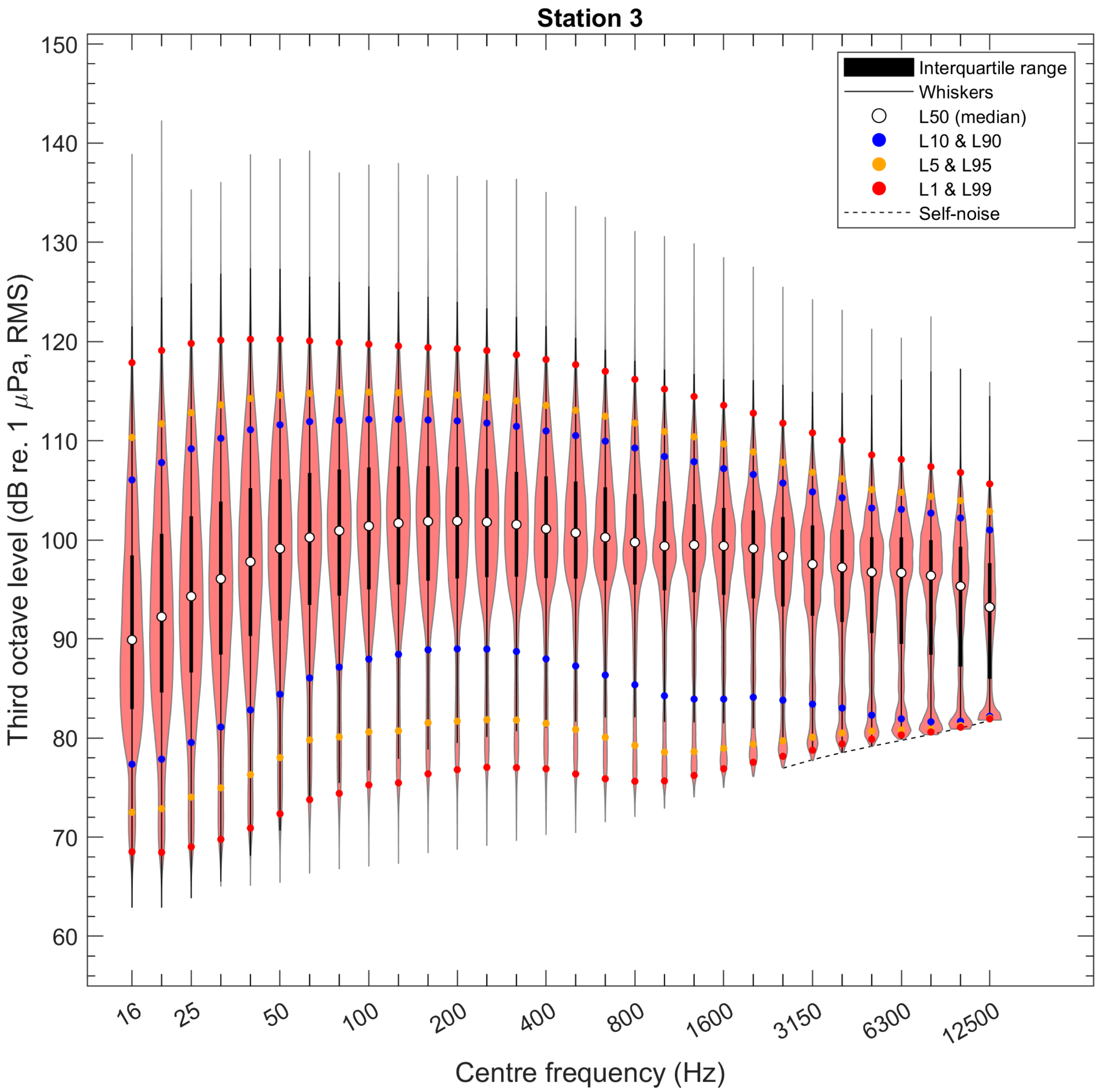

Third octave levels (TOLs) at station 3. The TOL distribution at each third octave band is represented with a violin plot overlaid with a boxplot and points at different exceedance levels (see legend). Boxplot whiskers extend to the lowest and highest data points observed within a distance of 1.5 times the interquartile range from the first and third quartiles, respectively.

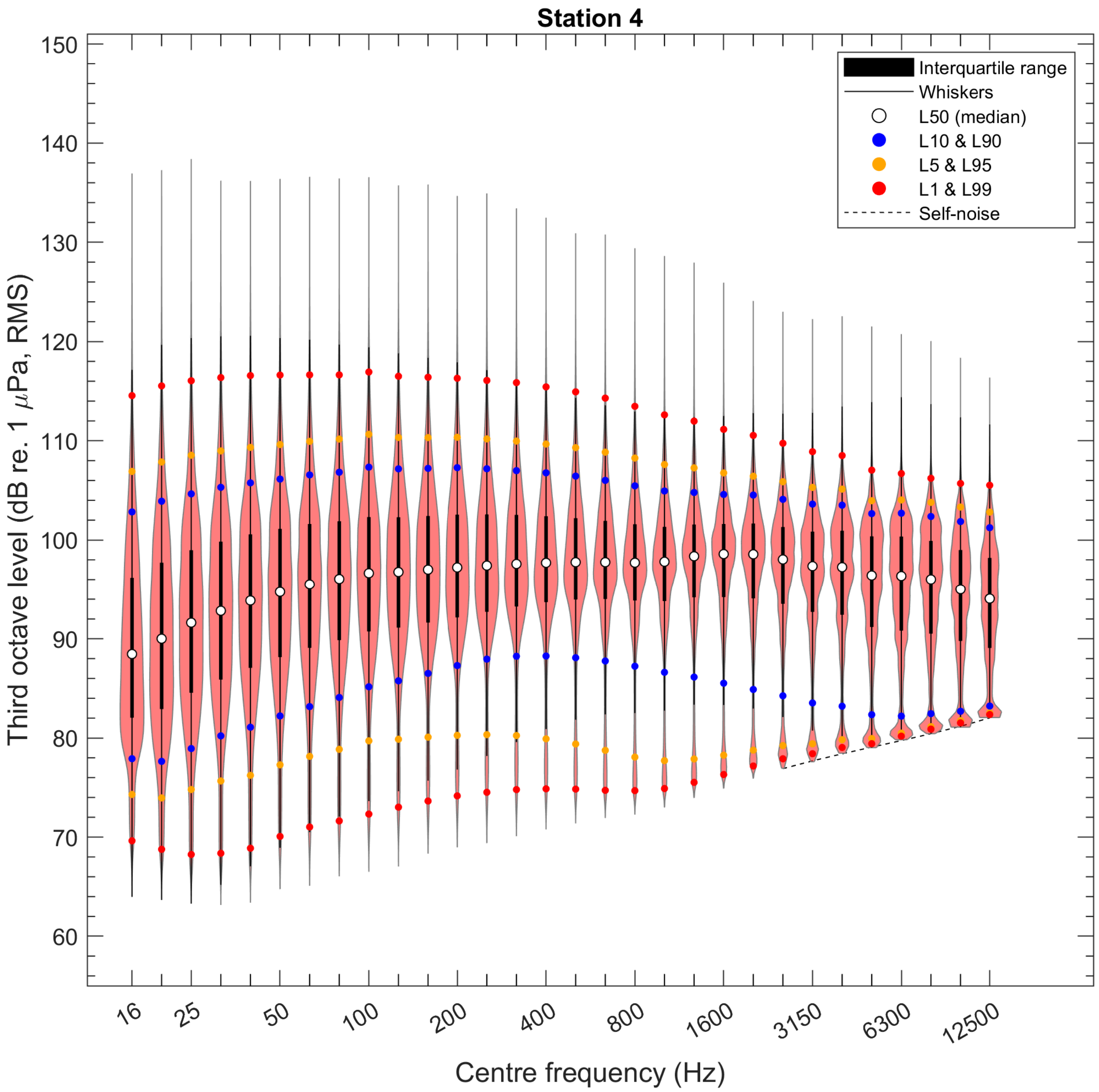

Third octave levels (TOLs) at station 4. The TOL distribution at each third octave band is represented with a violin plot overlaid with a boxplot and points at different exceedance levels (see legend). Boxplot whiskers extend to the lowest and highest data points observed within a distance of 1.5 times the interquartile range from the first and third quartiles, respectively.

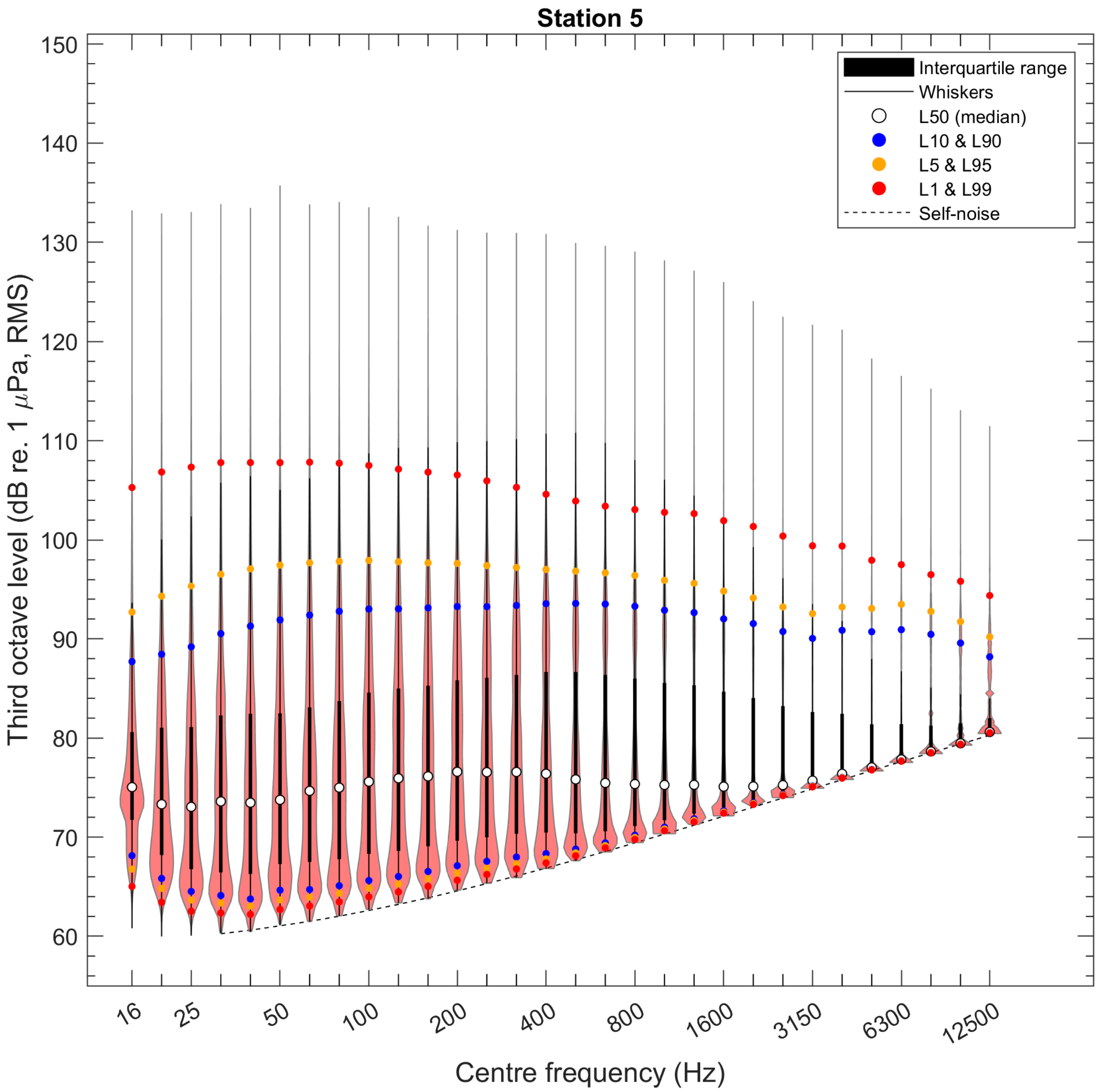

Third octave levels (TOLs) at station 5. The TOL distribution at each third octave band is represented with a violin plot overlaid with a boxplot and points at different exceedance levels (see legend). Boxplot whiskers extend to the lowest and highest data points observed within a distance of 1.5 times the interquartile range from the first and third quartiles, respectively.

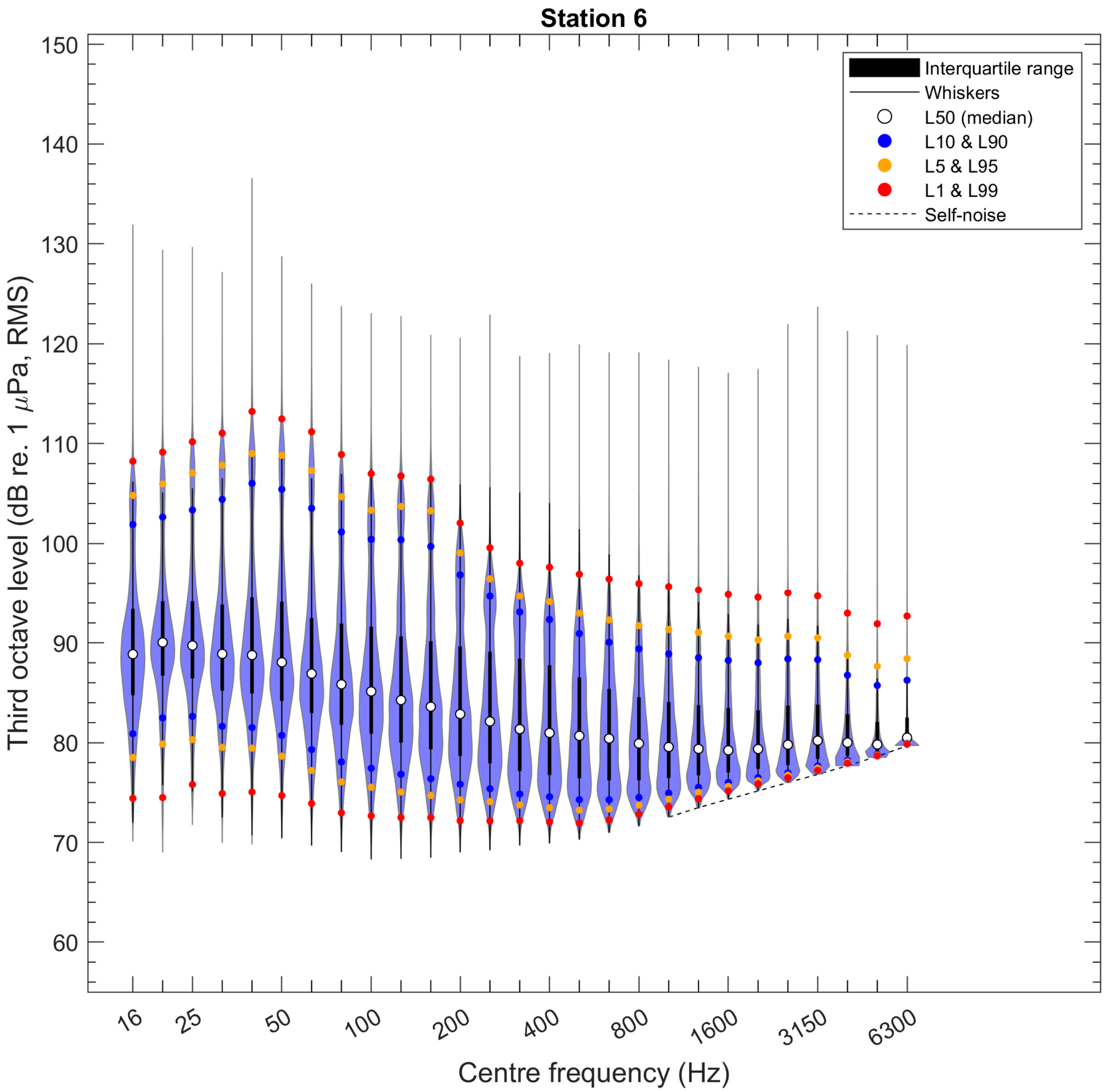

Third octave levels (TOLs) at station 6. The TOL distribution at each third octave band is represented with a violin plot overlaid with a boxplot and points at different exceedance levels (see legend). Boxplot whiskers extend to the lowest and highest data points observed within a distance of 1.5 times the interquartile range from the first and third quartiles, respectively.

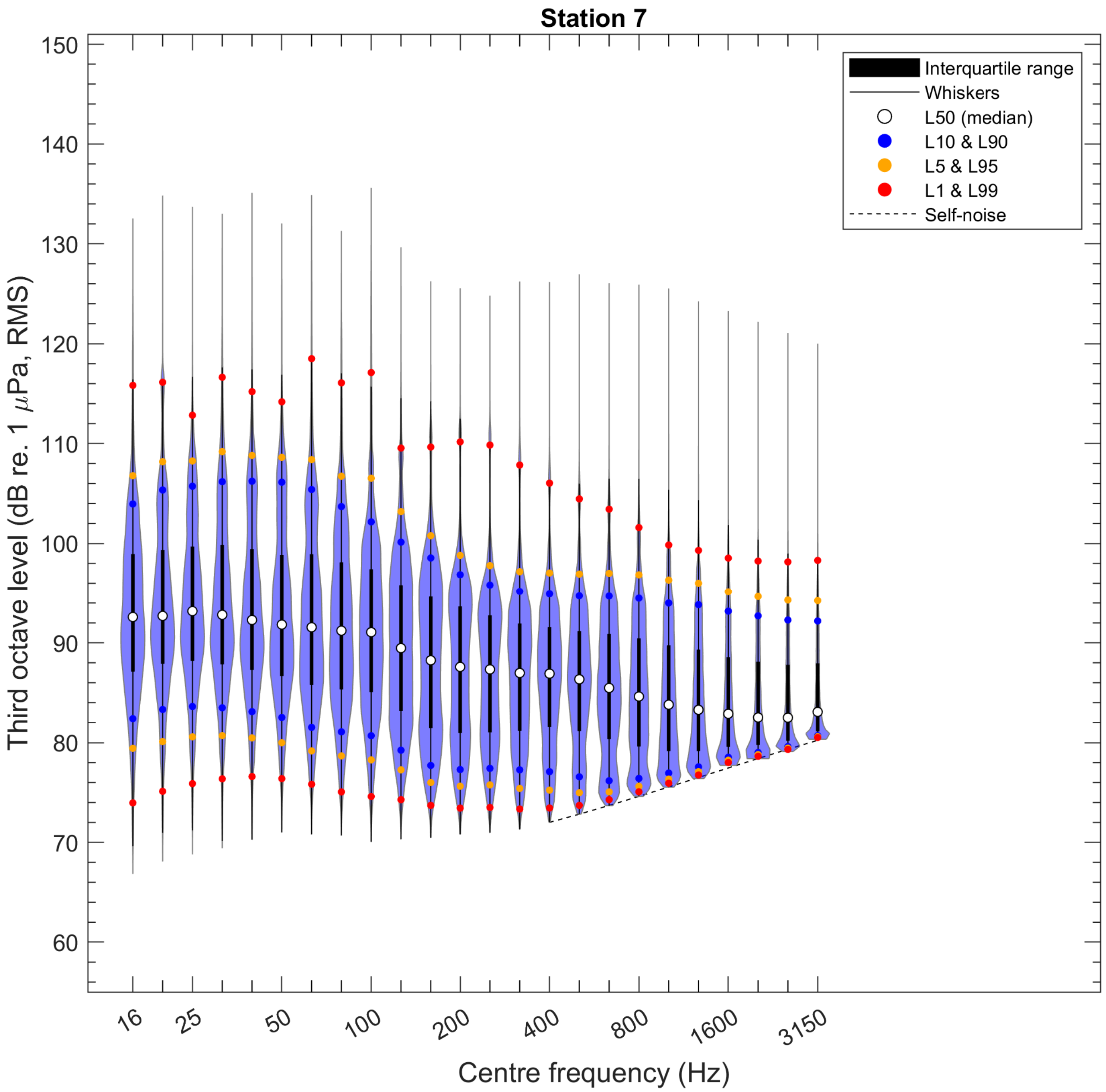

Third octave levels (TOLs) at station 7. The TOL distribution at each third octave band is represented with a violin plot overlaid with a boxplot and points at different exceedance levels (see legend). Boxplot whiskers extend to the lowest and highest data points observed within a distance of 1.5 times the interquartile range from the first and third quartiles, respectively.

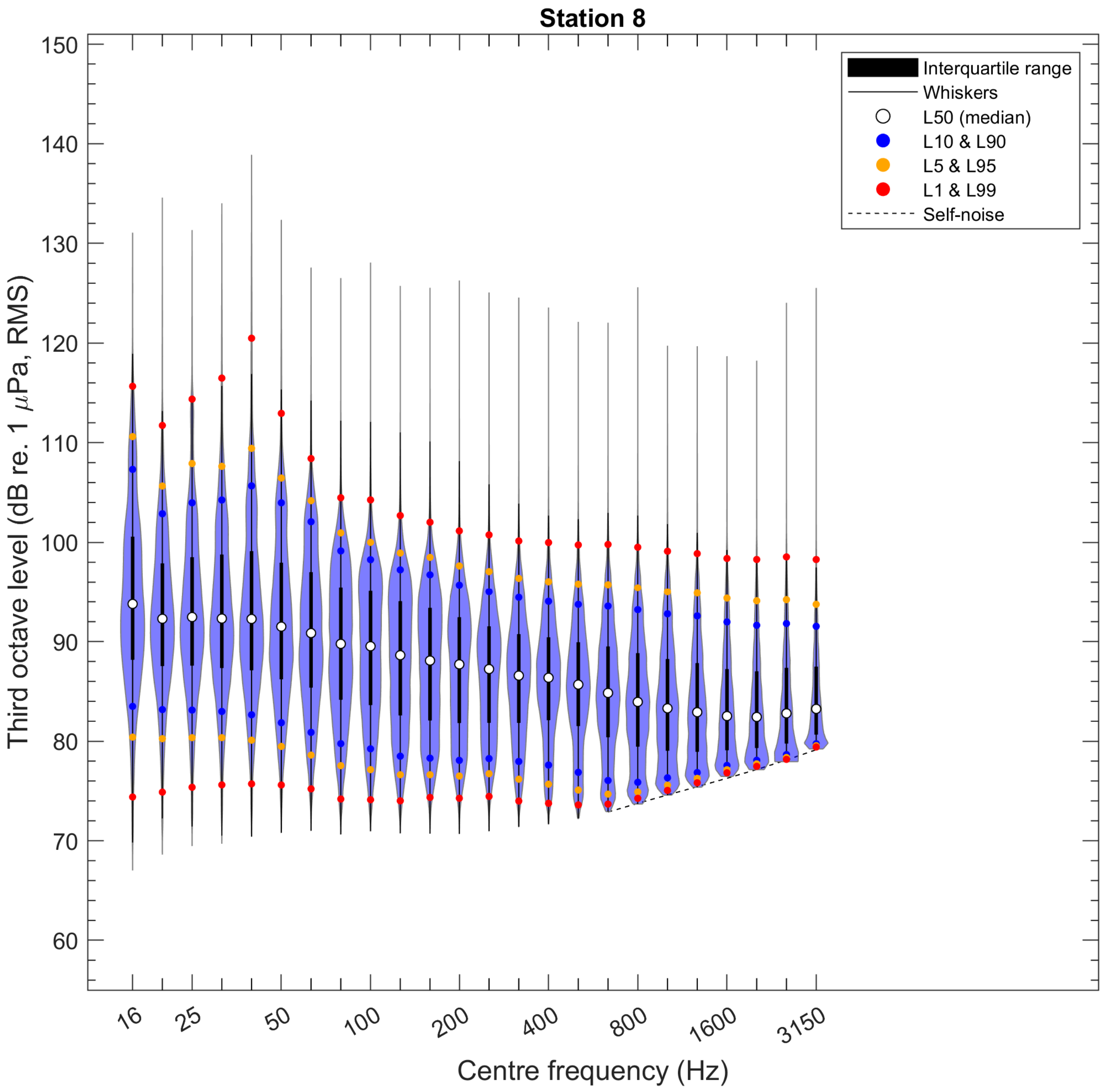

Third octave levels (TOLs) at station 8. The TOL distribution at each third octave band is represented with a violin plot overlaid with a boxplot and points at different exceedance levels (see legend). Boxplot whiskers extend to the lowest and highest data points observed within a distance of 1.5 times the interquartile range from the first and third quartiles, respectively.

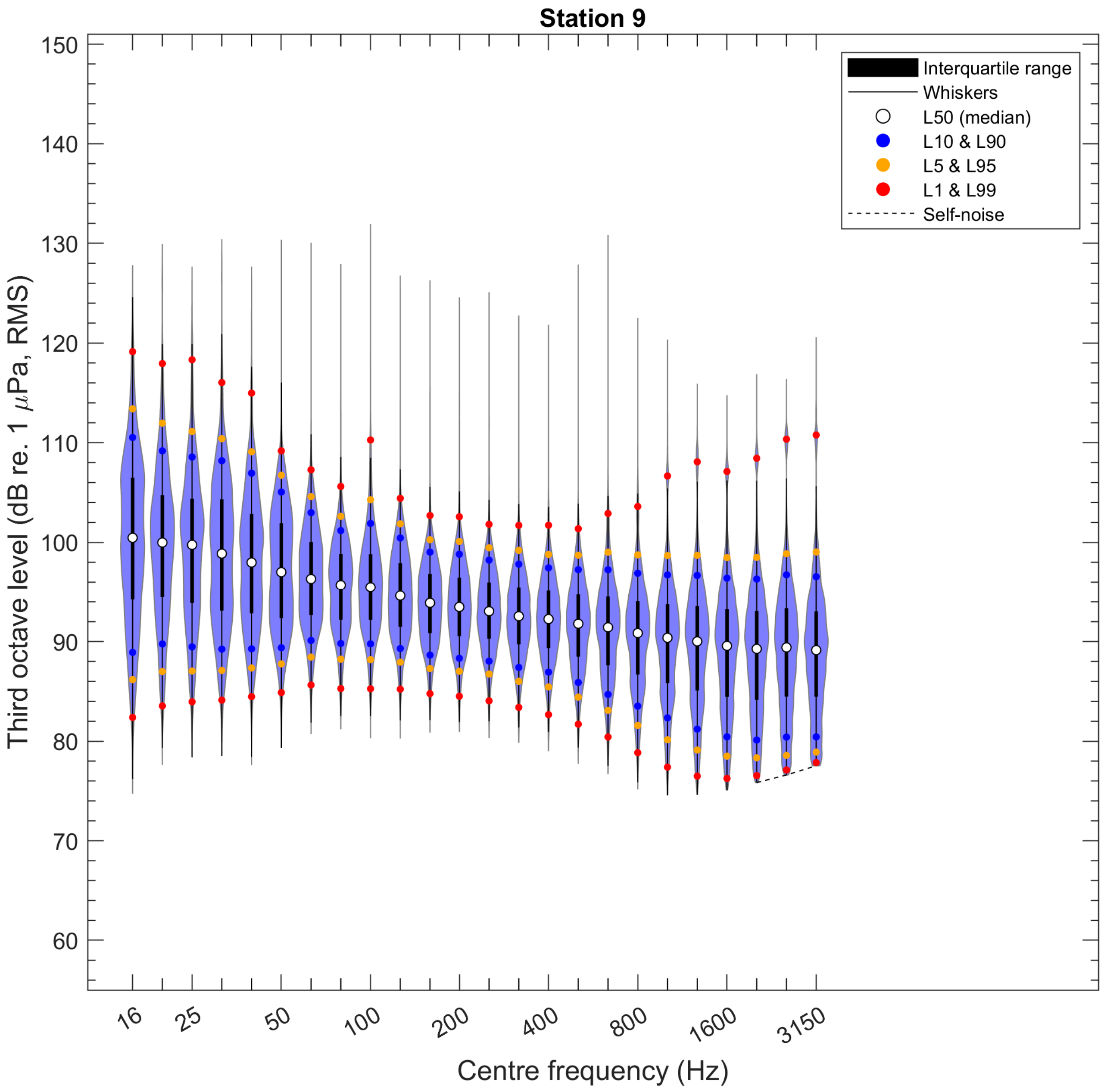

Third octave levels (TOLs) at station 9. The TOL distribution at each third octave band is represented with a violin plot overlaid with a boxplot and points at different exceedance levels (see legend). Boxplot whiskers extend to the lowest and highest data points observed within a distance of 1.5 times the interquartile range from the first and third quartiles, respectively.

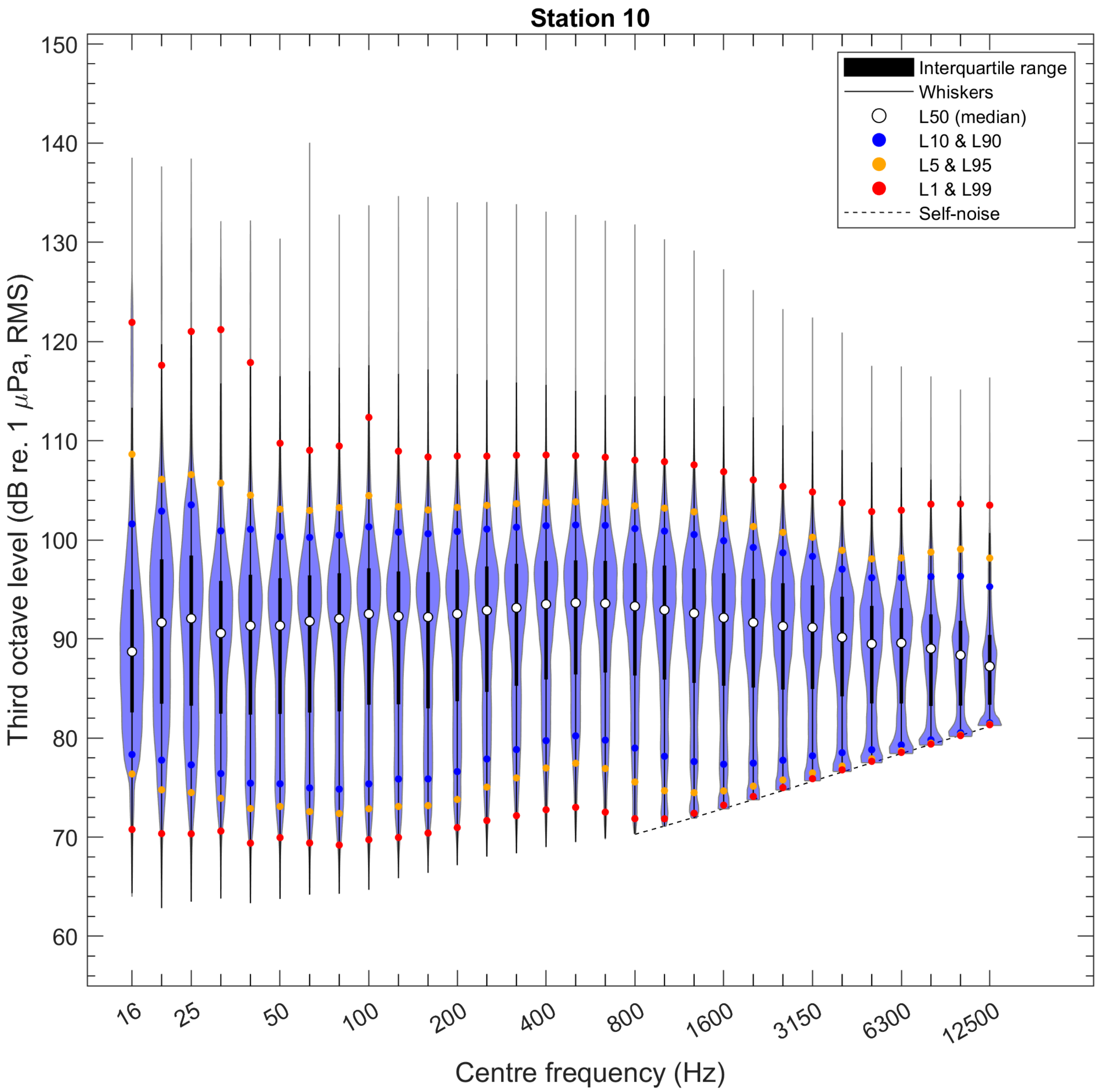

Third octave levels (TOLs) at station 10. The TOL distribution at each third octave band is represented with a violin plot overlaid with a boxplot and points at different exceedance levels (see legend). Boxplot whiskers extend to the lowest and highest data points observed within a distance of 1.5 times the interquartile range from the first and third quartiles, respectively.

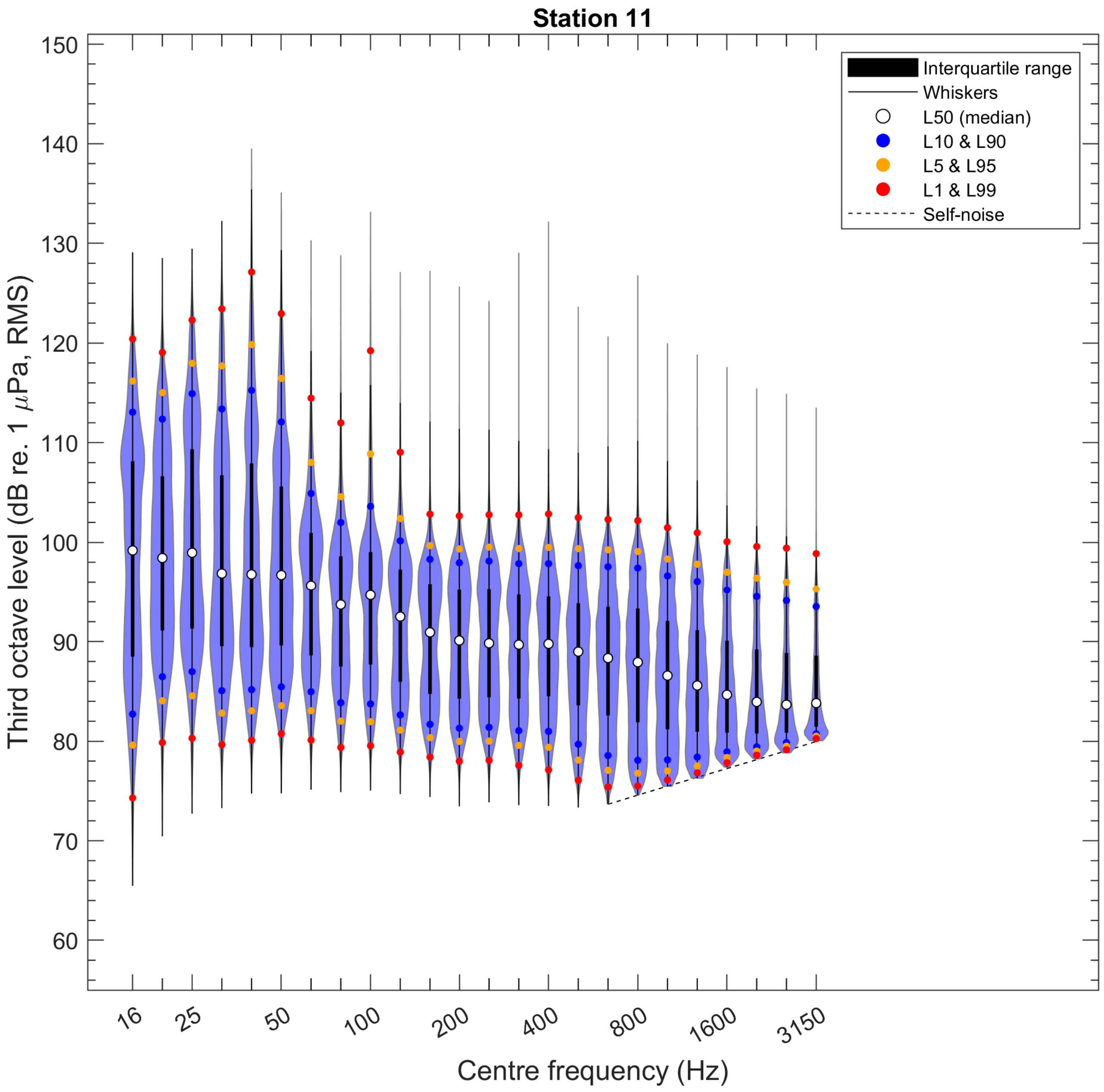

Third octave levels (TOLs) at station 11. The TOL distribution at each third octave band is represented with a violin plot overlaid with a boxplot and points at different exceedance levels (see legend). Boxplot whiskers extend to the lowest and highest data points observed within a distance of 1.5 times the interquartile range from the first and third quartiles, respectively.

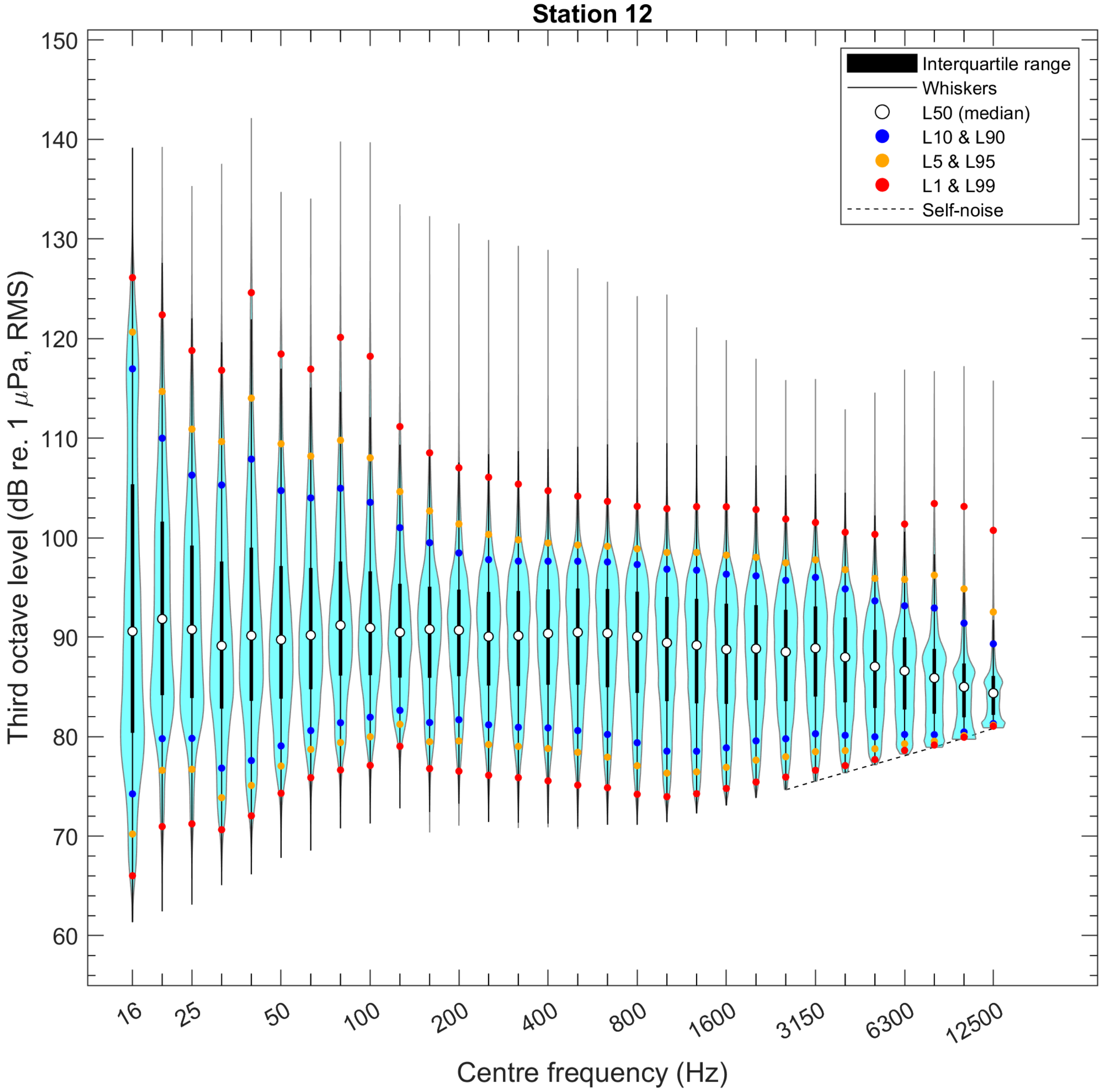

Third octave levels (TOLs) at station 12. The TOL distribution at each third octave band is represented with a violin plot overlaid with a boxplot and points at different exceedance levels (see legend). Boxplot whiskers extend to the lowest and highest data points observed within a distance of 1.5 times the interquartile range from the first and third quartiles, respectively.

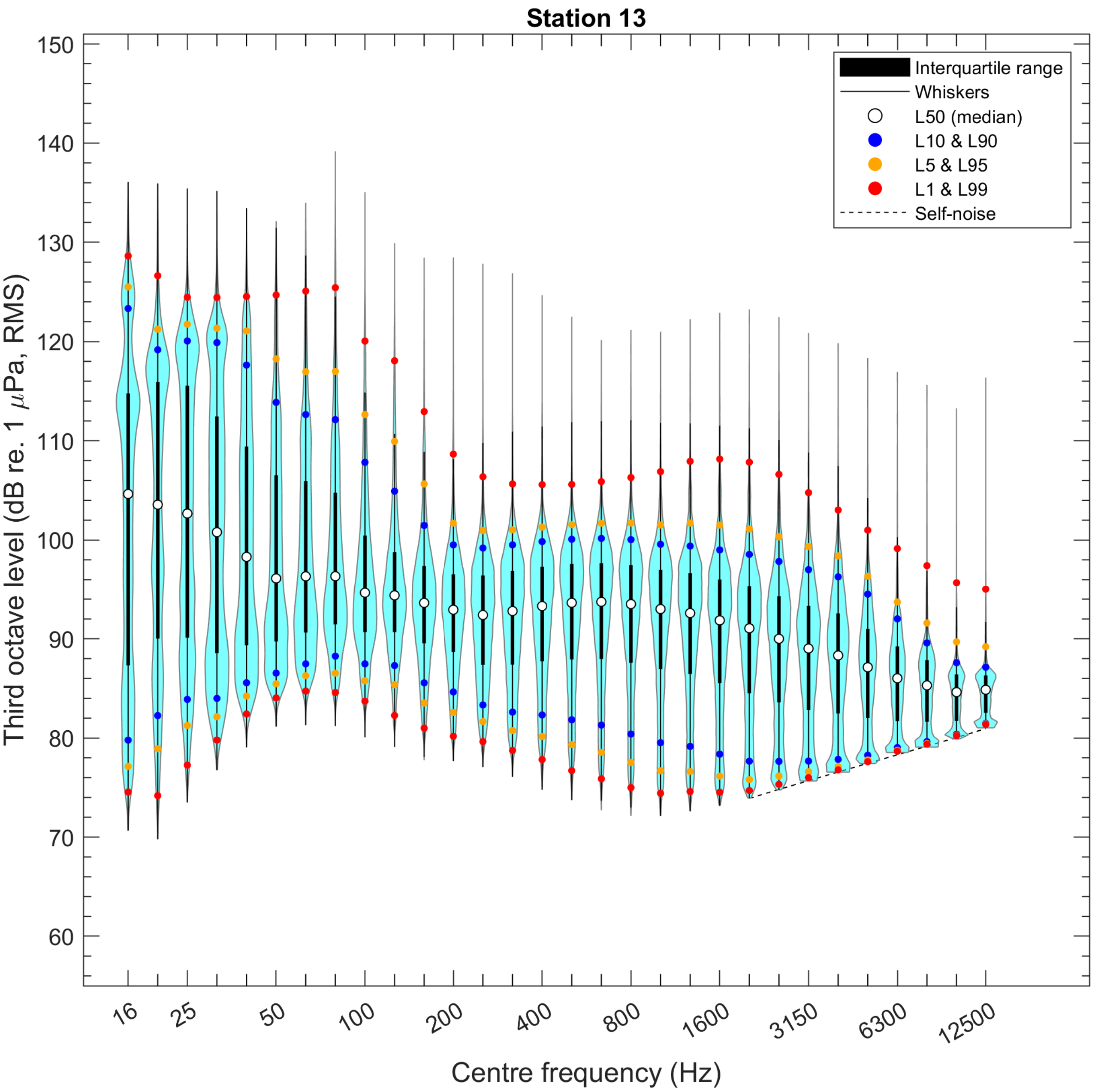

Third octave levels (TOLs) at station 13. The TOL distribution at each third octave band is represented with a violin plot overlaid with a boxplot and points at different exceedance levels (see legend). Boxplot whiskers extend to the lowest and highest data points observed within a distance of 1.5 times the interquartile range from the first and third quartiles, respectively.

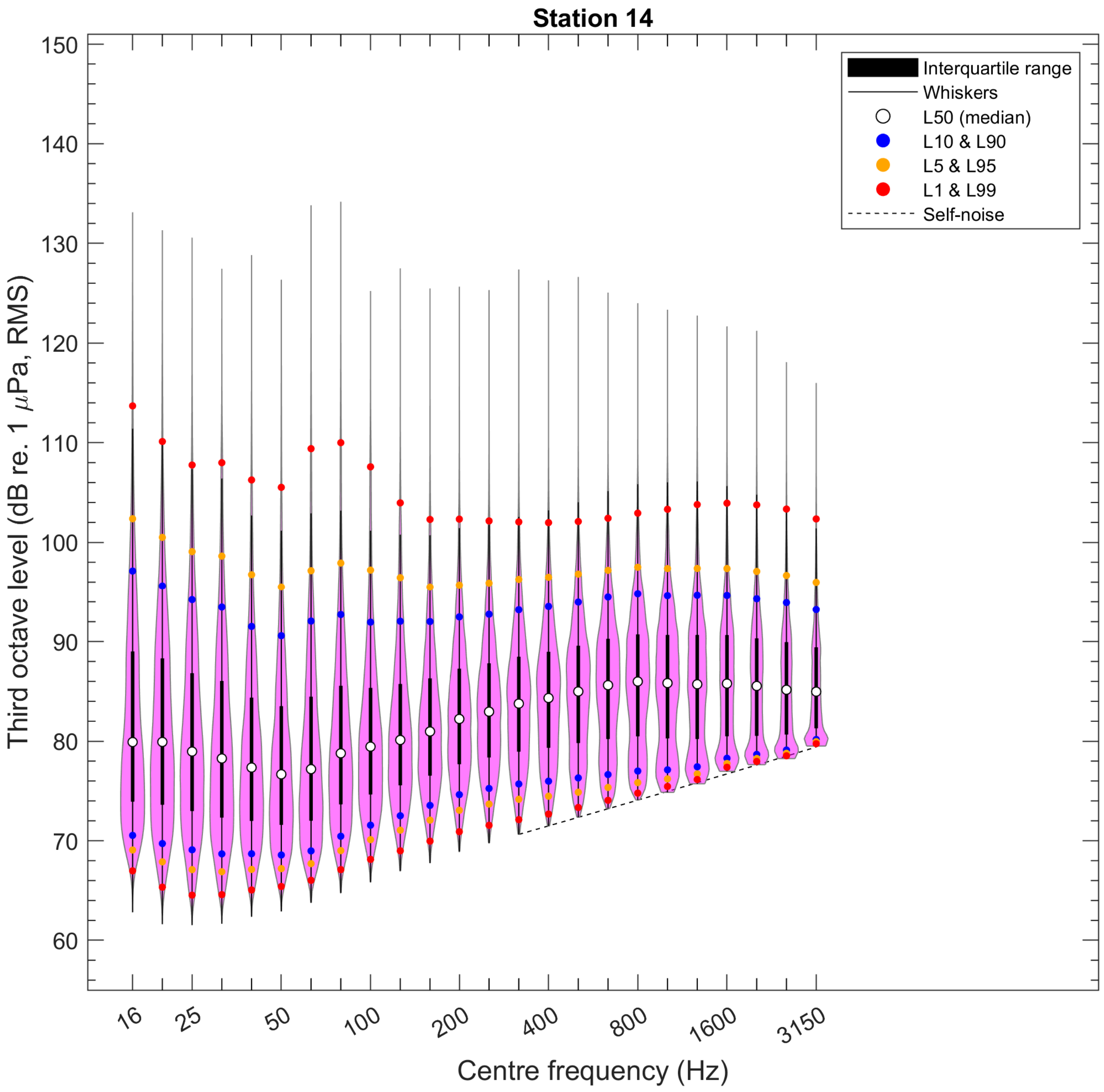

Third octave levels (TOLs) at station 14. The TOL distribution at each third octave band is represented with a violin plot overlaid with a boxplot and points at different exceedance levels (see legend). Boxplot whiskers extend to the lowest and highest data points observed within a distance of 1.5 times the interquartile range from the first and third quartiles, respectively.

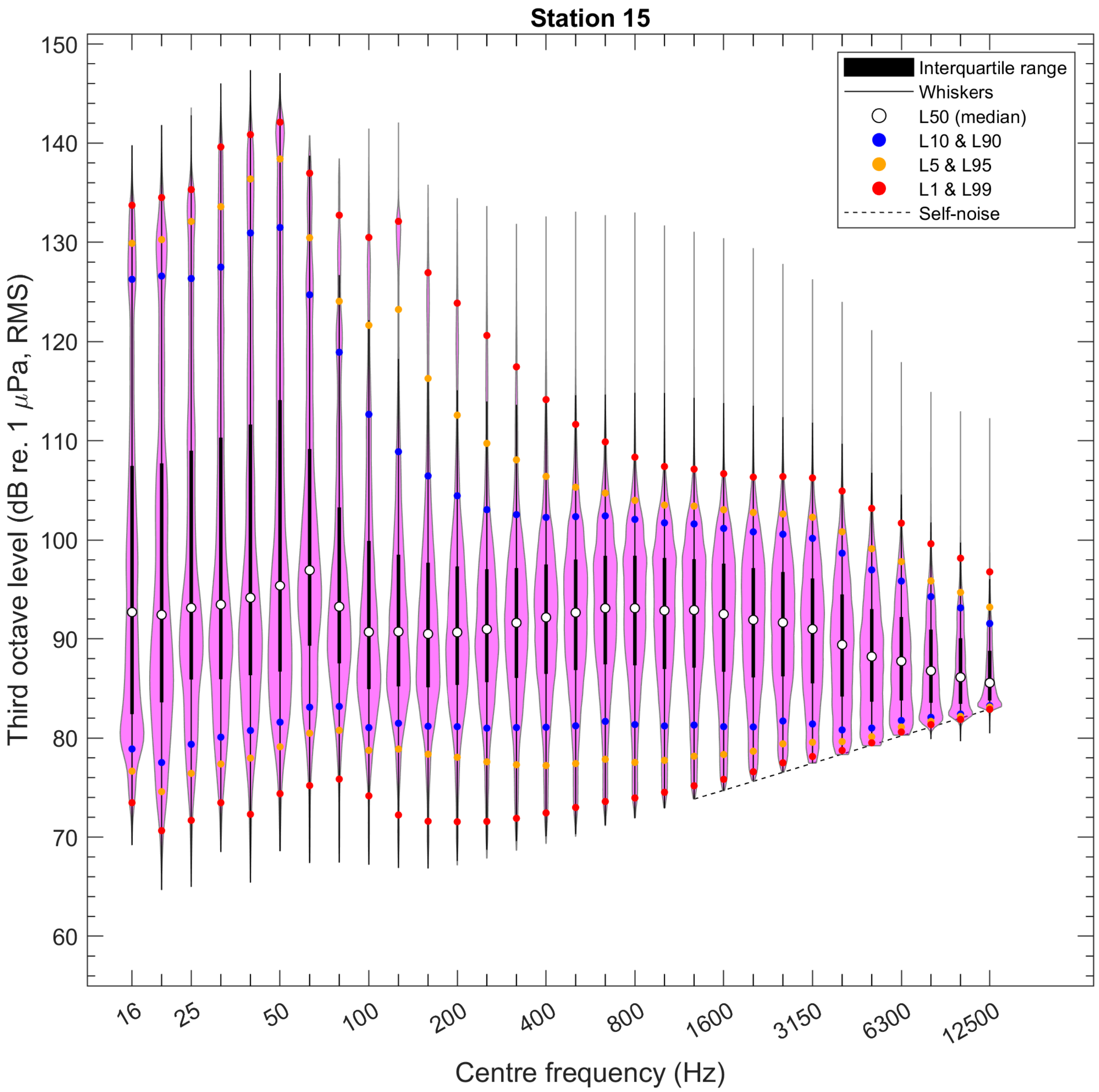

Third octave levels (TOLs) at station 15. The TOL distribution at each third octave band is represented with a violin plot overlaid with a boxplot and points at different exceedance levels (see legend). Boxplot whiskers extend to the lowest and highest data points observed within a distance of 1.5 times the interquartile range from the first and third quartiles, respectively.

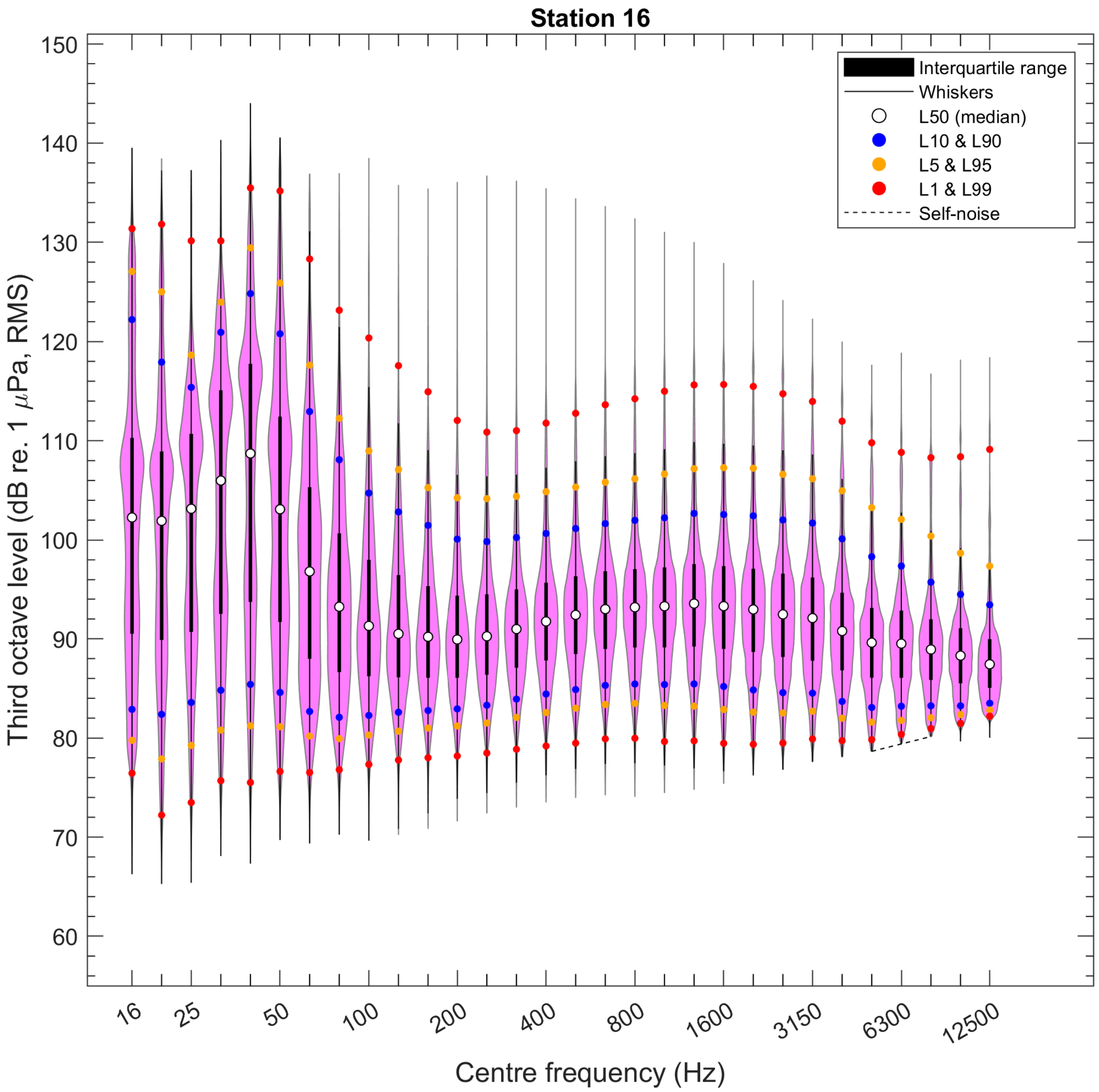

Third octave levels (TOLs) at station 16. The TOL distribution at each third octave band is represented with a violin plot overlaid with a boxplot and points at different exceedance levels (see legend). Boxplot whiskers extend to the lowest and highest data points observed within a distance of 1.5 times the interquartile range from the first and third quartiles, respectively.

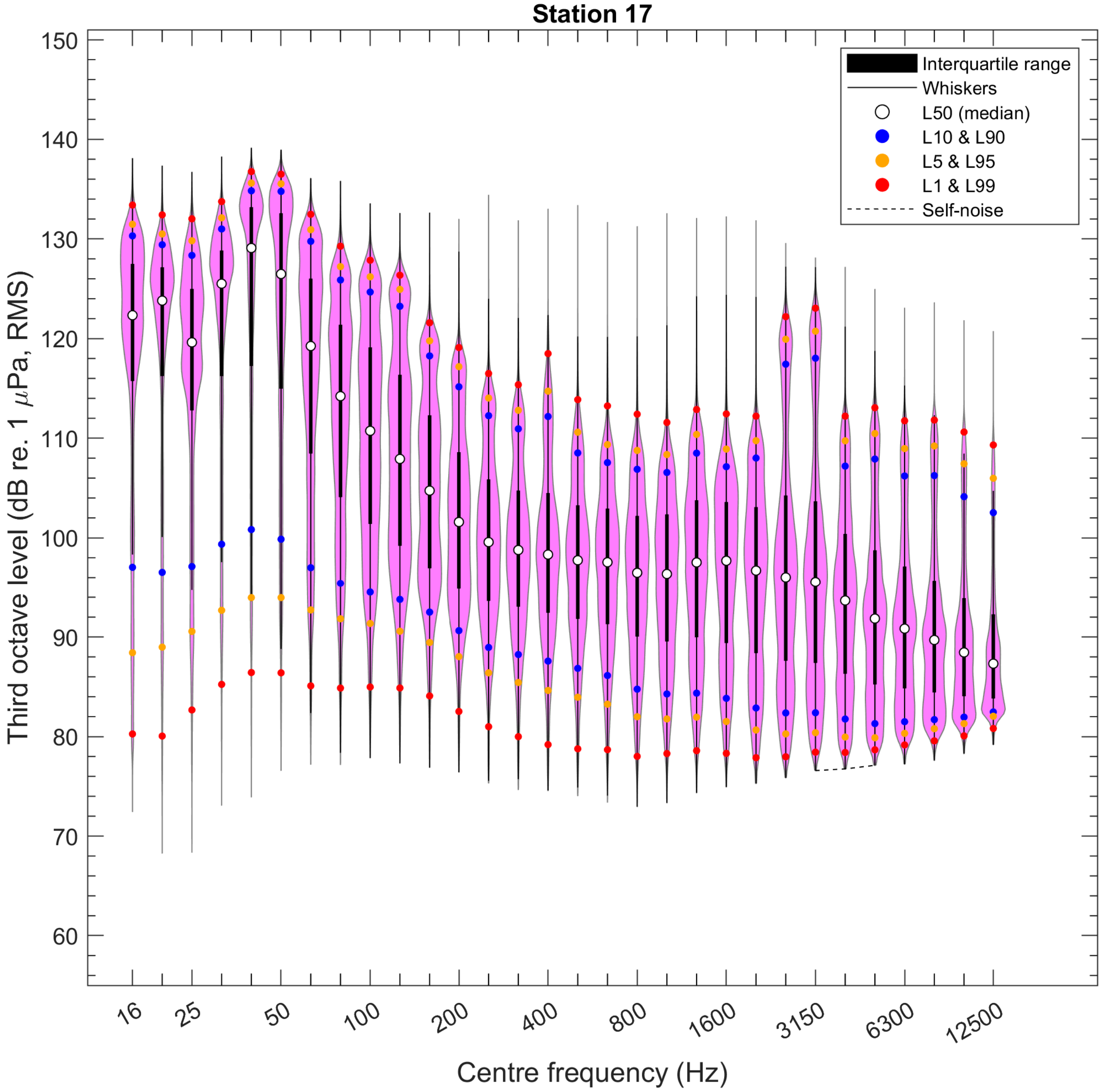

Third octave levels (TOLs) at station 17. The TOL distribution at each third octave band is represented with a violin plot overlaid with a boxplot and points at different exceedance levels (see legend). Boxplot whiskers extend to the lowest and highest data points observed within a distance of 1.5 times the interquartile range from the first and third quartiles, respectively.

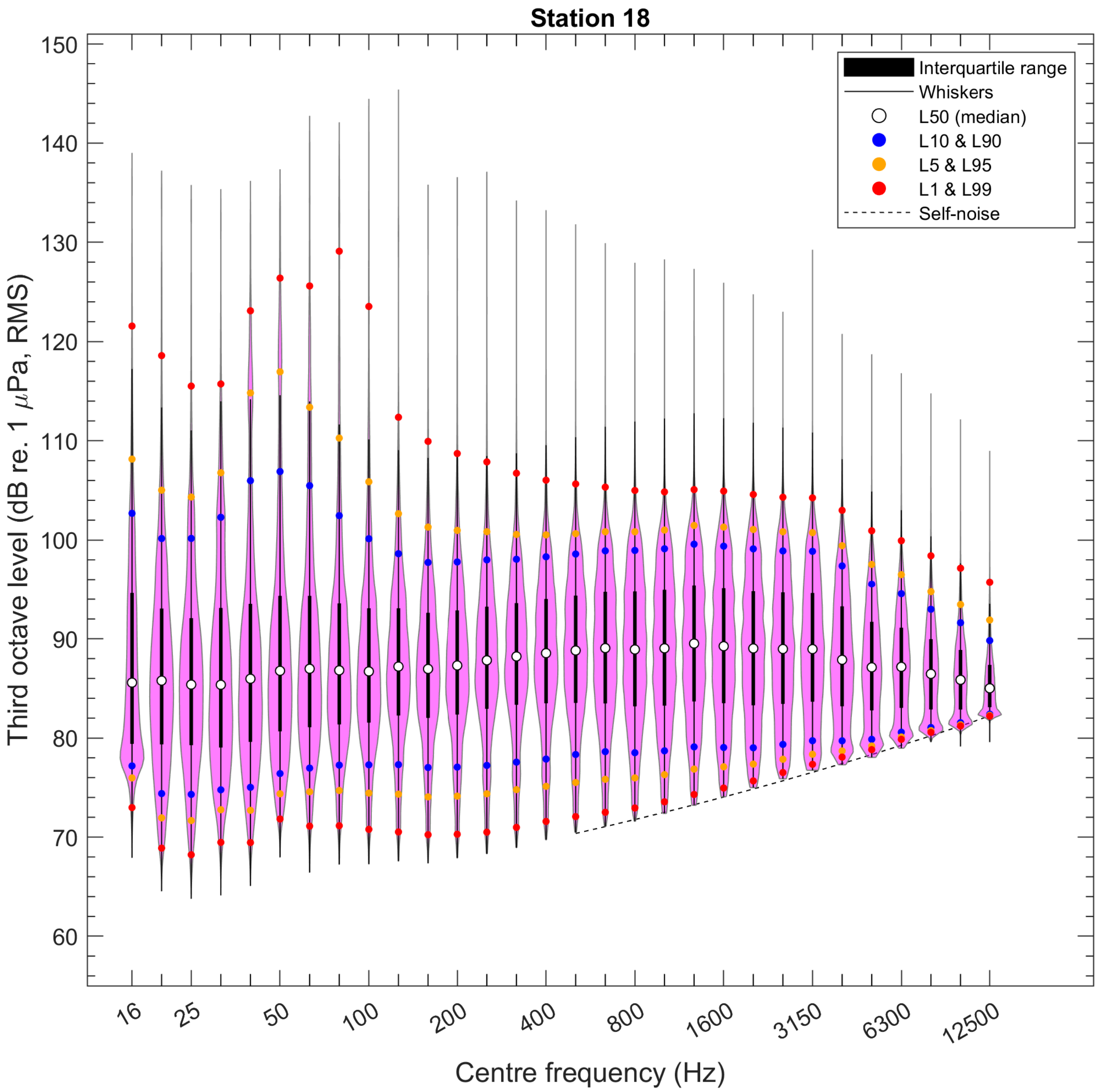

Third octave levels (TOLs) at station 18. The TOL distribution at each third octave band is represented with a violin plot overlaid with a boxplot and points at different exceedance levels (see legend). Boxplot whiskers extend to the lowest and highest data points observed within a distance of 1.5 times the interquartile range from the first and third quartiles, respectively.

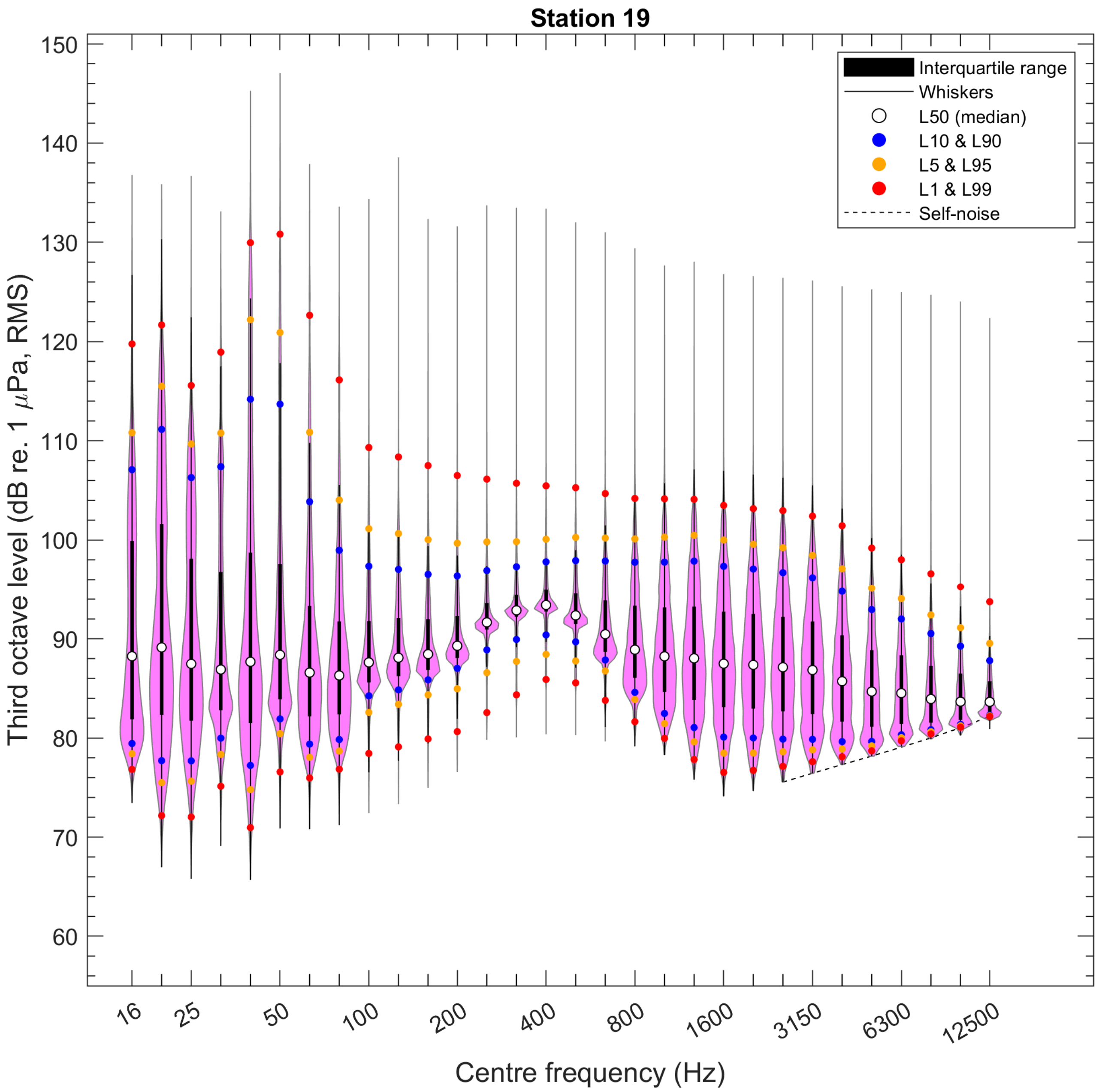

Third octave levels (TOLs) at station 19. The TOL distribution at each third octave band is represented with a violin plot overlaid with a boxplot and points at different exceedance levels (see legend). Boxplot whiskers extend to the lowest and highest data points observed within a distance of 1.5 times the interquartile range from the first and third quartiles, respectively.

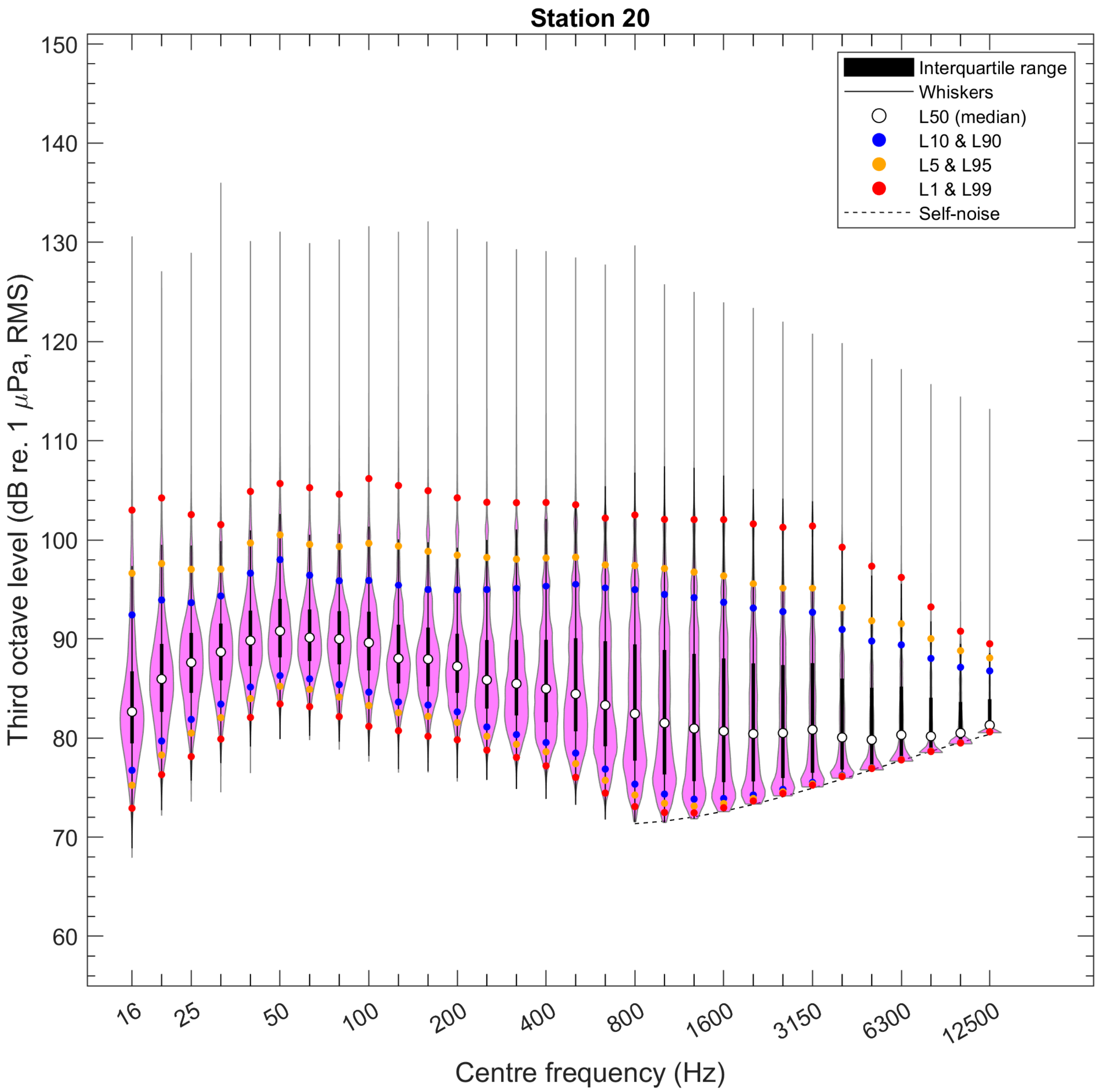

Third octave levels (TOLs) at station 20. The TOL distribution at each third octave band is represented with a violin plot overlaid with a boxplot and points at different exceedance levels (see legend). Boxplot whiskers extend to the lowest and highest data points observed within a distance of 1.5 times the interquartile range from the first and third quartiles, respectively.

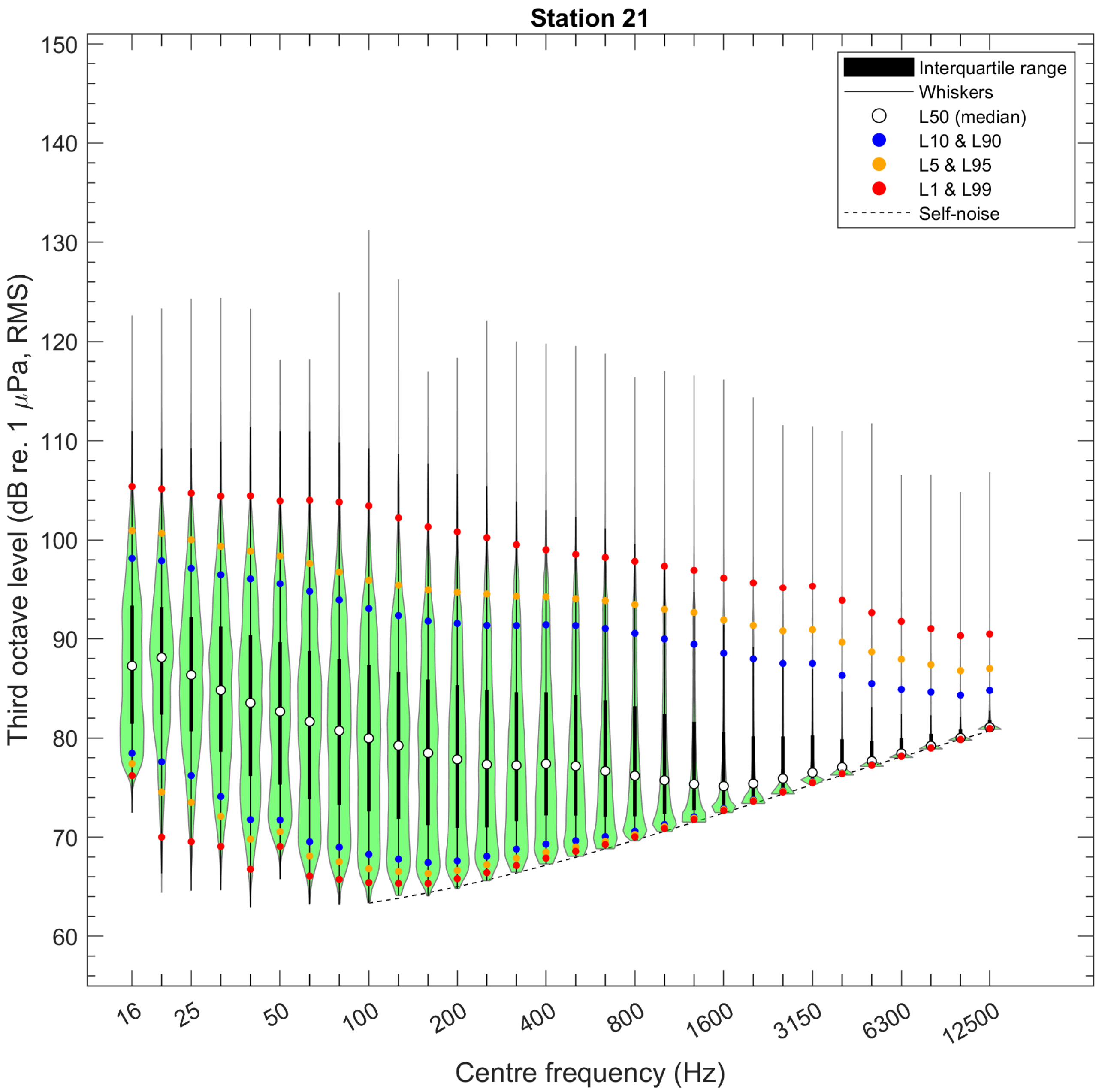

Third octave levels (TOLs) at station 21. The TOL distribution at each third octave band is represented with a violin plot overlaid with a boxplot and points at different exceedance levels (see legend). Boxplot whiskers extend to the lowest and highest data points observed within a distance of 1.5 times the interquartile range from the first and third quartiles, respectively.

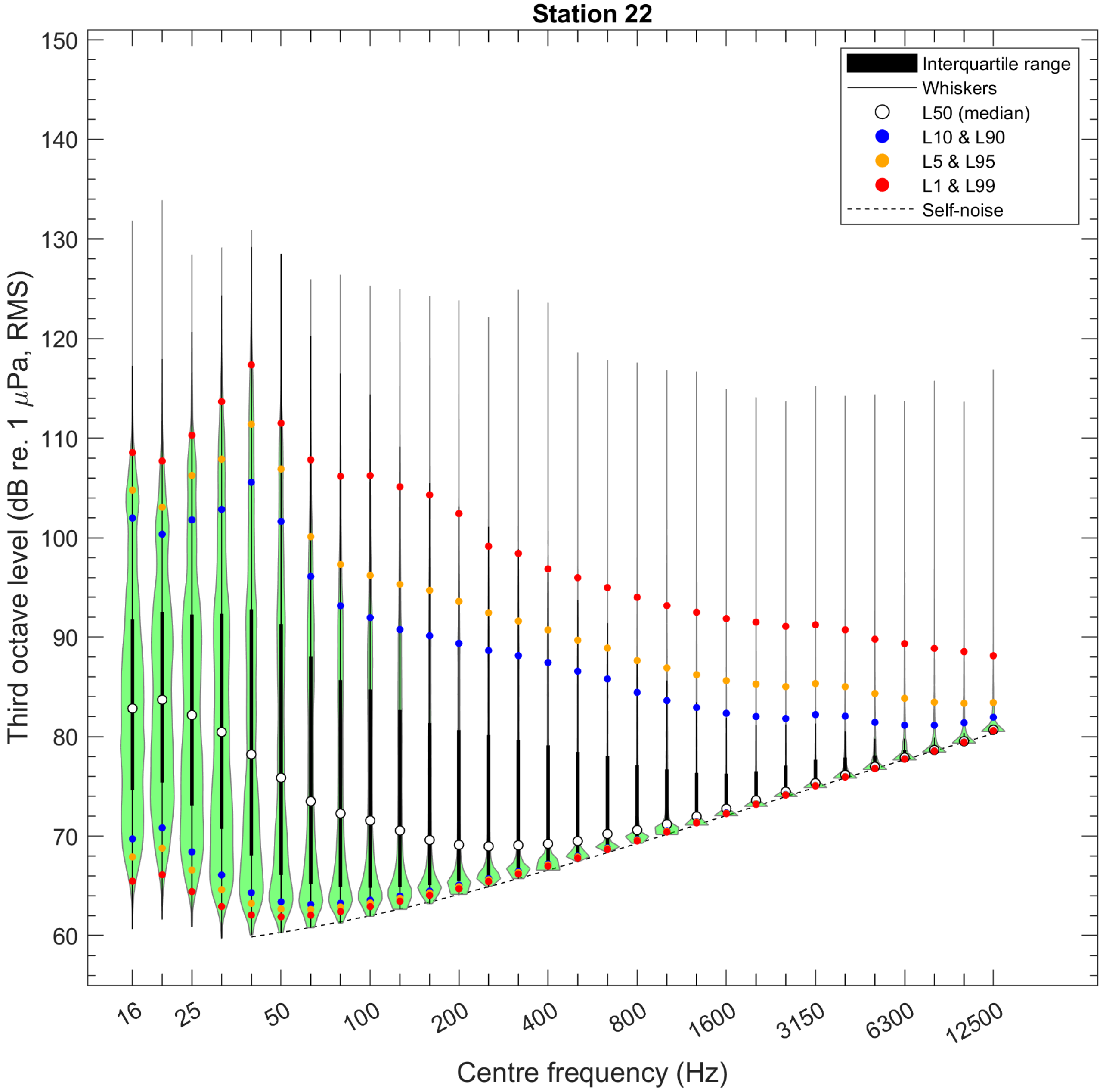

Third octave levels (TOLs) at station 22. The TOL distribution at each third octave band is represented with a violin plot overlaid with a boxplot and points at different exceedance levels (see legend). Boxplot whiskers extend to the lowest and highest data points observed within a distance of 1.5 times the interquartile range from the first and third quartiles, respectively.

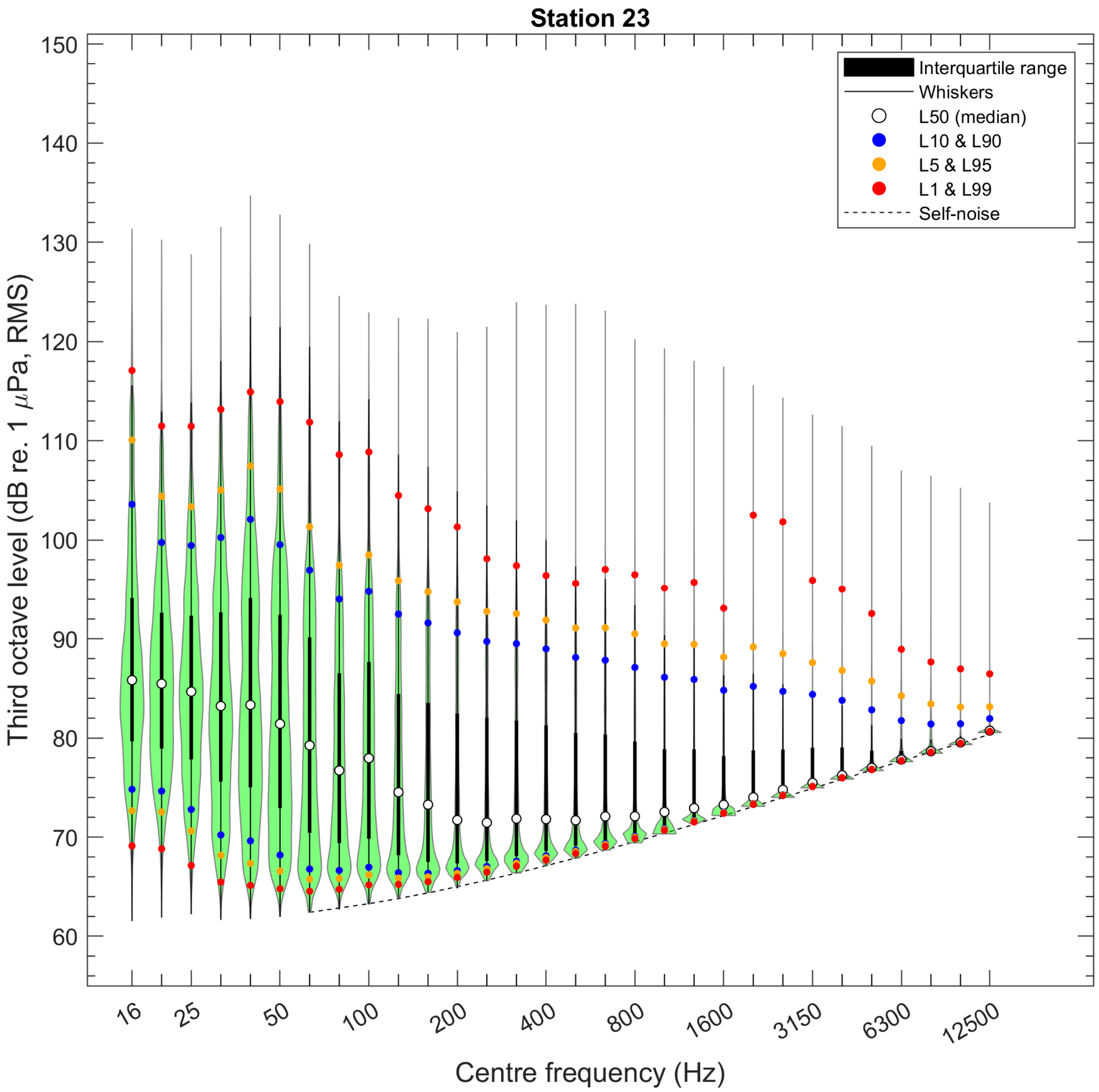

Third octave levels (TOLs) at station 23. The TOL distribution at each third octave band is represented with a violin plot overlaid with a boxplot and points at different exceedance levels (see legend). Boxplot whiskers extend to the lowest and highest data points observed within a distance of 1.5 times the interquartile range from the first and third quartiles, respectively.

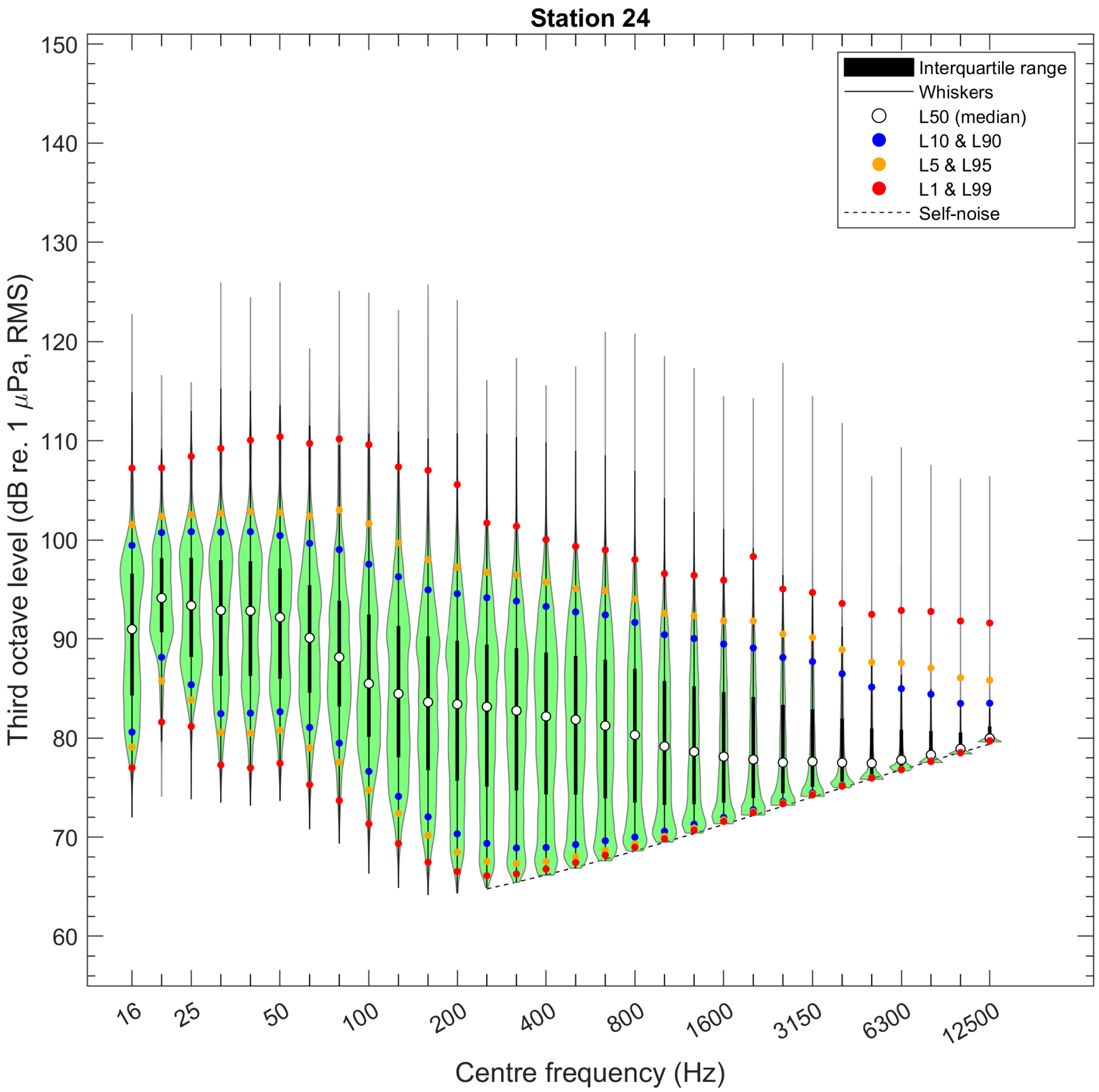

Third octave levels (TOLs) at station 24. The TOL distribution at each third octave band is represented with a violin plot overlaid with a boxplot and points at different exceedance levels (see legend). Boxplot whiskers extend to the lowest and highest data points observed within a distance of 1.5 times the interquartile range from the first and third quartiles, respectively.

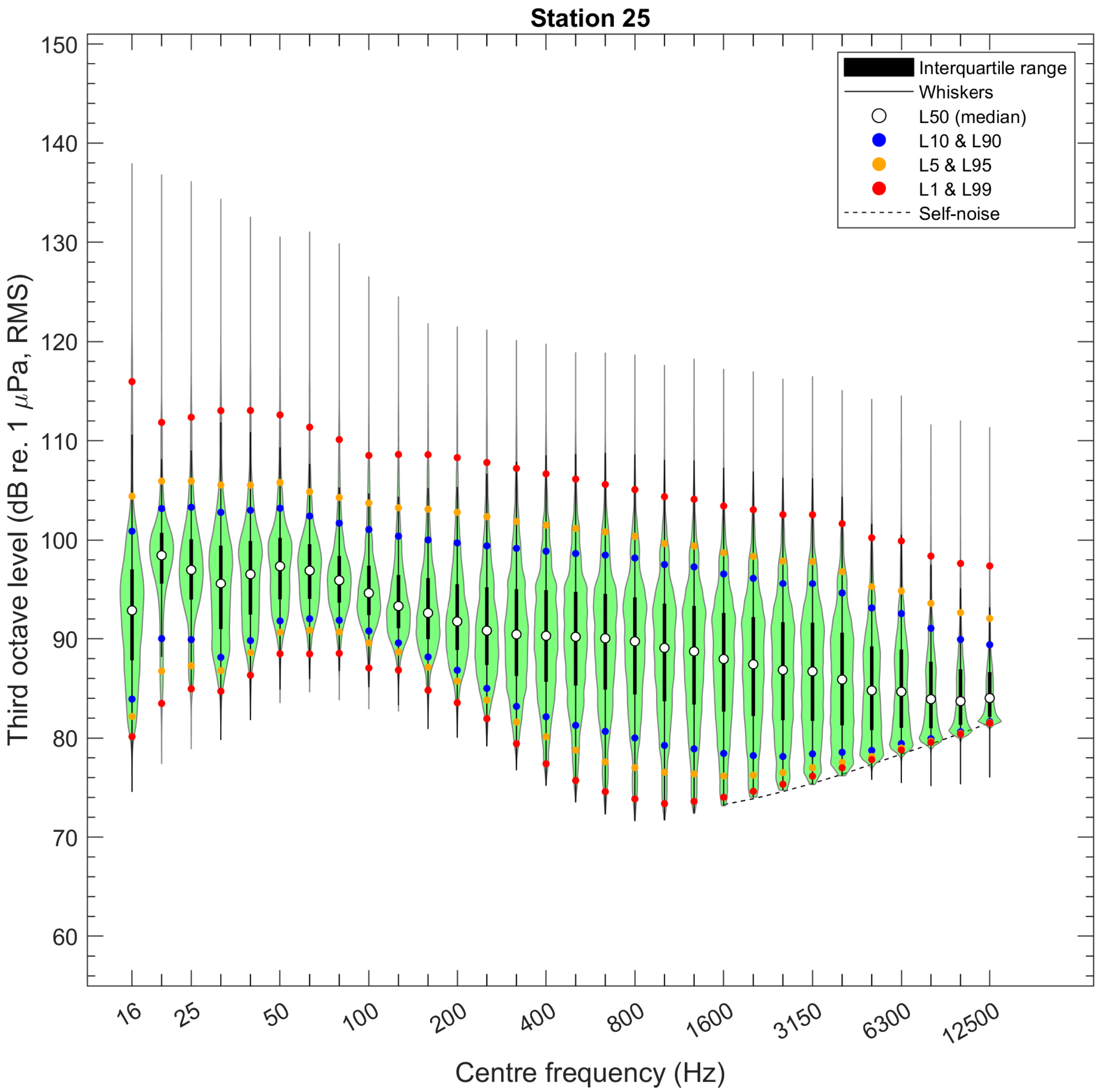

Third octave levels (TOLs) at station 25. The TOL distribution at each third octave band is represented with a violin plot overlaid with a boxplot and points at different exceedance levels (see legend). Boxplot whiskers extend to the lowest and highest data points observed within a distance of 1.5 times the interquartile range from the first and third quartiles, respectively.

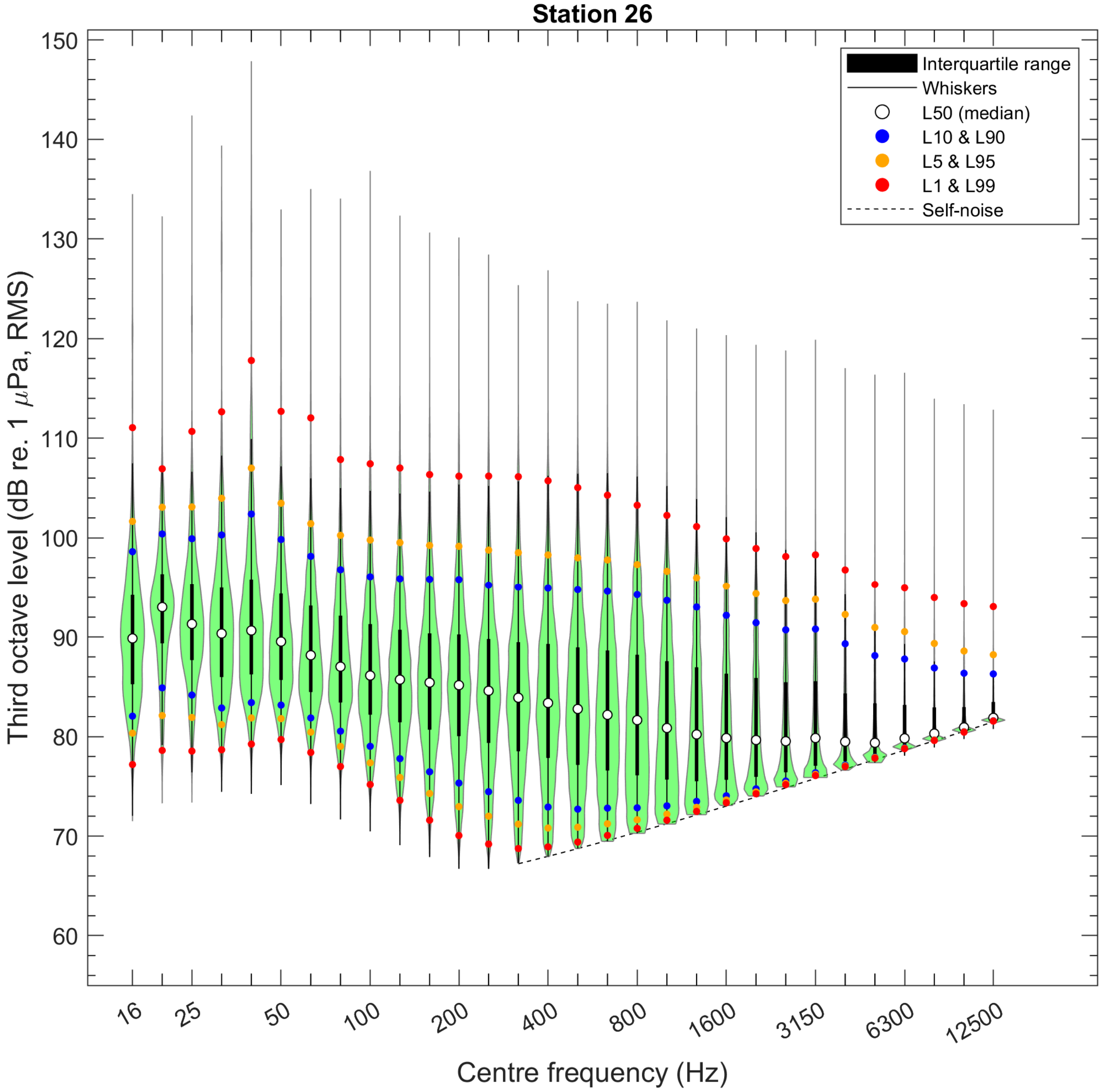

Third octave levels (TOLs) at station 26. The TOL distribution at each third octave band is represented with a violin plot overlaid with a boxplot and points at different exceedance levels (see legend). Boxplot whiskers extend to the lowest and highest data points observed within a distance of 1.5 times the interquartile range from the first and third quartiles, respectively.
